# Learning from the past to plan for the future: An historical review of the evolution of waste and resource management 1970–2020 and reflections on priorities 2020–2030 – The perspective of an involved witness

**DOI:** 10.1177/0734242X231178025

**Published:** 2023-09-21

**Authors:** David C Wilson

**Affiliations:** Imperial College London, London, UK

**Keywords:** Waste history, extending waste collection coverage, reduce, reuse, recycle, controlled recovery and disposal, nine development bands, developing countries, informal sector recycling, integrated sustainable waste management

## Abstract

Improving waste and resource management (WaRM) around the world can halve the weight of plastics entering the oceans, significantly mitigate global heating and contribute directly to 12 of 17 sustainable development goals (SDGs). Achieving such results demands understanding and learning from historical evolution of WaRM. The baseline is 1970, prior to environmental legislation. Early steps in the Global North focused on the ‘technical fix’ within strictly enforced legal frameworks, first bringing hazardous wastes and municipal solid wastes (MSW) under control, then gradually ramping up environmental standards. Using modern technologies to the Global South often failed due to institutional and financial constraints. From 1990, focus switched to integrating technical and governance aspects: local institutional coherence, financial sustainability, provider inclusivity, user inclusivity, national legislative and policy framework. The Global North rediscovered recycling, using policy measures to promote segregation at source; this relied on new markets in emerging economies, which had largely disappeared by 2020. The Global South is making progress on bringing wastes under control, but around 2.7 billion people lack access to waste collection, while ~40% of collected MSW is open dumped or burned – a continuing global waste emergency. So, much remains to be done to move further towards a circular economy. Three policy priorities are critical for all countries: access to sustainable financing, rethinking sustainable recycling and worldwide extended producer responsibility with teeth. Extending services to unserved communities (SDG11.6.1) requires a people-centred approach, working with communities to provide both quality services and decent livelihoods for collection and recycling workers.

## Introduction

For most of human development, the environment was the ‘global commons’ (Hardin, 1968); any emissions or wastes from resource extraction, material production, agriculture, manufacturing processes, distribution and use, as well as human wastes, could simply be released without charge to air, water or land. Legislation to control the discharge of human wastes, and to require the collection of solid wastes in cities, was first introduced in the 19th century to protect public health. However, the disposal of municipal, industrial and hazardous solid wastes remained uncontrolled – essentially ‘out of sight, out of mind’ (Wilson, 2007) until the last quarter of the 20th century. Uncontrolled emissions to air were more ‘visible’; London smog prompted control of household burning of solid fuels in the UK 1956 *Clean Air Act*, with similar legislation elsewhere in developed countries.

The volume of resource use and thus also of emissions and wastes has increased exponentially since around 1950 (the ‘great acceleration’) (Steffen et al., 2015). Between 1950 and 1970, both world population and the percentage living in urban areas had increased by 50%, to 3.8 billion and 57%, respectively. Consumerism was beginning as living standards began to rise. Environmental pressures were increasing: Rachel Carson’s *Silent Spring (Carson, 1982)* drew attention to the global issue of pollution from persistent pesticides such as DDT and catalysed the environmental movement. As cities expanded, people were impacted more by uncontrolled dumpsites originally located beyond the city limits, particularly those containing hazardous wastes. All this resulted in the introduction of comprehensive environmental legislation in developed countries from the 1970s, covering pollution to air, water and wastes to land. Hence, the selection of 1970 is the baseline for this paper.

I started work in the sector shortly after enactment of the UK 1974 *Control of Pollution Act* and have focused over the years on issues of policy and planning for both municipal solid wastes (MSW) and industrial hazardous wastes, and the evidence base to underpin that. So, I have been fortunate both to have a ‘front-row seat’ to witness and also to contribute, in a modest way, to the rapid evolution of waste and resource management (WaRM) since then.

### Why it is important to understand and learn from the recent past (1970–2020)

Solid waste management (SWM) has traditionally been a ‘Cinderella’ subject, receiving even less attention than other areas of environmental pollution such as wastewater or air pollution. SWM is an essential utility service underpinning modern society, but unlike water and wastewater, electricity and gas supply, telephone and, more recently, internet services, it has never achieved general recognition as such. Attaining political priority has tended to rise and fall, with historic up-turns following cholera epidemics in the 19th century (Girling, 2005; Tulchinsky and Varavikova, 2014); high-profile cases of uncontrolled hazardous waste sites, such as Love Canal in the US (Brown, 1979; LaGrega et al., 1994) or Lekkerkerk in the Netherlands (Kingsbury and Bingham, 1992), in the 1970s; and a wide array of news-worthy incidents when things go wrong and waste piles up in the streets (e.g. in Naples in 2010 or Beirut in 2016), or a landslip at an uncontrolled dumpsite kills tens or hundreds of people (Zhang et al., 2020).

Some of the most developed countries moved rapidly to bring wastes under control in the 1970s, and started ‘ramping up’ technical standards of control in the 1980s. Others have followed a similar progression, although both the exact path taken and the degree of time-lag have varied widely. I first noted this tendency towards a step-by-step approach when invited to identify ‘*priorities for waste management in the 1990s*’ (Wilson, 1988), and returned regularly to the subject, adding later steps, over the years (Wilson, 1993, 1999a, 2007; UNEP and ISWA, 2015).

**Figure 1.**
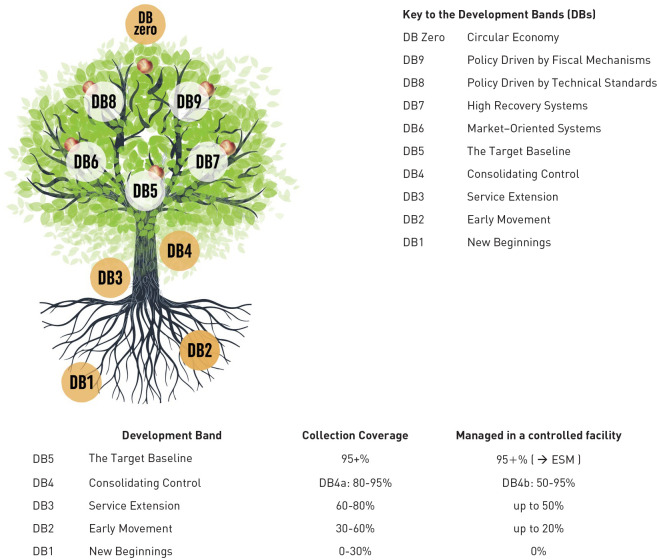
The Nine Development Bands (9DBs) theory of waste and development: Showing the ‘9DBs tree’, the key to the development bands, and progress through the early DBs. The roots and trunk of the tree represent early development bands in the development of a municipal solid waste management (MSWM) system (DB1–DB4), as rates of collection coverage and management in a controlled facility gradually increases. The top of the trunk, emerging from DB4 into the new ‘target baseline’ DB5, marks compliance with SDG indicator 11.6.1, ‘universal’ (95+%) waste collection and controlled recovery or disposal. DB6 –> DB8 and DB7 –> DB9 represent two distinct historical routes through the ‘leafy branches’ as MSWM evolves more into waste and resource management (WaRM), with (95+%) full control or environmentally sound management (ESM) in recovery and disposal achieved in DB6 and DB7, and a focus on the 3Rs in DB8 and DB9. DB zero sits on top of the tree, representing the ultimate aspiration of a ‘zero waste’ circular economy. Source: Whiteman et al. (2021). Figure © Andrew Whiteman (Graphics: Ecuson Studio). 3Rs: reduce, reuse, recycle; DB: development band; ESM: environmentally sound management; SDG: sustainable development goal.

My international consultancy work has been extremely varied, but I often summarise the early decades as helping countries and/or cities to identify and implement the next appropriate steps in developing their own sustainable systems for managing MSW or hazardous waste. The key ‘tools of the trade’ are to identify a country’s current starting point and build from there; and to understand the journeys already taken by other countries and adapt those lessons learned to the specific local situation. When one looks at ‘modern waste and resource management’ in high-income countries today, it is important to remember that it has taken them 50 years to get to where they are now from their 1970 baseline; other countries are scattered at different points along the route, with many of the least developed countries still striving to extend waste collection to most of their urban populations and so not yet at that 1970 baseline. These observations form the basis for the ‘nine development bands’ (9DBs), our recent global theory of waste and development (Figure 1) (Whiteman et al., 2021).

Much of my work over the last 15 years has focused on developing such analytical tools (Wilson et al., 2015a) and distilling higher level lessons for the future (Scheinberg et al., 2010b; Wilson and Scheinberg, 2010; Wilson et al., 2012b, 2013). When I led preparation of the inaugural *Global Waste Management Outlook* (GWMO) (UNEP and ISWA, 2015; Wilson and Velis, 2015), part of our brief was to raise the political profile of SWM, particularly in relation to developing countries. We documented various groups of drivers (Wilson, 2007; Marshall and Farahbakhsh, 2013), including the public health risks from uncollected wastes, the local environmental damage from uncontrolled disposal and open burning, and the huge indirect ‘costs of inaction’ to society. We emphasised the resource value in the waste, which has the potential to provide decent livelihoods to millions, and thus contribute to a ‘just transition’ from SWM into inclusive WaRM, and noted resource scarcity as a related driver towards a circular economy. We highlighted the global environmental benefits from climate mitigation, both by reducing direct (largely methane) emissions and indirectly through reduction, reuse and recycling (3Rs). However, despite our best efforts, if I had been asked early in 2017 if we had succeeded in the original ambition of elevating the political profile of the sector, I would have said ‘No!’.

That changed in 2017/2018 when Sir David Attenborough finally broke through to global consciousness on the tragedy of plastics entering the oceans (Rapid Transition, 2019). Yes, marine plastics were already on the scientific agenda (e.g. Jambeck et al., 2015; Velis et al., 2017), but I could not have predicted the scale to which global action and funding to tackle plastics pollution has since ‘taken off’. Whilst current data and scientific understanding do not give a clear picture, my best estimate is that extending basic waste collection and controlled disposal to all would cut in half the weight of plastics reaching the oceans (CIWM and Wasteaid UK, 2018). With the agreement to develop a legally binding global instrument on plastics pollution (UNEA, 2022a), one can argue that SWM/WaRM has now finally got firmly onto the international agenda (Silva-Filho and Velis, 2022).

### This paper

I first discussed the proposal for the International Solid Waste Association (ISWA) to set up a peer-reviewed journal with Jens Aage Hansen at a conference in Gatlinburg, Tennessee in October 1981, and volunteered to write this paper as a contribution to celebrating *Waste Management & Research* (WM&R)’s 40th anniversary in 2023. The aim is to identify and review the evolution in WaRM which I have witnessed since the 1970s; and to use that and my work on global priorities over the last 15 years to reflect on how that continuing evolution should be shaped in the next decade. My basic thesis is that it is necessary to understand how WaRM has evolved in the past to plan confidently for the future and to avoid ‘reinventing the wheel’. I hope that this paper can contribute both by acting as a conduit to earlier experiences, including the ‘grey’ literature, but also to encourage researchers not to neglect early work when conducting literature reviews.

#### Audience

The intended audience can be divided into two. The detailed historical review in Parts A and B of the paper (see Methodology) is aimed primarily at a professional and academic audience; while the reflections in Part C on present and future priorities going forward are aimed at a much broader audience including decision-makers and their advisors. Indeed, many readers may wish to begin with the forward looking Part C, and then ‘dipping back’ into the detailed historical material.

#### Focus

The topic is huge for one paper; so, the focus is on some of the bigger picture changes over time; other authors in the WM&R 40th anniversary series address in detail specific topics which I can only touch on. The scope necessarily reflects my own career, so includes both MSW and hazardous waste; the journey of developed countries in the so-called ‘Global North’ (in particular the UK and EU, but with some mention also of the United States, Canada, Japan, Australia and New Zealand) from end-of-pipe waste management through the 3Rs towards a circular economy; the journeys of emerging economies (China, former Soviet Union and Eastern European countries); the continuing struggles of many other developing countries in the ‘Global South’ to take even the early steps to bring their wastes under control; and some of the successes and failures along the way.

It was a happy coincidence that I happened to stumble into a career in waste management in the 1970s. Its rapid evolution over the years, the transition into WaRM and the struggles of developing countries to make progress have all helped to keep me interested. It is another coincidence that, thanks to plastics pollution, the early 2020s appears to be a ‘tipping point’, when my ‘baby’ has at last ‘come of age’, and is emerging onto the world stage as a global priority. So I hope that this paper is timely.

## Methodology

This paper documents recent history through the perspective of an actively involved ‘witness’ who has also been a ‘participant’ making a modest contribution. I first viewed myself in this way when I was invited to participate in a Witness Seminar, defined in the preface to the resulting book as ‘*a specialized form of oral history, where several individuals associated with a particular set of circumstances or events are invited to meet together to discuss, debate and agree or disagree about their memories*’ (Jones and Tansey, 2015).

I have used two complementary sources for the historical review. The first is my own memories and experiences – I also use my career progression as part of the ‘thread’ to provide a coherent narrative. I use the first person when I wish to emphasise my personal perspective at the time under discussion; otherwise, the third person is used. To confirm my recollections and to provide proper documentation, I make extensive use of my personal library, including grey literature reports and proceedings of conferences at which I presented; and have also corresponded with past and present colleagues to fill in some of the gaps.

The second approach is the more conventional semi-systematic review of literature. I have conducted many searches on a wide range of specific topics, to search for both older and the more recent literature. I have used Scopus to search the peer-reviewed literature, and internet searches for the ‘grey’ literature. I have also made extensive use of ‘snowballing’, beginning with a key source and using their reference list to look back in time and citations of the source to identify more recent relevant work.

### Analytical framework

What analytical framework to use in the paper? That apparently simple question posed a dilemma, because one of the main things that has evolved over the last 50 years, and which I have contributed to, is precisely the lens through which WaRM has been analysed. So, which parts of that should that be treated as part of the evolving story, and which as comparative tools for evaluating ‘progress’ over time? Two recent analytical tools serve as the latter, both based on the concept of a step-by-step approach.

**Figure 2.**
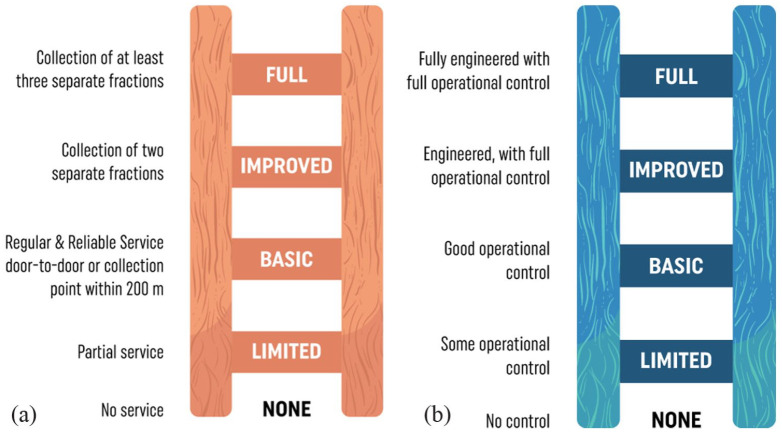
Ladders of service/control. (a) Service ladder for waste collection and (b) Example control ladder for recovery and disposal facilities: the control levels are defined separately for landfill, incineration and other recovery - this example is for landfill. Each ladder shows a step-wise progression through five levels of service or control. For the purposes of meeting SDG indicator 11.6.1, collection coverage must meet at least the ‘basic’ level of service (regular collection of mixed waste); and recovery and disposal facilities the ‘basic’ levels of control. The ‘improved’ and ‘full’ levels of collection service include separation at source to facilitate both the quantity and quality of recycling; while ‘full’ control of recovery and disposal facilities correspond to environmentally sound management (ESM) (SDG 12.4). Source: Whiteman et al. (2021). Figure © Andrew Whiteman and David C. Wilson (Graphics: Ecuson Studio). The requirements for each level have been summarised for the graphical presentation, for further details see the WaCT (UN-Habitat, 2021a). The levels of facility control in WaCT were developed from earlier work (Wilson, 1993) (Scheinberg et al., 2010b; Wilson et al., 2015b), while the service ladder for waste collection is a new formulation based on established practice. SDG: sustainable development goal; WaCT: Waste Wise Cities Tool.

The first is the five-level ‘ladder of service’ and ‘ladders of control’, used as part of the Waste Wise Cities Tool (WaCT) to monitor progress towards sustainable development goal (SDG) indicator 11.6.1, *the proportion of MSW (a) collected and (b) managed in controlled recovery and disposal facilities, out of total MSW generated in a city* (UN-Habitat, 2021a). To ‘count’ towards the indicators, the level of collection service and of control over the recovery or disposal facility must be at least ‘basic’ (Figure 2).

The second is the 9DBs, which provides a conceptual framework, a ‘road map’ allowing a country or city to locate their current position and to plot the way ahead. As shown in Figure 1, the early development bands, progressing through the roots and trunk of the ‘9DBs tree’, are defined largely in terms of progress on the two component parts of indicator 11.6.1, that is the percentage of people receiving a basic level of collection service, and whose waste is managed in a recovery or disposal facility meeting the basic level of control (Figure 2). The top of the trunk of the tree is DB5, the new target baseline of 95+% compliance with SDG 11.61. Further progress through branches of the tree requires first improved and then full levels of control over recovery and disposal (‘environmentally sound management (ESM)) (Figure 2(b)); and also higher levels of collection service, with segregation at source and separate collection of two, three or more fractions of MSW (Figure 2(a)), to facilitate the ‘3Rs’ and the transition from municipal solid waste management (MSWM) to WaRM. The tree shows two alternative routes, which are explained as part of the evolving story later in the paper (1980s); there is also a discussion in Part C, under priority challenges in implementation, as to whether or not the ultimate aspiration of ‘DB zero’ shown at the top of the 9DBs tree, a ‘zero waste’ circular economy, is attainable.

One point where terminology has evolved is worth noting. In the 1970s, the focus was on safe disposal, with alternative technologies for waste ‘*treatment and disposal*’. Over time, the focus shifted from ‘treatment’ towards ‘recovery’, of both materials and energy, so both recent analytical tools used here to monitor progress refer rather to ‘*recovery and disposal*’.

### Organisation of the paper

The paper has been arranged in three distinct parts: to facilitate navigation, Table 1 provides an annotated guide for the reader. The historical review is divided into two, corresponding approximately to time bands (Wilson, 2007). Part A covers the early stages in evolution of SWM from 1970 to 1990, an era which I categorise as the ‘technical fix’. Part B examines the further evolution of SWM into WaRM from 1990s to 2010s, which required a more integrated approach.

**Table 1.** A reader’s guide to navigating the remainder of this paper.

Main headings	Topics included
PART A: EARLY STAGES IN EVOLUTION OF SOLID WASTE MANAGEMENT 1970–1990 – THE ‘TECHNICAL FIX’	
The baseline around 1970	Composition of MSW
	Waste collection, treatment and disposal
	Responsibilities for waste management: MSW, industrial wastes
	Recycling: materials recycling, example of China, organic waste valorisation, informal sector recycling
	Status quo in 1970: MSWM baseline on the 9DBs, industrial and hazardous wastes, 1970 – a pivotal year
From 1970s – bringing wastes under control	New legislation: initial action driven by hazardous waste, introduction of legislation
	The ‘technical fix’: basic control standards, waste disposal planning, evaluating alternative technologies
	Institutional context: consolidation of municipal responsibilities, independent environmental regulator
	Monitoring progress on the 9DBs
From 1980s – ramping up technical standards	MSW
	Hazardous waste
	Intended and unintended consequences: NIMBY, waste crime
	Continued ramping up of technical standards since the 1980s
Slow progress in the Global South	MSWM: some failure cases; case study – Bangkok; early World Bank MSWM funding. Case study – Shanghai
	Hazardous waste: taking the first steps; case study – two Bhopal disasters
Updated 1990 baseline in Global North	
PART B: FURTHER EVOLUTION FROM 1990S TO 2010S – A MORE INTEGRATED APPROACH	
Towards a new analytical framework	What is meant by ‘integrated’? Origins of the ‘waste hierarchy’. ISWM. Strategic planning for MSWM
Governance factors	
*Local institutional coherence*	Common institutional problems. Single point of responsibility. Essential institutional functions (national and local)
*Financial sustainability*	Affordability. Costs of inaction. Revenue collection. Pricing disposal
*Provider inclusivity*	Public–private partnerships. Public or private sector operation?
*User inclusivity*	Behaviour change. Waste and gender
*National legislative and policy framework*	A new driver to reduce methane emissions from landfill. A wider range of policy instruments. Economic instruments: Legislative sticks. EPR
Global North – from waste management to waste and resource management	
*Rediscovering MSW recycling*	Recycling as a competitive ‘sink’. Policy measures to promote recycling. Increased recycling rates. Global markets for recycled materials
*Edging towards waste prevention*	Terminology: 3Rs –> 9Rs. Source reduction of industrial and hazardous waste. Food waste prevention. Prevention of MSW
Progress in the Global South	
*Progress in extending collection and controlled disposal*	1990s baseline. Recent advances in data availability. Demonstrating progress in selected cities. Billions of people still lack basic services. Fates of MSW. Waste and the SDGs
*3Rs and Informal sector recycling*	3Rs in Asia. Categories of informal recycling. Recycling rates. Changing attitudes to the informal sector. Inclusion/integration of the informal sector. The importance of separation at source. Organic waste recycling
*Investments in infrastructure*	Upgrading to controlled recovery and disposal. Accessing investment funding. Case studies: Project preparation disrupted by ‘magic solutions’. When are conditions right to invest in high-tech? Decision-makers’ guides. Selecting appropriate technologies. Inability to fund operating costs. Capacity building. How much development finance goes to SWM?
*Progress in emerging economies*	Rapid stepwise progress in China. Moving direct to EU standards in new Member States. Two case studies: is an interim step necessary?
PART C: 2020–2030 – REFLECTIONS ON PRESENT AND FUTURE PRIORITIES	
Solid waste management emerges onto the global agenda	
*A changing landscape*	Population, economic and waste growth. Changing types and composition of waste. COVID-19. Healthcare waste management
*Global action on plastics pollution*	Statistics. Ocean plastics. Mismanaged solid waste as the major source of plastics leakage to the environment. Huge increase in activity. Towards a legally binding agreement
*Waste and climate*	Historic focus on methane. Indirect carbon savings from the 3Rs. Estimated mitigation potential
*Open burning of waste*	Extent of open burning. Local and global impacts. Campaign to end open burning
*International science-policy panel on chemicals, waste and pollution*	Scope of the panel. Improving waste data. Hazardous wastes
*Exponential growth in peer-reviewed literature*	
Three key policy priorities in waste and resource management for all countries	
*Sustainable financing*	Securing investment finance for waste facilities. Readiness to absorb investment in the Global South. Basic services are often not affordable. The costs are local, the benefits global. International obligation to deliver sustainable finance. Targeting sustainable finance in the Global South
*Rethink sustainable recycling – Global North*	The MSW recycling system is broken. Don’t just focus on stimulating supply. Be prepared to pay for recycling. Target quality rather than quantity. Stimulate demand
*Rethink sustainable recycling – Global South*	Integrate recycling with formal MSWM. Build from where you are rather than follow the Global North. Move earlier to separation at source
*Need worldwide EPR with teeth*	Cover the full costs. Incentivise reduction and reuse; Take full responsibility to make recycling happen. Extend the coverage of EPR. Extend EPR upstream. Implement EPR in the Global South. Transparent monitoring of EPR
Priority challenges and directions in implementation	
*Moving towards clean cycles*	Directions in the Global North. Limits to recycling. Need for final sinks. Circular Economy in the Global South
*Extending basic waste services to all*	A continuing challenge. Build from the community upwards. A people-centred approach
*What standards for recovery and disposal?*	Upgrading disposal to ‘basic’ control standards. Is controlled recovery a realistic alternative to controlled landfill? Moving towards full control standards
Conclusions	Learning from the past . . . in order to plan for the future. Priorities moving forward. New opportunities

EPR: extended producer responsibility; EU: European Union; ISWM: integrated sustainable waste management; 3Rs: reduce, reuse, recycle; 9DBs: nine development bands; SDG: sustainable development goal; MSW: municipal solid waste; MSWM: municipal solid waste management; SWM: solid waste management.

As stated in the Introduction, it is likely that many readers will choose to begin by reading the ‘discussion’ section in Part C, which takes the form of my reflections on priorities for the decade 2020–2030. Where has WaRM got to, what are the key continuing and emerging issues and how should those be tackled? Much of my recent work has been future focused, so I present here my perspective on opportunities to shape continuing evolution over the next decade. These include influencing the negotiations for the new international legally binding instrument on plastics pollution and the science-policy panel on chemicals, waste and pollution; three key policy priorities for all countries; and priority challenges and directions in implementation.

### Use of abbreviations

It is already clear that it is difficult to write on this subject reasonably concisely without the use of abbreviations. I have tried to reduce the numbers of abbreviations used. For those which are unavoidable, or which make a particular paragraph or sub-section easier to read, I both introduce the abbreviation the first time it is used, and reintroduce it if it has not been used for some time. I hope that this will make the paper more accessible to those readers who are not waste management ‘nerds’ (and will not annoy those who are too much). A list of abbreviations is provided as an Appendix after the references.

## Part A: Early Stages in Evolution of Solid Waste Management 1970–1990 – The ‘Technical Fix’

## The baseline around 1970

### Composition of Municipal Solid Waste (MSW)

By 1970, consumerism and economic growth was beginning to see living standards rise in the Global North, which was one factor changing the nature and composition of MSW (Strasser, 1999). Another was the switch in some cities away from coal for heating. Taking the UK as an example, the 1956 *Clean Air Act* reduced coal ash in MSW – the fine fraction declined from 57% by weight before World War 2, to 28% in 1963 and 17% in 1968 (UK DoE, 1971). The same data set shows paper increasing from 14% pre-war, through 23% to 37%; metals from 4% to 8%; glass from 3% to 9%; the putrescible fraction from 14% to 18%; and plastics being first recorded separately in 1967 at 1%. Taken together, these two factors had somewhat cancelled out in terms of weight of waste generation per capita, but the time trend was beginning to increase sharply. In terms of waste density, the two trends reinforced each other, with a marked decrease from 290 kg/m³ pre-war to 200 in 1963 and 157 in 1968 (US waste was less dense, ~100 kg/m³ (Institute for Solid Wastes, 1975)); this had led to dramatic increases in waste volumes so that compaction vehicles had become standard for waste collection.

In contrast, waste composition in the Global South was dominated by 40–80% by weight putrescible organic wastes (Wilson, 1981; Cointreau, 1982), and depending on the local climate by solid fuel ash from heating and cooking; so density was high, ranging from 250 to 500 kg/m³. Moisture content at 40–80% (Cointreau, 1982) was also much higher than the 25% in the UK (Wilson, 1981).

### Waste collection, treatment and disposal

#### Waste collection

Near universal collection of mixed MSW (i.e. a basic level of service in Figure 2(a)) had already been in place for many years, at least in urban areas, in much of Europe, North America, Japan, Australia and New Zealand, the Soviet Union and China, driven by legislation focused on public health, some of which dated back to the middle of the 19th century. Collection systems also operated in at least parts of many larger cities in developing countries, some of which had been set up under former colonial administrations (Melosi, 1981; Tonge and Quincey, 1985; Wilson, 2007).

#### Waste treatment and disposal

There was not yet legislation setting out environmental controls. Land disposal and combustion were dominant for MSWM, for example, accounting, respectively, for 90%/8% in the UK (UK DoE, 1971); 72%/21% in the United States (US EPA, 1997); 40–70%/50–30% in central and northern Europe and 90–100%/10–0% in southern and eastern Europe (ERL, 1992b); and 34%/55% in Japan (Buekens, 1978).

The UK had developed the principles of ‘controlled tipping’ as early as the 1920s (Dawes, 1929;Thomson, 1932; Owen & Jones, 1934) which were reiterated again in 1967 (Bevan, 1967). Waste was to be deposited in thin layers and covered within 24 hours, with no deposit into standing water. The focus was on preventing or reducing nuisance due to flies, vermin, odour, windblown litter and fires; the principles extended to 13 bullet points and just 320 words in total (UK DoE, 1971). The United States adapted the UK principles into what they called ‘sanitary landfilling’. In terms of the landfill control ladder in Figure 2(b), sites operated under these recommendations would have met the first step of ‘limited’ control, but it is arguable as to whether they would now be considered as reaching the ‘basic’ control level required to count as controlled disposal. In the UK, an official Working Party on Refuse Disposal was set up in 1967, and their report, known after the Chairman as the ‘Sumner report’ (UK DoE, 1971), provides a valuable baseline based on 1966/1967 data from a survey of English local authorities. Their data show that, of the total wastes disposed to land, 70% met controlled tipping recommendations, while a further 29% were listed as ‘semi-controlled’. In his history of the evolution of MSWM in the United States, Louis reports that sanitary landfilling had become ‘dominant’ by the 1960s, but also that the first nationwide survey in 1968 showed a system dominated by ‘municipal dumps’ (Louis, 2004).

**Figure 3.**
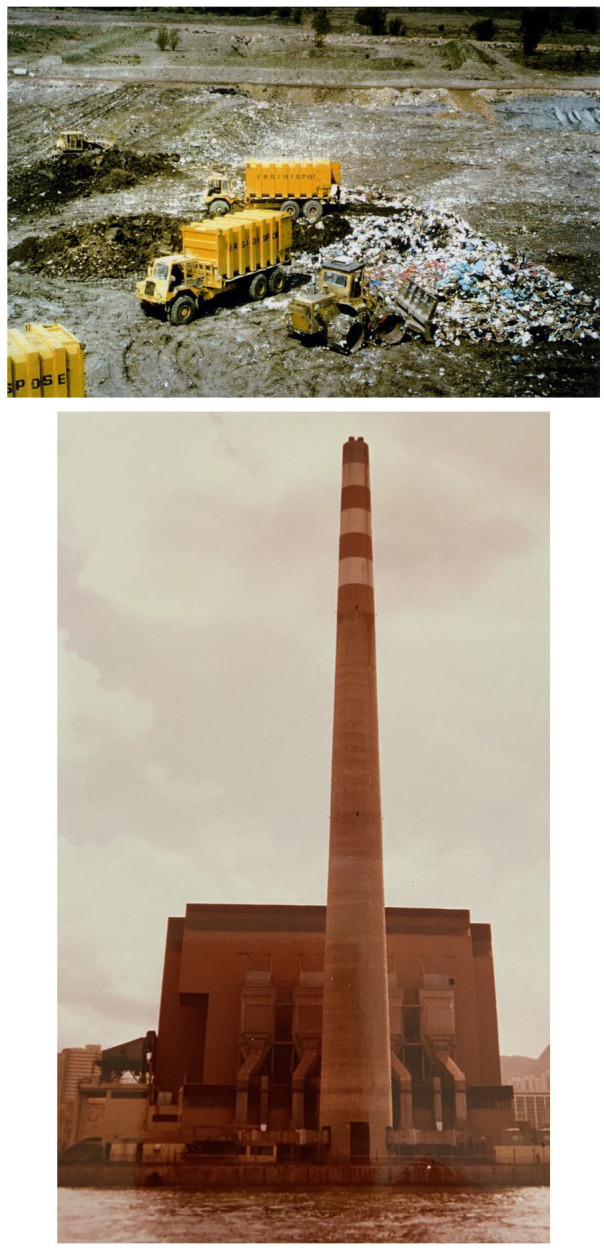
Example 1970s best practice controlled treatment and disposal facilities for municipal solid waste. Top: 1970s UK best practice landfill, illustrating operational control by the ‘3Cs’. In this example, waste was transported in containers by rail and hauled to the working face located in one cell of the site (‘Confine’). The waste is placed by a steel-wheel compactor (‘Compact’) and covered at the end of each working day (‘Cover’). Photo: David Campbell. Bottom: 1970s incinerator at Kwai Chung, Hong Kong. Hong Kong commissioned two modern moving grate incinerators designed to ensure complete combustion, at Kennedy Town in 1967 and Lai Chi Kok in 1969; emissions were controlled solely by dispersal through a 61-m chimney. Kwai Chung was built to the then latest standards between 1973 and 1978, using electrostatic precipitators for dust removal and a 150-m chimney. There was no energy recovery. The first two incinerators were decommissioned in 1991–1993 and Kwai Chung in 1997. Photo: David C. Wilson, 1983.

Most facilities for combustion were relatively old, with little or no emissions control. This was beginning to change: a new generation of modern moving-grate MSW incinerators, designed to ensure complete combustion, had begun to emerge from 1962 (Kleis and Dalager, 2004); while none was operational in the UK at the time of the 1966/1967 survey, five had been commissioned by 1970 (UK DoE, 1971), with a further 20 by 1975 (Wilson, 1981) (see also Figure 3 later). The primary purpose of such incinerators was waste treatment as a means of disposal; however, energy was recovered at some facilities as heat and/or electricity, particularly in northern European countries where centralised district heating is provided by municipal utilities in the winter.

### Responsibilities for waste management

#### Municipal solid waste management

Responsibility for MSWM was assigned at a very local level in most countries, with, for example, 1174 local authorities in England (UK DoE, 1971); and 36,453 *Communes* in France (ERL, 1992b) individually responsible. One consequence was that the professional capacity to manage wastes properly was often scarce (e.g. Louis, 2004). Another was that accurate data were often not collected routinely; for example, in the UK, only 16% of collected waste was weighed, with just 5% of authorities outside London weighing more than 50% of the waste collected. Where waste was weighed, the average reported waste generation per capita was 0.79 kg per capita per day, which compared to the overall average of 0.91, demonstrating a systematic over-estimation by 15% (UK DoE, 1971). In many countries, services were also delivered by the municipality or a municipal controlled company; in the United States in particular, some cities delegated to private contractors (Louis, 2004).

#### Industrial wastes

In 1970, extraction and mining of raw materials was already on a global scale, but manufacturing products to meet consumer demand was still predominantly in the Global North. Responsibility for managing industrial wastes lay with the industry generating the waste; in most countries, two consequences were that no data were (publicly) available; and, in the absence of regulatory controls, the predominant fate was uncontrolled land disposal, either in dedicated onsite facilities, in commercial industrial waste landfills or mixed with MSW at municipal landfills. Early controls over air pollution and effluent discharges were already adding to the quantities of both solid and liquid hazardous wastes requiring management, which were beginning to be recognised as a priority.

### Recycling

#### Materials recycling

Recycling rates reported from formal MSWM were low, for example, 6% in the United States (US EPA, 1997), 2% in the UK (UK DoE, 1971) and 1–10% in other EC Member States (ERL, 1992b). However, this is only part of the picture. A private and partly informal recycling sector operated in parallel, collecting post-consumer materials for sale to the secondary materials (recycling) industry, which had developed since the 19th century alongside the virgin materials industry as an integral part of the industrial supply chain (Scheinberg, 2003). However, industrial recyclers prefer to deal with larger volume, cleaner sources of materials with a high value, including their ‘own scrap’ or ‘home scrap’ from within a production plant, which arguably never becomes ‘waste’; ‘new scrap’, such as trimmings and cuttings of industrial or artisanal processes, or spoilage, or errors in production (Henstock, 1996); or ‘industrial salvage’, such as products that have arrived late or were over ordered and are written off by the owner. Such clean recycling has always dominated reported national recycling rates.

#### Example of China

Recycling was a key element of the centrally controlled industrial systems in the Soviet Union, Eastern Europe and China, providing a vital part of the supply chain. In China, a network of state-run Material Recovery Companies operated in major cities, in parallel to the municipal Sanitation Bureaus – when I visited Shanghai in 1985, each organisation employed 30,000+ people. High rates of recycling covered a huge range of products – including, for example, several thousand tonnes per year of human hair in both Beijing and Shanghai, used as an organic chemical feedstock. The system extended to household wastes: people separated anything saleable at home, and sold it to itinerant buyers or direct to redemption centres (of which there were e.g. 400 in Beijing) (Furedy, 1990, 1993). The resulting residual MSW had a high organic content, with high ash content particularly in Northern China in the winter. One management method has been described as ‘garbage farming’ – waste was taken to local transfer points and transported into the countryside, with 2282 deposit/composting points around Beijing (Guo et al., 2005), from which local farmers collected material for use after further composting or co-composting with animal and/or nightsoil (human excrement and urine), followed by maturation and possibly screening; a major example was in Shanghai (Furedy, 1989).

#### Organic waste valorisation

**Figure 4.**
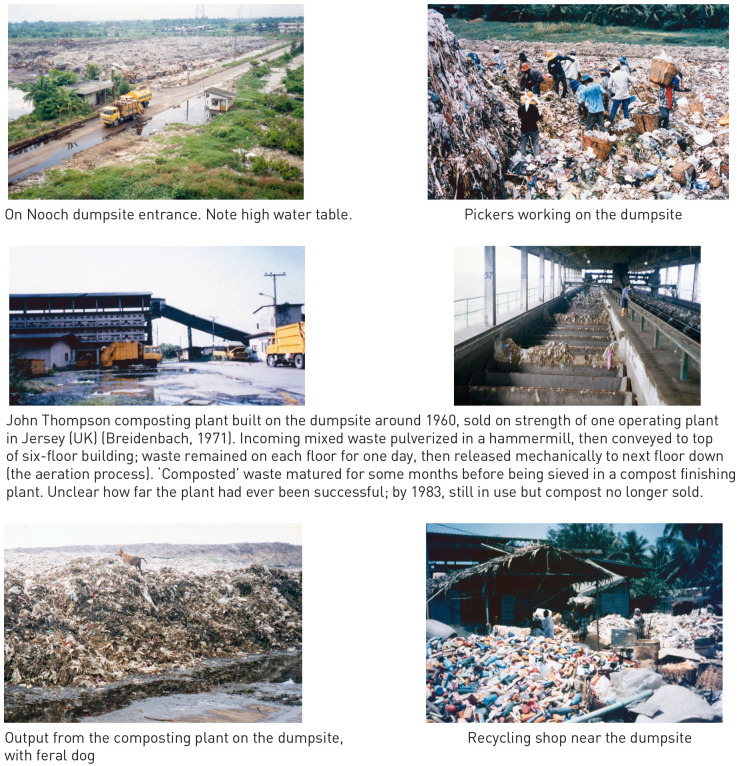
Municipal solid waste management in Bangkok in the 1980s. Most photos taken on my 1983 visit, others in 1987. © David C Wilson.

It was normal in pre-industrial societies. Any left-over food was fed to animals and ‘clean’, source-separated organic wastes (of human, animal and vegetable origin) were recycled back to the soil via composting (Scheinberg et al., 2010b). By 1970, such practices were still common, both at the household level and in more rural areas; organised collection of food wastes from restaurants and markets for use as pig feed was also relatively widespread, particularly in centrally controlled economies. Given the very high organic (and ash) content of MSW in developing countries, composting was an obvious solution as per the Chinese example. Another example of ‘garbage farming’ was in East Calcutta, where the municipal Corporation leased out 800 hectare of plots of mature waste on its 100-year-old dumpsite for intensive farming, which produced an average of 150–300 tonnes per day of vegetables and generated employment for 20,000 people (Furedy and Ghosh, 1984; Furedy, 1989). India was also home in 1970 to around 2500 small co-composting plants for MSW and nightsoil (human faeces and urine), using the Indore/Bangalore process where the wastes were layered in trenches and turned regularly over 4–6 months. Composting of organic wastes for use on farmland had been promoted by the Indian Government since 1944, with annual production at 3.3 million tonnes in 1959 (Breidenbach, 1971). Elsewhere in the world, some 30 different processes for mechanically assisted and accelerated composting of MSW had been developed, with 100 plants reported in the 1960s in 30 countries (for an example, see Figure 4 later). The amount of MSW recovered in this way was generally less than 1% on a national basis, a notable exception being the Netherlands at 17% (Breidenbach, 1971).

#### Informal sector recycling

Recycling predates formal MSWM; if a market exists for materials thrown away by the affluent, then people from more economically marginalised groups will take the opportunity to earn a livelihood (Medina and Dows, 2000). Such informal sector recycling was well documented in 19th-century London (Mayhew, 1862) and Paris (Paulian, 1896) but had largely died out by 1970 in the Global North. However, long-established ‘customary’ systems of informal recycling were still commonplace in the Global South, operating in parallel to and often ‘invisible’ to the formal MSWM sector (Wilson et al., 2006; Medina, 2007). Relatively, little had been written on informal recycling in 1970, or indeed during the 1970s and 1980s (e.g. (Meyer, 1987)), so this important subject is returned to in Part B, under ‘Progress in the Global South’.

### Status quo in 1970

#### 1970 baseline for MSWM on the 9DBs

Progress on MSWM since 1970 will be evaluated using the 9DBs (Figure 1). So where did the World stand in 1970 in terms of the 9DBs? On collection, the Global North had reached at least 80–95% coverage (DB4a), with the Global South mainly at DB1 or DB2 (collection coverage 0–60%). However, in terms of management in a controlled facility, even if the then current recommendations on ‘controlled tipping’ or ‘sanitary landfilling’ are accepted as meeting the ‘basic’ control level, then just a few countries had likely reached DB3 (controlled recovery and disposal 20–50%), with most still at DB2 or DB1. MSW recycling rates were generally low in the Global North (0–5%), variable in developing countries depending on the local customary role of informal recyclers (5–45%), and high in the centrally controlled economies (around 50%).

How to classify China in 1970? If the ‘garbage farming’ method really did work as intended, covered the whole country, and could be categorised as meeting a ‘basic’ standard of control, then arguably China could perhaps have achieved 95+% levels, at least in urban areas, for both collection coverage and controlled recovery and disposal (as measured now by SDG indicator 11.6.1), thus reaching DB5. Indeed, if the system of resource and waste management really did work like this, then arguably it had some of the characteristics of a circular economy (DB Zero)? Similar, heavily caveated, speculations have been made about other historical WaRM systems, such as the Aztecs in Mexico City around 1500 (Medina, 2014) or early 19th-century London (Velis et al., 2009).

#### Industrial and hazardous wastes

The 1970 status quo for industrial and hazardous wastes, even in the Global North, was that most wastes were self-managed by or on behalf of the waste generator. Those wastes that could not easily and profitably be recycled were mainly disposed of at essentially uncontrolled land disposal sites.

#### 1970 as a pivotal year for waste management

The ‘environmental movement’ of the 1960s meant that legislation to control environmental pollution, waste management in general and hazardous wastes in particular was under discussion in many developed countries, with official reports such as the Sumner report (UK DoE, 1971), and an earlier report on toxic wastes (UK MHLG, 1970), being commissioned to provide a firm evidence base. It is only partly co-incidence that 1970 also saw the founding of the International Solid Waste Association (ISWA); and the UK national member of ISWA, the professional body for those working in the sector, changed its name in 1971 from the *Institute of Public Cleansing* to the *Institute of Wastes Management* (later the Chartered Institution of Wastes Management (CIWM)).

## From 1970s – Bringing wastes under control

### New legislation

#### Initial action in UK driven by hazardous wastes

I went up to Oxford University in 1970 to read Chemistry. When I was working on my fourth-year thesis on theoretical chemistry, I decided that I wanted to find a job doing ‘something mathematical to save the environment’. I ended up in the Hazardous Wastes Service, which had been set up at the Harwell Laboratory in 1972 to transfer skills from radioactive to hazardous chemicals and wastes, in response to a hazardous waste crisis: the discovery of abandoned drums of solid cyanide wastes in an area where children played, near Nuneaton in the industrial West Midlands, caused public outrage and resulted in the 1972 *Deposit of Poisonous Wastes Act* being rushed through Parliament in record time (Wilson, 1982a).

#### Introduction of legislation

The advert I replied to was to research ‘*A Systems Approach to Municipal Solid Waste Management (MSWM) Planning*’, which became my doctorate thesis. The topic was a direct response to the 1974 *Control of Pollution Act* which was the first UK legislation controlling waste disposal to land. The scope of the *Act* included municipal, commercial and industrial wastes, including hazardous wastes but specifically excluding most mining, quarrying and agricultural wastes. Similar waste legislation was enacted around the same time in Denmark, France, the Federal Republic of Germany and the Netherlands, and formed the basis of the 1975 European Community (EC) *Directive on Waste (75/442/EEC)*, which, in turn, led to legislation in other EC Member States; also in Japan in 1970 (Sigita, 1979) and in the United States at both State and Federal levels (the *Resource Conservation and Recovery Act* of 1976; Louis, 2004).

Much of this new generation of environmental and waste legislation provided a framework, which then needed to be implemented through further regulations and either statutory or advisory guidance. Key components included the licensing (permitting) of disposal and treatment facilities to meet defined standards; the preparation of waste disposal (or wider waste management) strategies or plans; and institutional responsibilities for waste collection and disposal, and for the inspection of licensed facilities and enforcement of license conditions (Wilson, 1981). In the UK, implementation of the *Control of Pollution (Special Waste) Regulations, 1980* under the 1974 *Control of Pollution Act* repealed the earlier *Deposit of Poisonous Wastes Act*; as with subtitle C in the US *Resource Conservation and Recovery Act*, the basic provisions for hazardous wastes were similar to other wastes, but with additional requirements to ensure that the waste arrived at its designated destination (Wilson, 1979a).

### The ‘technical fix’

#### Basic control standards

The primary focus of the legislation was technical, to raise the level of control of treatment and disposal facilities to the ‘basic’ level as shown in Figure 2(b), to become formally ‘controlled’. For landfill of MSW, the initial focus was on operational control, often summarised as the ‘3Cs’, confine (within a ‘cell’), compact, cover (Figure 3). For example, the UK best practice guidance on landfilling wastes built on the earlier recommendations on controlled tipping (see 1970 baseline), with model conditions included in guidance on licensing of waste disposal sites (UK DoE, 1976b). For incineration of MSW, the first phase of control combined moving grate (mass burn) technology first introduced in the 1960s to ensure controlled combustion (see 1970 baseline) with electrostatic precipitators to remove dust. Figure 3 illustrates two typical best practice 1970s facilities.

For hazardous wastes, the early 1970s saw the opening of commercial facilities in parts of the Global North for the thermal destruction of toxic and persistent organic wastes by high temperature incineration; for chemical treatment (e.g. neutralisation of acids and alkalis, oxidation of cyanide, reduction of hexavalent chromium); for chemical or physical fixation (solidification) of heavy metal salts; and for ‘safe’ landfill (Wilson, 1979a).

#### Waste disposal planning

The UK guidance document on waste disposal planning stated the objective as ‘the disposal of waste at the least possible cost to the community with due regard to the safeguarding of the environment and the use of waste as a resource’ (UK DoE, 1976a). Again, the focus was on the technologies – I paraphrase the original objectives of my doctorate research as: what type of facilities to build? where? when? how many? how big? Stated in this way, mathematical modelling was an obvious approach to evaluate alternative plans; indeed, my initial literature review highlighted that waste planning had features that made it very ‘interesting’ to academics. For example, in the conventional ‘warehouse location problem’ for optimising the delivery of goods from their initial sources to final destinations (Baumol and Wolfe, 1958), the quantities entering each warehouse are the same as those leaving. For MSWM, while this is often true for transfer stations which transfer the waste from smaller to larger vehicles to reduce transport costs, trans-shipment through other treatment facilities changes both the nature and the quantities of the waste leaving the site. My office was in the control tower of the Harwell wartime airfield, which housed the Operations Research Group.

Unfortunately, this had resulted in a focus more on the elegance of the model rather than on its practical application to assist the waste planner in understanding their system (Wilson, 1977a, 1977b). I did return to the challenge of developing a more practical model to assist the Hong Kong Government in assembling and evaluating alternative waste disposal plans (Wilson et al., 1984; Hoare et al., 1985; Wilson, 1985; Rushbrook, 1987). However, with the benefit of hindsight, computer systems had not yet developed sufficiently to support the types of practical, user-friendly decision support system we aspired to – indeed, realising that aspiration is still proving elusive (Finneviden et al., 2007; Chang et al., 2011; Zurbrügg et al., 2014; Asefi et al., 2020; Campitelli and Schebek, 2020).

#### Evaluating alternative technologies

My thesis turned more towards earlier steps in the MSW planning process, in particular the comparative evaluation of alternative technologies considering both economics (Wilson, 1978, 1979c) and energy efficiency (Wilson, 1979b). The resulting textbook (Wilson, 1981; Hansen, 2003) provides a state-of-the-art review of technologies for MSWM as available in the 1970s. The technologies with a substantial commercial and operational track record were transfer, landfill, incineration and composting; emerging technologies were physical separation of wastes, refuse-derived fuel (RDF) and anaerobic digestion. Incineration was again categorised primarily as waste treatment/disposal, although it was noted that there were 200 modern incinerators with energy recovery in Western Europe and 50 elsewhere, mainly in Japan. One problem cited from the four UK energy recovery incinerators was corrosion of water walls and heat exchanger tubes. Commercial-scale demonstration facilities utilising pyrolysis, gasification, wet pulping and hydrolysis were also included, but most resulted in project failures; one 900 tonnes per day demonstration gasification plant in Baltimore, Maryland was noted as struggling; a few years later, it had already been replaced with a mass-burn incinerator with energy recovery (Feindler, 1984).

### Institutional context

#### Consolidation of municipal responsibilities

It was recognised that many small municipal authorities would have difficulty achieving controlled disposal and treatment, due to a lack of scale and of technical and financial capacity. In the UK, the Greater London Council had been formed in 1965 and was assigned responsibility for waste disposal, while collection remained with the 32 London Boroughs and the City of London. The 1974 *Control of Pollution Act* coincided with major reform of the remaining local authorities along similar lines, with the previous 1174 authorities in England being replaced by 45 counties, which became Waste Disposal Authorities, and 332 districts which became Waste Collection Authorities. Similar consolidation occurred in other countries, with, for example, responsibility for disposal in France being transferred from the 36,453 *Communes* to just 100 *Départments (ERL, 1992b)*.

#### Independent environmental regulator

The other important institutional element of the new legislation was that of the environmental regulator, responsible inter alia for issuing waste management site licenses, inspecting operations and enforcing license conditions; for tracking shipments of hazardous wastes; and also for a ‘level playing field’, ensuring that the legitimate waste industry is not undercut by waste criminals. In England, this role was initially assigned to new Waste Regulation Authorities, who sat alongside the Waste Disposal Authorities at County level; that changed in 1995 with the establishment of the Environment Agency, bringing together the National Rivers Authority, Her Majesty’s Inspectorate of Pollution and all the Waste Regulation Authorities to form a single, independent and ‘arms-length’ regulator.

### Monitoring progress on the 9DBs

The main focus in bringing wastes under control in the Global North was to phase out open dumping and other forms of uncontrolled disposal for both hazardous wastes and MSW. In parallel, efforts were made to extend MSW collection to all, particularly in smaller urban communities and in rural areas. The rate of progress towards DB5, the target baseline of near universal (95+%) collection coverage and controlled recovery (then still termed ‘treatment’) and disposal for MSW, varied widely between countries.

## From 1980s – Ramping up technical standards

### Municipal solid waste

While most countries were still at the stage of bringing MSW under control, some of the highest income countries quickly ramped up technical standards, moving from basic controlled treatment (recovery) and disposal (DB5) in a series of steps through improved control towards full control or ESM. The focus for landfill was on leachate collection and treatment, and on gas collection with either flaring or energy recovery. As an example, landfill guidance in the UK expanded from just 320 words (DoE, 1971 – Sumner report), to one volume of 200 pages (UK DoE, 1986), to six volumes and 500+ pages by 1996 (UK DoE, 1991; UK DoE, 1994–96). For incineration, gradually increasing standards for emissions to air required additional stages for gas cleaning, initially focusing on acid gas removal (Damgaard et al., 2010).

The 9DBs distinguish for MSW between two alternative paths to progress beyond DB5 through the ‘leafy branches’ of the ‘9DBs tree’ (Figure 1). The UK and the United States chose to rely on market mechanisms operating within a highly regulated framework, and were content to use mainly landfill disposal (DB6), backed up by substantial research programmes; while much of central, western and northern Europe and Japan moved strongly towards high recovery systems (DB7), for example, with combined heat and power incinerators feeding into local district heating schemes (Kleis and Dalager, 2004; Whiteman et al., 2021).

### Hazardous waste

My first job after my doctorate was at Harwell, running the (hazardous) Waste Research Unit under contract to the then UK Department of the Environment. In 1981, I was invited to be rapporteur for a working group which resulted in the first international policy guidelines and code of practice for hazardous waste (‘hazwaste’) management (WHO and UNEP, 1983). I was a founder member of ISWA’s first Working Group, on hazwaste (WGHW), which was set up at the ISWA annual business meeting in 1984. Looking at the Proceedings of the parallel ISWA Congress in 1984, it is notable that all the keynote country reports consider both MSW and hazwaste (Formaglini, 1984), whereas more recently such papers have largely become separated into ‘silos’. The WGHW published a set of 12 country reports (Forester and Skinner, 1987) alongside a comparative analysis (Wilson and Forester, 1987; Wilson, 1999b), which showed both significant commonality of the legislative approach, but also considerable diversity in implementation (Wilson and Parker, 1987). A state-of-the-art guide for decision-makers on safe hazardous waste management systems was also prepared and later updated (ISWA WGHW, 1991, 2002).

In many countries, both the quantities and range of hazwaste being treated in centralised facilities steadily increased over time, with technical standards also being ramped up; for example, high-temperature rotary kiln incinerators with long gas residence times and multi-stage gas cleaning became the norm in the 1980s. For landfill, those countries following DB7 for MSW often took action to restrict the range of hazwaste that could be accepted, for example limiting that to residues from incineration or chemical treatment, which might then require solidification prior to landfilling. These countries often had the power to direct wastes to a particular facility, sometimes run by a public or public–private hazwaste utility company (e.g. Denmark, Sweden, Netherlands). France provided a 50% subsidy of the disposal price to a company using the environmentally preferable disposal option (Forester and Skinner, 1987; Wilson and Forester, 1987).

The main DB6 countries also relied on both strong regulation and market forces for hazwaste. For example, following the Love Canal scandal, the US enacted the 1980 *Comprehensive Environmental Response, Compensation and Liability Act* (commonly referred to as ‘Superfund’), under which any company which sends waste to landfill is held legally responsible for the entire costs of any future clean-up. In 1984, the *Hazardous and Solid Waste Amendments* drastically ramped up the technical standards for landfill under the previous 1976 *Resource Conservation and Recovery Act*, and banned land disposal of most liquid wastes and selected other hazwastes. Then in 1986, the *Superfund Amendments and Reauthorization Act* completely rewrote and ramped up earlier requirements. Taken together, these ‘legislative sticks’ led to hazwaste management dominating the new, rapidly growing environmental consultancy sector in the United States (LaGrega et al., 1994).

The UK took a rather different approach; the Department of Environment commissioned the British Geological Survey, Harwell and the Water Research Centre to undertake long-term hazwaste research programmes from the early 1970s, with the aim of providing the evidence base (UK DoE, 1978; McGahan, 1986a; Rushbrook, 1989) to safely manage the controlled co-disposal of selected hazwastes in MSW landfill sites (McGahan, 1986b; UK DoE, 1996).

### Intended and unintended consequences

Rising technical standards led to a rapid increase in facility costs. One intended consequence was improved economics for waste reduction, reuse, recycling (the ‘3Rs’), which are explored further in Part B. Economies of scale meant fewer, larger sites, to which MSW was transported via transfer stations, which was intended to encourage regional cooperation between municipalities.

#### NIMBY

However, larger sites added to existing public opposition to new waste facilities, generally known as ‘not in my backyard’ (NIMBY) syndrome. This likely had its origins in the ‘sins of the past’; for example, one popular UK author described the nuisance caused by ash, dust and charred paper from 200+ (uncontrolled) refuse destructors built within towns between 1874 and 1914, and added: ‘*Thus began the long and bitter opposition to incinerators that has never ceased*’ (Girling, 2005). Siting new hazardous waste facilities in North America in the 1980s was particularly difficult due to concerns from Love Canal and other uncontrolled sites. The best-known example of successful siting at the time was in Alberta Canada, where the usual ‘top down’ planning was replaced by an innovative approach based on decentralisation of decision-making authority and full and meaningful public involvement (McQuaid-Cook and Simons, 1989; Rabe, 1992; Kuhn and Ballard, 1998).

Another unintended consequence is what I have called the ‘implementation conundrum’, felt particularly in (DB6) countries like the United States or UK who rely heavily on market forces (Wilson, 1995). When a new, higher-cost facility comes online, the older, lower standard and cheaper facilities may not close immediately, leading to a period of unfair competition; there were several examples in the United States in the 1980s where new hazardous waste landfill sites went bankrupt during this interim period (Wilson and Parker, 1987). This lack of ‘regulatory certainty’ continues to be cited in the UK as a major constraint to the development of new high-tech hazardous waste facilities (Wilson and Smith, 2005; Wilson, 2018a).

The forced closure of non-compliant landfills and incinerators, and the delay in permitting new facilities due to public opposition and NIMBY caused a ‘garbage crisis’ in the United States in the 1980s. The resulting regionalisation of MSW disposal facilities also resulted in an unintended shift from public to private sector provision of services; disposal operations were largely municipal in 1980, but had been transferred to a small number of private companies by 1990 (Louis, 2004).

#### Waste crime

The sharp rise in the costs of full control environmentally sound management (ESM) facilities also presents an opportunity for organised waste crime, both locally and globally (Baird et al., 2014). There were many high-profile scandals in the 1980s, where hazardous wastes were illegally exported and dumped in Eastern Europe and Africa (Basel Convention, n.d.); but scandals also occurred in Western Europe, as when drums containing dioxin residues from decontamination of the Seveso factory in Italy following the 1977 accident (Wilson, 1982b; Bromley et al., 1983) were found at a warehouse in France. Such incidents led in 1989 to the first multilateral environment agreement, the *Basel Convention on the Control of Transboundary Movements of Hazardous Wastes and their Disposal*, which has now been adopted by some 200 countries and other contracting parties.

### Continued ramping up of technical standards since the 1980s

Technical standards continued to be increased beyond the 1980s. In the EU, significant landmarks were provided by Directives in 1989 on *air pollution from municipal solid waste incineration plants* (89/369/EEC and 89/429/EEC, subsequently replaced by 2000/76/EC); and in 1999 on *landfill of waste* (1999/31/EC), which aimed to level up all Member States to the same high standards.

The 1989 incineration directives introduced, inter alia, a limit for dioxins near or below the then analytical detection limit (a ‘stretch’ target). All existing incinerators had until 1996 to upgrade to the new standards, or close down; typical upgrade costs were said to be around Euro40 million per plant. The results were dramatic: in the UK, dioxin was first added to the annual National Atmospheric Emissions Inventory (www.naei.org.uk) in 1990, when the total emissions were 1142 g I-TEQ/year, of which 52% was due to MSW incineration; by 1999, the total had been reduced by 70% to 345 g I-TEQ/year, of which less than 1% was due to MSW incineration (UK Defra, 2002).

Over time, incineration has evolved from a primary focus on waste treatment and disposal, to a focus on energy recovery. The *EU Waste Framework Directive (2008/98/EC)* introduced a test intended to classify any incinerator as either a disposal (‘D10’) or a recovery (‘R1’) facility. In principle, the threshold energy efficiency to qualify as R1 recovery is (for new facilities) 65% (CIWM, n.d.), which would classify waste-to-energy incinerators utilising only electricity as ‘disposal’, while most plants utilising heat or combined heat and power would be ‘recovery’. However, the guidelines are not so clear cut (European Commission, 2011; Viganò, 2018); a recent lobby group report claims the R1 threshold could be achieved at net efficiencies below 20%, and thus recommends reclassifying incineration again as a disposal process (Zero Waste Europe, 2023).

Incineration produces several types of solid wastes requiring further management. Bottom ash is widely used as an aggregate in construction, while air pollution control residues generally require significant pre-treatment prior to landfill (Christensen, 2011); some may be classified as hazardous wastes.

The impact of implementing the Landfill Directive was felt particularly in the UK; I represented CIWM on the multi-stakeholder Hazardous Waste Forum, set up to prepare for implementation of the ban on co-disposal of most hazardous wastes in MSW landfill sites from December 2003, and acted as rapporteur for their action plan (Hazardous Waste Forum, 2003). Technical standards for waste incineration and waste treatment in the EU now come under the ‘BREF’ framework, that is Best Available Technologies for pollution control, under first the *Integrated Pollution Prevention and Control Directive* (96/61/EC) and later the *Industrial Emissions Directive* (2010/75/EU) (European IPPC Bureau, n.d.).

Technical standards for the use of recycled organic wastes have also been increased in the Global North. The use of compost for food production is generally limited to compost from source-separated food or garden waste, not from mixed MSW. A major outbreak of animal disease in the UK in 2001 was attributed to contaminated food waste being fed to pigs, which resulted in a widespread ban on the feeding of kitchen or catering waste to farm animals (zu Ermgassen et al., 2018; Boumans et al., 2022).

## Slow progress in the Global South

### Municipal solid waste management

#### Some failure cases

Elsewhere in the world, populations and cities had continued to grow, but not so much had changed regarding waste management in the 1970s and 1980s; by 1990, collection coverage remained relatively low, while uncontrolled disposal was still the norm. An obvious approach was technology transfer; however, early attempts to export technologies designed for American, European or Japanese wastes, regulatory systems, cultures and income levels, often resulted in failure. For example, there were many examples where donated, reconditioned, hydraulic compaction waste collection vehicles designed for use in the Global North either could not cope with the wet, dense waste; or were too heavy for local roads; or could not access narrow streets; or could not be maintained locally (Arlosoroff, 1991; Coffey, 1988; Coffey and Coad, 2010). Several incinerators designed for European waste were also installed in West African cities, which simply could not burn the local waste.

#### Case study: Bangkok

My first field trip in the Global South was to Bangkok, at the invitation of the city technical department. I visited a dumpsite, and the composting plant built some 20 years earlier using European technology which by 1983 was only semi-functional (Figure 4). The Technical Director asked me to comment on a draft Masterplan report prepared by one bilateral donor, which I read in his office; my memory is that it found the waste to be too wet to burn, but still recommended buying an incinerator from one of their manufacturers on the assumption that increases in living standards over the construction period would raise the calorific value. I spent time in Bangkok 4 years later, when a team funded by a rival country was preparing a new Masterplan, using a similar, technical focused methodology; their survey work showed that the paper and plastics content of the waste as generated had indeed increased, as had the quantities separated by the active informal recyclers collecting the waste door-to-door, on the streets and at the dumpsites (see Figure 9 later) – so the calorific value of the residual waste was unchanged.

#### Early World Bank funding for MSWM

The World Bank had started funding MSWM projects in 1974. Their first project guide on urban solid wastes in developing countries was the early ‘go-to’ reference on the subject (Cointreau, 1982). A review of their 71 projects with a MSWM component between 1974 and 1988 showed a total investment of $532 million in MSWM, less than 8% of total project costs; 75% of the projects focused on urban development and 13% on water and sanitation. Just three projects, in Nigeria, Singapore and Mexico, focused more than 50% on MSWM and accounted for $209 million (40% of the total MSWM spend): the average cost of the remaining projects was $4.7 million (Bartone et al., 1990). ‘*The majority of MSWM investments encountered implementation problems and delays, due to poorly defined institutional structure and responsibilities, poor cost recovery, insufficient technical and managerial expertise and other problems*’ (Arlosoroff, 1991).

The World Bank also led a UN Development Programme (UNDP)-funded 10-year project in the 1980s on integrating waste management with resource recovery and recycling. The first project report reviewed the status of recycling from MSWM around the world, with an annotated bibliography of 200 publications (Cointreau et al., 1984).

#### Case study: Shanghai

I moved in 1985 from Harwell to the environmental consultancy ERL (later ERM). Shortly afterwards, I was invited to Shanghai to run a training course on MSWM for the municipal Sanitation Bureau. I found a city in a state of flux; the hotel I stayed in was one of the few high-rise buildings in the city, which was already remarkable even a decade later. The former system of garbage farming was beginning to unravel, because farmers had access to subsidised chemical fertilisers and the nature of the waste was changing, with, for example, plastics increasing, so the old network of small transfer points from which farmers had collected the waste for composting were becoming uncontrolled disposal sites. This experience was not unique; every mixed waste composting plant I visited around the world used their compost on a flower bed by the office building, where the marketing effect was spoilt by the evident contamination, particularly by film plastics. Separate collection for materials recycling was also beginning to decline as the country moved towards a more market-based system (Furedy, 1990). So by 1990, it is likely that China had reverted from ‘near DB5’ (95+%) to DB4a (80–95%) on collection coverage and DB3/DB2 (0–50%) on controlled recovery and disposal.

### Hazardous waste

The Basel Convention sought to limit and control the export of hazardous waste (hazwaste) from the Global North to South, but also focused attention on the general lack of progress in the Global South in terms of managing their own hazwaste, which, in turn, made it difficult to improve sanitation, MSWM or wastewater treatment.

#### Taking the first steps

I was heavily involved in international efforts to help developing countries take their first steps in hazwaste management, including a World Bank Technical Manual (Batstone et al., 1989); a workshop for the ISWA WGHW which led to a Special Issue of WM&R (Wilson and Balkau, 1990); a technical assistance programme to help countries implement a ban on the dumping of industrial wastes at sea (Ross, 1995); and a Training Resource Pack developed jointly by ISWA, UNEP and the Basel Convention (Wilson et al., 2002). One strand of this work was on interim measures or transitional technologies, to allow a basic standard of control to be achieved as an interim step on the path towards ESM (c.f. Figure 2(b)) – ‘*it is better to do something than to investigate for too long*’ (Wilson and Balkau, 1990). Example case studies included co-disposal and encapsulation in South Africa (de Bruin, 1990) and chemical treatment in Bangkok (Lohwongwatana et al., 1990). Training materials on transitional technologies including co-disposal were included in Wilson et al. (2002).

#### Case study: the two Bhopal disasters

The chasm between the Global North and Global South on hazwaste is illustrated by one case study. The United States first introduced Superfund legislation in 1980 and used that to clean up old uncontrolled hazwaste sites. The World’s worst industrial disaster occurred at a joint venture US-Indian chemical company’s pesticide plant in Bhopal on 2 December 1984, when a gas leak killed 8–10,000 people that night, and more than 25,000 by 1994, with at least 150,000 suffering long-term effects (Varma and Varma, 2005; Wikipedia, 2020). Partly in response, the US *Superfund Amendments and Reauthorization Act* 1986 introduced *Community Right-to-Know* provisions which require industries in the United States to plan for emergencies and inform the public of chemicals being used (LaGrega et al., 1994).

However, a second Bhopal disaster actually predates the first; hazwastes were chemically treated on site, with sludges disposed of initially in unlined pits, and later in solar evaporation ponds which suffered cracks in the liners. This resulted in extensive contamination of soil and groundwater; despite some remediation work in the years following the accident, the site and groundwater remain highly contaminated and causing ongoing chronic illness (Ansell and Tinsley, 2011). So, Bhopal partly led to a major upgrade of the US Superfund, but despite being majority owned by a US company, remains as arguably one of the worst uncontrolled and unremediated hazwaste sites in the world.

## Updated 1990 baseline in the Global North

### Municipal solid waste management

I undertook an assessment of the state of MSWM across the then 12 Member States of the EC, which compiled comparative data up to 1990 (ERL, 1992b, 1992c). This showed that, by 1980, some countries had already reached development band DB5, including Denmark, the Federal Republic of Germany, the Netherlands and the UK. France introduced their legislation in 1975, and by 1980 had increased collection coverage from 80% to 95% of the population, reaching 98% by 1985 and 99.5% by 1989; and controlled treatment (recovery) and disposal from 30% in 1975, to 56% by 1980, 91% by 1985 and 94% by 1989 (ERL, 1992b); this corresponds to DB3 in 1975, DB4 in 1980 and (almost) DB5 by 1989. This relatively slow progress in completing the closure of open dumps was due to the large numbers of very small sites (less than 10 tonnes per day); by 1989, 648 of these had been licensed, but a further 7000 remained unlicensed. Progress was still slower in the more rural southern EC Member States: by 1990, Greece had collection coverage 100% in urban areas and 69% rural (85% average); while Portugal increased coverage from 64% in 1980 to 75% in 1985. Rates of controlled treatment (recovery) and disposal (1989/90) were 38% Portugal (DB3), 42% Italy (DB4a/DB3), 74% Spain (DB4) and unquantified (only 1500 out of 5000 sites were controlled) in Greece (ERL, 1992b).

Other countries in the Global North had also made significant progress by 1990. Most had reached DB5, achieving near universal collection and controlled treatment (recovery) and disposal, while some had moved beyond that towards improved or full control standards: the UK and the United States moving along one pathway still relying heavily on landfill (DB6), with central and northern European countries and Japan focusing more heavily on incineration with energy recovery (DB7). The approach adopted was focused primarily on technology, with due attention to consolidating institutional responsibility into larger sub-regional or regional units (US EPA, 1989, 1995).

MSW collection was still predominantly of mixed wastes, that is, the basic level of service in Figure 2(a); one exception was in Japan, where in some cities householders were required to separate waste into two fractions, combustible (for incineration) and non-combustible (Tanaka, 1988). ‘Bring’ collection systems, where householders could separate, for example, paper and glass and bring them to communal containers, were beginning to be commonplace. MSW recycling rates generally remained at relatively low levels: early movers towards ‘rediscovering recycling’ included the United States and Canada (Scheinberg, 2011), and Germany and the Netherlands (ERL, 1992b) (see Part B below).

### Hazardous waste

The Global North had made significant progress in bringing most industrial and commercial hazardous wastes under control and were moving towards ESM. Indeed, many countries had already instituted systems to separate small quantities of household hazardous waste from MSW (ERL, 1992c; Scharff and Vogel, 1994).

## Part B: Further Evolution from 1990s–2010s – a more Integrated Approach

## Towards a new analytical framework

Up to the 1990s, the general approach to waste management issues was driven by legislation and technologies; how to meet the legislative standards of environmental control at least cost. Attention to institutional issues had focused primarily on making municipalities large enough to support the technical capacity and achieve the economies of scale to implement the ‘technical fix’.

That focus gradually shifted in the 1990s towards a more, holistic, interdisciplinary, systems thinking, ‘integrated’ approach. This shift was perhaps most explicit in the Global South, where a consensus was beginning to emerge that the major constraints to progress were institutional and financial rather than technical. However, it also impacted the Global North; I clearly remember hearing a Canadian speaker at a conference in the late 1990s stating that SWM is 10% technical and 90% what he termed ‘political’ (unfortunately, I can’t remember the who or the where . . .). That said, politicians do like ‘modern’ technology, and efforts to promote a more integrated approach in the Global South have always been in competition with an army of technology salespeople.

### What is meant by ‘integrated’?

**Figure 5.**
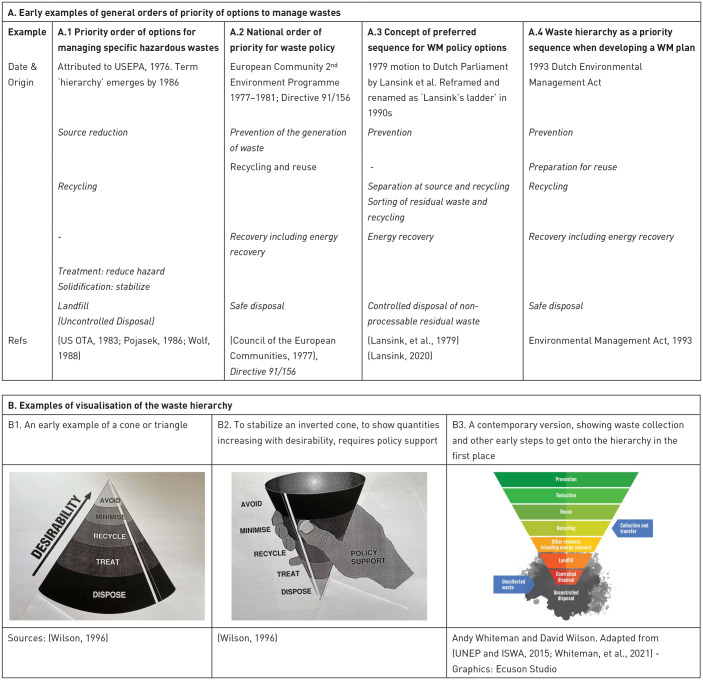
Evolution of the concept and presentation of the waste hierarchy. Part A shows examples of early representations as a ‘bullet list’ of priorities. Part B shows two early and one contemporary examples of diagrammatic representation. The terminology for, number of, and differentiation between, levels within the hierarchy varies widely.

The term ‘integrated’ has been used in many different contexts in relation to waste management: our review in 2012 identified at least 14 different thematic uses (Wilson et al., 2013). Many of these uses are largely technical and in the context of the Global North. One example is the early use of the ‘waste hierarchy’ in the United States, as a practical tool for selecting the preferable option for management of specific hazardous waste streams (Figure 5(A.1)). An integrated approach makes all options available; ‘while it is preferable to remain at the top of the hierarchy, technical and economic constraints keep forcing the selection of lower options’ (Pojasek, 1986). For MSW, the concept was not used in the first edition of US EPA’s decision-makers’ guide (US EPA, 1976), but the waste hierarchy is central to the second edition (US EPA, 1989, 1995).

### Origins of the ‘waste hierarchy’

The term ‘waste hierarchy’ has become ubiquitous since the 1990s as more of a conceptual hierarchical sequence of preferred options in waste management. Figure 5(A) shows the evolution of the concept, from its origins in the 1970s as a 4- or 5-point bullet list (Figures 5(A.2) and (A.3)), to its adoption (and one of the earliest explicit uses of the term ‘waste hierarchy’) in the 1993 Dutch *Environmental Management Act* (Figure 5(A.4)).

Integration of WM also refers to integrated policy, strategy and plans that encompass all the levels of the waste hierarchy and facilitate the general objective to ‘move WM up the hierarchy’. When I wrote on this topic in 1996, I first drew the hierarchy as a triangle or cone sitting on its base (Figure 5(B.1)). I observed that the area or volume given to each option was inversely proportional to its desirability, so proposed instead to ‘flip’ the cone to stand on its point, illustrating the desired outcome that most wastes are prevented rather than disposed of. Standing a cone on the tip is unstable, so I added ‘policy support’ to Figure 5(B.2).

The exact terminology used for each ‘level’ of the hierarchy and the number of levels vary widely, but generally waste prevention sits at the ‘top’ followed by reuse, recycling, ‘treatment’ or later ‘recovery’ (including energy recovery), and disposal of residual waste. Most versions assume that the waste is already collected (the basic service level in Figure 2(a)), and that disposal meets full control or environmentally sound management (ESM) standards (Figure 2(b)). Thus, my personal preference is for Figure 5(B.3) or similar, which explicitly shows the preliminary steps of collecting the waste and improving control standards, by moving from uncontrolled disposal to controlled disposal (to meet SDG 11.6.1) and then to (ESM) landfill, to get onto the conventional hierarchy in the first place.

When we reviewed different uses of ‘integrated’ in relation to WM, we found three dictionary meanings (Wilson et al., 2013):

i. ‘Combined or composite, made up of parts that work well together’;
ii. ‘Combining separate things, bringing together processes that are normally separate’ and
iii. ‘Open to all people, as in integration by, for example, race, ethnicity, religion, gender or social class’.

The first two correspond to the examples above, while (iii) suggests broadening the concept to include explicitly all the stakeholder groups involved in WM, which is one of the objectives of the new analytical framework introduced next.

### Integrated sustainable waste management

**Figure 6.**
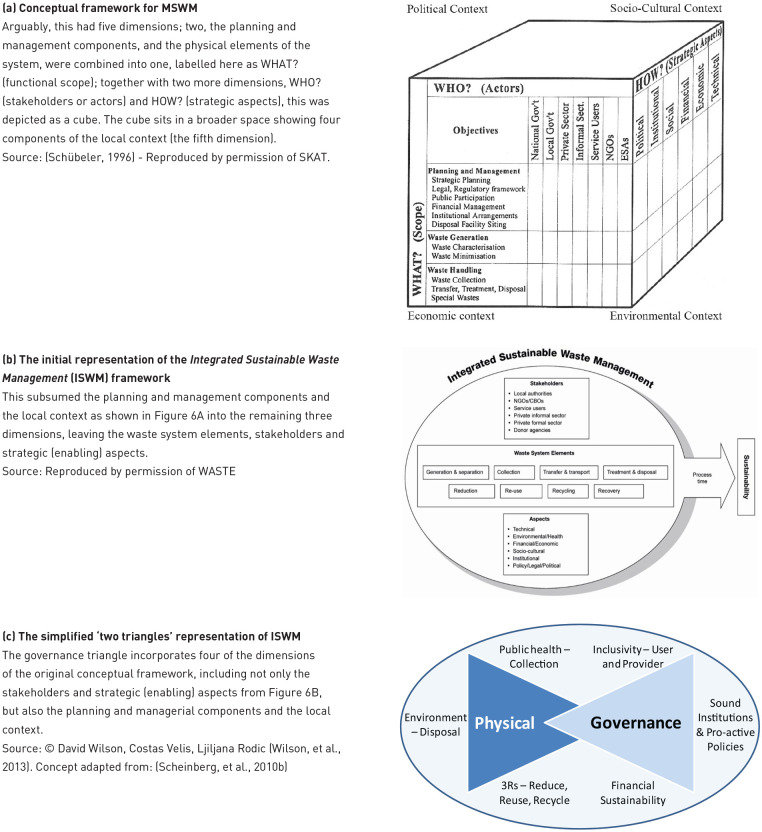
Evolution of the Integrated Sustainable Waste Management (ISWM) framework. The original framework (a) arguably had five dimensions, and was gradually simplified to, (b) three dimensions and then, (c) two dimensions, to make easier both its visual representation and its use as an analytical tool.

The integrated sustainable waste management (ISWM) analytical framework was first articulated in the context of developing countries, where the constraints to extending collection coverage and controlled disposal had been identified as institutional and financial rather than technical. The Urban Management Programme (UMP) was a joint activity of UNDP, UN-Habitat and the World Bank; and the Swiss Development Cooperation funded a UMP Collaborative Programme on MSWM in low-income countries from 1995 to 2000. Their first stakeholder workshop at Ittingen Switzerland in 1995 agreed a 5-year programme and established a Collaborative Working Group (CWG). The first output was a ‘conceptual framework for MSWM in low-income countries’ (Schübeler, 1996); for visual representation, this was simplified into a cube sitting against a background (Figure 6(a)).

One of the thinkers behind the conceptual framework was Arnold van de Klundert of Dutch institute-type non-governmental organisation (NGO) WASTE. The Dutch government funded a 6-year Urban Waste Expertise Programme led by WASTE from 1995, which refined the conceptual framework into an analytical tool (Figure 6(b)), now termed *Integrated Sustainable Waste Management* (ISWM) (Van de Klundert and Anschütz, 2001; Anschütz et al., 2004).

The successor to the CWG formed the backbone of the 35-strong international team which I co-led to prepare a seminal report for UN-Habitat on ‘*Solid Waste Management in the World’s Cities’* (Scheinberg et al., 2010b). We wanted a practical tool to compare on a consistent basis the performance of the combined (formal) MSWM and (often informal) recycling systems (collectively referred to as the waste and resource management (WaRM) system) in cities, whatever the income level of the country. To achieve that, we simplified the ISWM framework into two ‘overlapping triangles’ (Figure 6(c)) (Wilson et al., 2012b, 2013).

I led further development of the tool into the Wasteaware Benchmark Indicators (WABI) (Wilson et al., 2015a), providing indicators both for the three physical components of the system and for what are termed the governance aspects. Two of the three governance factors are sub-divided into separate indicators. *Inclusivity* focuses on stakeholders, specifically the *users* and the *providers* of the MSWM/WaRM service; while *sound institutions and proactive policies* are divided into indicators for the adequacy of the national legal and policy framework and for the degree of local institutional coherence. The third factor, *financial sustainability*, stands alone; each of these five WABI comprises five or six criteria (Wilson et al., 2015a), against which they are assessed qualitatively (Wilson et al., 2015b).

I was invited to lead the team preparing UNEP and ISWA’s inaugural Global Waste Management Outlook (GWMO) (UNEP and ISWA, 2015). We used the two triangles ISWM framework (Figure 6(c)) as the analytical framework; the graphical summary linked the physical components to the question WHAT TO DO?; and the governance aspects to HOW TO DO IT? The latter were visualised as four interconnecting cogwheels: responsibilities and partnerships (including all stakeholders), money matters (financial sustainability), proactive policies and sound institutions; and the need for a data revolution. As the literature had focused primarily on the technologies and the physical aspects (e.g. the excellent 1000pp textbook by Christensen (2011); see also reviews later under ‘Global South – investments in infrastructure’), the GWMO provides detailed guidance on both financial sustainability (Soos et al., 2015) and the other governance aspects (Rodic, 2015; Rodic and Wilson, 2017).

Publication of the GWMO was followed by the preparation of a series of Regional Waste Management Outlooks, covering Africa (Godfrey et al., 2019), Asia, Central Asia, Latin America, Mountain Regions, Small Island Developing States and West Asia (UNEP IETC, 2016–20).

The application of the ISWM analytical framework and specific tools used here is not the only option for analysing WaRM systems. For example, ISWM has been used in a variety of ways, for example, focusing on stakeholders (Abarca-Guerrero et al., 2013) or on recycling performance (Scheinberg and Simpson, 2015); alternative systems thinking approaches have been explored, building on a similar historical and drivers framework to that used in this this paper (Marshall and Farahbakhsh, 2013); and a PEST (Policy–Environmental–Socio-economic–Technology) thematic framework was developed using historical experiences in four Global North countries (the United States, Japan, Denmark, Australia) to explore factors limiting MSWM sustainability in the BRIC countries (Brazil, Russia, India and China) (Iyamu et al., 2020).

### Strategic planning for MSWM

Strategic planning is one important aspect no longer immediately visible in the simplified two triangles representation of ISWM (it appears three times at the criterion level, at both the national policy and local institutional levels, and as part of user inclusivity). This was where I started my career in 1974, when the state-of-the-art was optimisation models to minimise the costs of providing facilities. By 1990, the World Bank had recognised the need for ‘*appropriate and innovative planning tools to enable the authorities to select the right technology and institutional options . . . . which reflect the affordability of beneficiaries and the government’s capacity to manage*’ (Arlosoroff, 1991). When I led the development of a Strategic Planning Guide for MSWM for the UMP/SDC collaborative programme, the aim was to go one step further by integrating the stakeholders within a participative planning process (Wilson et al., 2000). The participative aspects were tested prior to publication in Ha Long, Viet Nam; and further elaborated through capacity building case studies funded by the UK government in La Ceiba (Honduras), Bamako (Mali) and Bangalore (India), resulting in a series of practical key sheets (Read et al., 2005). Although the primary context was the Global South, my team also used the participative methods in developing the first Waste Management Strategy for Northern Ireland (NI DoE, 2000).

## Governance factors

This section discusses some of the key changes in waste and resource management (WaRM) from 1990 to 2020, using the five WABI governance indicators as the sub-headings.

### Local institutional coherence

Municipalities are responsible for providing the MSWM service, and their ability to do that has long been recognised as an issue. One factor is simply the size and scale of the municipality – collection is generally assigned at an intermediate (‘district’) level of scale and disposal at a higher (‘metropolitan’ or ‘regional’) level, both of which may require inter-municipal cooperation. Another is the availability of the management and technical expertise to fulfil the responsibility (financial resources are considered separately, below).

#### Common institutional problems

By 1990, these issues had largely already been addressed in the Global North, but were still seen as major constraints in Global South. Among the common problems I observed in the 1990s (Wilson and Nair, 1992; Wilson, 1994) was the fragmentation of responsibilities for MSWM across many municipal departments; in one city, primary collection was carried out by street sweepers employed by the districts; secondary collection workers and the dumpsites came under the solid waste department; while the drivers and vehicles were run by the mechanical engineering department; the result was that no one individual or department had the responsibility or the authority to manage MSWM as a whole, and indeed no one knew how much the service was costing because the accounts were fragmented. Inadequate supervision was another issue; supervisors often had no means to move around their service area. Another was the relatively low level of management; technical skills and training were lacking; jobs were often based on political patronage rather than merit; I observed one city where a posting to the waste department was regarded as punishment for poor performance elsewhere in the city administration. Job rotation amongst senior administrators can also be an issue, for example, in the Indian civil service: I have met impressive people at international conferences or training courses, only to find a few months later that they have been moved to another posting.

#### Single point of responsibility

The World Bank highlighted weak institutions early on as a key constraint (Cointreau, 1982; Bartone et al., 1990; Arlosoroff, 1991). Institutional reorganisation to provide a coherent single point of responsibility alongside capacity building was a major component of every international MSWM consultancy project in the 1990s – to my surprise as a scientist, I found myself not only directing projects but also designated as the ‘institutional expert’.

#### Essential institutional functions (national and local)

Institutional issues are also important at the national level (considered under the WABI as part of the national framework). In the Strategic Planning Guide (Wilson et al., 2000), six essential institutional functions were distinguished, three predominantly anchored at national and three at local level. Having a national institution clearly designated and having the authority to implement SWM policy is essential – which agency that is, or what ministry it is part of, varies widely between countries and there is no ‘right’ or ‘wrong’ approach. The other national-level functions are the planner and the regulator; for the latter, independence and authority are particularly important, as is paying inspectors a good salary to reduce the temptation to turn a ‘blind eye’. At the local level, the three functions of client/employer, responsible for ensuring that services are provided; revenue collector; and the operator who delivers the service day-to-day, together comprise what is now called the ‘operator model’ (Soos et al., 2014; Wilson et al., 2017). More recently, a seventh function has been added, that of change agent, an organisation assigned to make a step change in policy happen in practice (Whiteman, 2010). In the 9DBs paper, we further refined the concept by dividing the ‘regulator’ into three separate functions, the environmental, technical and financial regulators, as each comes into focus at different stages of WaRM development (Whiteman et al., 2021).

### Financial sustainability

#### Affordability

MSWM costs increased rapidly as levels of service and control were raised in the Global North in the 1970s and 1980s, and this trend holds true in the Global South following in their footsteps. Any municipality must obtain sufficient revenues to cover the costs of providing the waste service; for financial sustainability, the costs should be borne by taxpayers, including households and other the service users. The World Bank has stated that the maximum affordable cost for a complete MSWM service ‘*is internationally accepted as 1–1.5% of average household spendable income*’ (World Bank Group, 2018). Using this rule of thumb, in high-income countries, the current costs of modern MSWM of around US$ 100–400 per tonne are easily affordable; but in low- and lower-middle-income countries, even their current costs for providing inadequate services are already pushing the limits of affordability (UNEP and ISWA, 2015; Kaza et al., 2018). So finding the funds to extend collection coverage and to raise control levels for disposal is challenging.

#### Costs of inaction

Mismanaged MSW has many adverse public health and environmental impacts (Cointreau, 2006; Velis and Mavropoulos, 2016; Ferronato and Torretta, 2019; Vinti et al., 2021). In the GWMO, we showed that the costs of inaction, that is, the economic costs to society from mismanaged waste, exceed the financial costs of collecting and managing the waste properly in the first place by a factor of 5–10 (UNEP and ISWA, 2015; Wilson and Velis, 2015). The problem is that dumping or burning their waste has little or no direct financial cost to the individual. Having said that, even the poorest communities will pay a small fee when they can see the benefits of a regular and reliable waste collection service in keeping their neighbourhood clean and their children healthy (Scheinberg et al., 2010b). So, charging households a direct fee that covers (most) costs of primary waste collection will often be feasible; while charging a direct fee for the collective benefits of safe disposal, and clean streets, which rest on a kind of social contract, is much more difficult and seldom works in low- and middle-income countries. Politicians are often unwilling to impose a direct cost on taxpayers for infrastructure when affordability of services is of concern and the benefits are not always clear or individual (Soos et al., 2015).

#### Revenue collection

Much internationally funded work to improve MSWM services in low- and middle-income countries has focused on the need to impose direct user charges. However, that is just one option available to a municipality for raising revenue to cover the costs of providing the waste service. Revenues can also be collected indirectly with property or other local taxes or with other utility charges (e.g. electricity or water bills); an unusual example is Kigali in Rwanda where a 95% fee collection rate is achieved by co-collection with fees for regular neighbourhood security patrols which people are willing to pay for (Kabera et al., 2019). Revenues can also be derived from general budgetary funds, from either municipal or national sources (Scheinberg et al., 2010b). Even when direct charging is used, payment has usually been at a flat rate; a variable rate, or pay as you throw, has only been the norm for household waste in the United States and Ireland, although other countries have recently experimented with this as a waste reduction measure (Welivita et al., 2015; Wilson 2018b).

All these mechanisms, or a combination of them, can work; the key is to ensure that the revenue collection systems match local administrative practices, practical realities and customs and traditions. I remember being very surprised on the first day of a week-long stakeholder workshop in 1991, when we were developing a national strategy for Poland when the country was seeking accession to the EU, to learn that collection coverage in Warsaw was only 80%, less than that in Bangkok; it was only later in the week that I understood that the municipal-owned waste company did offer collection services to all, but only 80% of building owners paid the waste fees and so received a service. This was a hangover from the former communist system, which did seem strange to me when compared to the then 12 (capitalist) EU Member States, all but two of which collected charges indirectly and so avoided the issue of non-payment (ERL, 1992c).

#### Pricing disposal

An important milestone in developing a sustainable WaRM system is to price waste disposal (Scheinberg, 2011). A landfill gate fee, or price allocated on the municipal balance sheet, provides not only secure revenue for disposal operations, but also stimulates an incentive for the 3Rs. More generally, larger waste generators should make their own arrangements, and pay the full economic costs, for ESM of their own wastes (the ‘polluter pays principle’). Smaller commercial waste generators often have the option to ‘opt-in’ to the MSWM system, so long as they pay their proper share of the costs. When new controls are being implemented, for both non-hazardous and for hazardous wastes, particular care is needed to ensure both that the infrastructure is available to do the right thing and that the environmental regulator is independent and well resourced, to counteract the strong financial incentives to dump waste illegally.

### Provider inclusivity

Even at the relatively low service and control levels being provided in the 1980s, many cities in the Global South were already struggling to cover the operating costs of their MSWM services, while often spending 20–50% of available municipal revenues (Arlosoroff, 1991). The World Bank recognised this early on, recommending a mix of approaches, including institutional strengthening and capacity building of the municipality and direct charging as discussed above, and ‘doing more with less’ through efficiency improvements (Wilson et al., 2000).

#### Public–private partnerships

The World Bank also actively promoted involvement of the private sector *(Bartone et al., 1990).* However, rather than using the term ‘privatization’ as for other utility sectors, the terms ‘private sector participation’ (PSP) and more recently ‘public–private partnership’ (PPP) have been used to reflect the nature of the MSWM operator model where the municipality remains responsible for provision of the service as the client/employer, but potentially delegates service delivery to a private entity. An early World Bank guidance document set out different models for PSP involving the formal sector, and identified three key success factors which must be present, namely *competition, transparency and accountability* (Cointreau, 1994). The CWG later published guidelines for municipal managers on involving micro- and small enterprises (the community and informal sectors), which is particularly important for extending collection to unserved areas (Haan et al., 1998); a detailed guidance pack for formal sector PSP including model contracts (Cointreau and Coad, 2000); guidelines for solid waste collection that benefits the urban poor (Coad, 2003); and lessons learnt from 23 case studies from both the formal, community and informal sectors (including failure cases), sub-titled *Avoiding Problems and Building on Successes (Coad, 2005)*.

**Figure 7.**
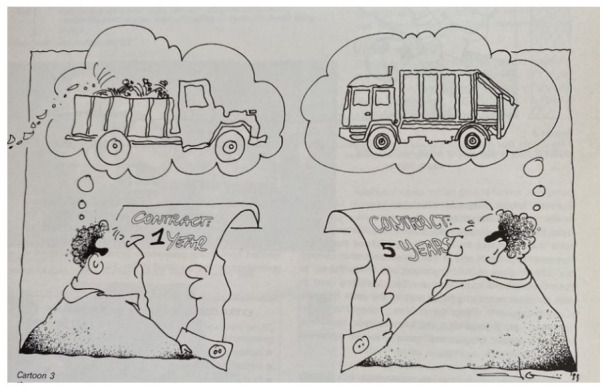
Successful PPP requires adequate contract duration. Anything more than 5 years allows the contractor to pay back a loan on new vehicles (which may or may not need compaction). A 1-year contract means using old and inefficient vehicles. Source: Cartoon by Dorsi Germann for Cointreau and Coad (2000). Reproduced by permission of SKAT. PPP: public–private partnerships.

One key lesson is the need for a balanced partnership. Yes, there needs to be requirements on the contractor to meet well defined, monitored and enforced performance measures; but also on the municipality, to pay on time according to an agreed schedule, and to set up the contract to enable the contractor to deliver the required performance. The project I led in Bangkok in 1987 focused on opportunities for PSP (Wilson et al., 1988), so we examined a previous ‘failure case’ where the city had been required to contract out collection services in three districts, and the consensus was that service standards had decreased. I later generalised the failings we identified, alongside experience from other projects, into recommendations for making PSP work (Wilson, 1994). What has stuck most in my memory is one municipal manager’s dismissal of PSP: ‘the contractor used bad old vehicles’; to which my response is neatly summarised by one of the excellent cartoons commissioned for the CWG guidance documents (Figure 7).

#### Public or private sector operation?

A major GIZ study revisited the issue of which operator models are more appropriate in a particular local situation (Soos et al., 2014), with two subsequent papers looking at the evidence base (Wilson et al., 2017) and providing decision support tools (Soos et al., 2017). This allows conclusions to be drawn from cumulative experience over some 30 years: PPPs are important for service delivery; an element of private service provision introduces competition into the service provider market, which can have a beneficial impact on reducing unit costs and improving attention to quality in service delivery; it is essential to ensure adherence to transparency and due process in public tenders. However, there is no evidence to show that private or public service provision or financing for MSWM is more frequent or is more efficient or beneficial than the other.

These conclusions are reinforced by a recent World Bank roadmap for MSWM sector reform: ‘*Private sector involvement makes sense only if there is sufficient and reliable financing available to the sector, and if the public sector has the capacity to provide meticulous contract enforcement and supervision of private activities. The private sector may improve efficiency on the margins and bring in private capital, but will rarely be able to solve larger sector issues; indeed, it may compound existing problems*’ (World Bank Group, 2018).

The best blend of public and private service delivery is what works best in the local situation. In the Global South, facilitating the inclusion of small-scale service providers, including community-based organisations, micro-enterprises and the informal sector, is often an effective way both to extend waste and recycling services to all communities and to maximise the opportunities for local job creation. The role of the informal recycling sector in the Global South is explored further below.

### User inclusivity

A major change over this period has been the increasing recognition of service users as a critical stakeholder group (Scheinberg et al., 2010b). Everyone has an interest in keeping their neighbourhood clean and their children healthy; equity of service provision requires that everyone receives a good MSWM service, irrespective of income. Actively involving the service users in the planning and delivery of the service is an essential element of sustainable system; while good performance against these criteria will not make the siting of new facilities straightforward, it is safe to say that poor performance is likely to make siting even more difficult (see discussion of NIMBY in Part A).

#### Behaviour change

Most initiatives to reduce environmental pollution or improve waste management require people (as householders or workers) to change their previous habitual behaviours. For waste, this first came to attention in the Global North when people were required to change from simply ‘throwing away’ their mixed waste to separating their waste at source into two or more components prior to collection to facilitate recycling (see below). But it is also relevant when waste collection is being extended to previously unserved areas in the Global South; people need to unlearn habitual behaviours to dump or burn their wastes, and learn new ones to store waste within the home and present it in the specified way for primary collection.

When I became an independent consultant in 2003, a major client over the next 10 years was the English environmental ministry Defra, advising on their evidence programme designed to underpin waste and resources policy (UK Defra, 2004, 2007; Wilson et al., 2007). Social science research was a major priority; in parallel, the UK governments prepared seminal reports on environmental behavioural change (Darnton, 2008; Southerton et al., 2011). It takes more than just providing information to change people’s attitudes and behaviours; making it easy for (i.e. *Enabling*) people to do the right thing, *Engaging* with communities, leading by *Example* and *Encouraging* people with both ‘carrots’ and ‘sticks’ (the ‘4Es’) are each important (UK Government, 2005); the 4Es model has been applied to WaRM (Cox et al., 2010; UNEP and ISWA, 2015).

#### Waste and gender

Another change has been the increasing recognition of MSWM as a gender issue (Seager et al., 2020). Women in most cultures are the persons largely responsible for managing waste within their household; the nature of the role is changing from self-management of waste (including by dumping or burning), to storing mixed waste for collection, to segregation of several fractions at source for separate collection; but all rely on women’s unpaid domestic labour. As waste management is modernised, women are also mostly excluded from the more lucrative levels of employment, including higher levels of administration and better-paid jobs (Seager et al., 2020). The same is generally true in informal recycling: men often have traditional rights to valorise higher-value metals, and women are ‘allowed’ to accumulate and sell lower-value plastics and textiles (Beall, 1997; Dias and Ogando, 2015; Wittmer, 2022). Investment often replaces ‘women’s work’ by ‘men’s work’ (Practical Action, 2021). Participatory planning for waste management represents a key opportunity to ensure gender mainstreaming and bring women’s knowledge, ideas, preferences and interests into waste system upgrading (Carpintero-Rogero and McGilchrist, 2015).

### National legislative and policy framework

By the 1990s, people were beginning to recognise that an approach focussed primarily on the ‘technical fix’, based on setting standards for technologies within a strictly enforced legislative framework, was not sufficient on its own. Despite waste prevention and recycling being placed in the waste hierarchy as preferable to recovery and disposal, landfill was still generally the cheapest option and thus dominant if the policy objective was simply to meet environmental standards at the least cost.

#### A new driver to reduce methane emissions from landfill

One issue was that the new ‘full control’ standards (Figure 2(b)) internalised only some of the ‘external’ environmental and social costs of disposal; both the remaining local costs and all the global costs from greenhouse gas (GHG) emissions were still borne by wider society. The latter came to the fore with adoption of the 1992 *UN Framework Convention on Climate Change* (UNFCCC). Methane is a powerful GHG, so national targets to reduce GHG emissions from a 1990 baseline under the subsequent 1994 *Kyoto Convention* provided a new driver both to collect and utilise methane emissions from landfill sites, but also to reduce waste generation, segregate wastes at source for separate collection (moving from the basic (mixed waste) level of collection service to improved or full service levels, see Figure 2(a)), increase recycling and divert biodegradable waste from landfill.

#### A wider range of policy instruments

Waste policy thus became more nuanced, with multiple objectives and constraints. Many different policy measures or instruments were considered or used in different countries to implement that policy. We compiled a database covering 25 developed countries and territories which underpinned reports for the UK (ERL, 1992a), Ireland (ERL, 1993) and Hong Kong (ERM, 1994) governments; I used that as the basis for a summary paper considering different types of ‘sticks’ and ‘carrots’ to move waste management up the hierarchy (Figure 5(B.2) (Wilson, 1996)). Targets (e.g. for recycling or landfill diversion) were often used, both in formulating the policy and as either an information mechanism (e.g. how is authority X or company Y progressing against the target) and/or a legislative stick (e.g. what is the penalty for missing the target). Other information dissemination and use mechanisms included various forms of ‘nudges’ to help people or companies change their behaviour, for example, through coordinated information campaigns or requiring local authorities or larger companies to prepare and publish waste management plans; but the ‘community right to know’ under the revised US Superfund SARA legislation shows that ‘regulation by embarrassment’ can be a powerful instrument (see part A; LaGrega et al., 1994).

#### Economic instruments

These can be divided into ‘carrots’ that provide a financial incentive to do the right thing (e.g. tax relief or direct support for research, development, demonstration or early adoption) and ‘sticks’ to correct distortions in the free market to force the desired action (e.g. various actions to charge the full economic costs of waste management; raw materials or product taxes; landfill and/or incineration taxes; and strict liability for environmental damage as in the US Superfund).

#### Legislative sticks

When economic instruments are underpinned by legislation, they could also be considered as legislative sticks. In addition to technical standards enforced through facility permitting and controls over waste transport, other legislative sticks include the mandatory provision of separate collection services, and even mandatory separation by the waste generator; bans on the landfill of certain types of waste; and bans on certain products that give rise to wastes, the most common being plastic bags (Godfrey, 2019; Nyathi and Togo, 2020; Muposhi and Mpinganjira, 2022). A number of classifications and reviews of policy instruments have been published (Wilson, 1996; UNEP and ISWA, 2015; Rodic and Wilson, 2017) (Xevgenos et al., 2015).

#### Extended producer responsibility

One policy instrument was already attracting attention in the early 1990s and demands separate mention. When responsibility for MSWM is assigned to the municipality, the implicit assumption is that they, and ultimately the householders who fund them either directly or indirectly, should bear all the financial costs. But by 1990, the proportion of MSW made up of packaging or other end-of-life products was increasing fast, so the question was being asked as to why these costs should not be paid by the ‘producer’ who placed the product on the local market. This concept, commonly referred to as extended producer responsibility (EPR), or as product stewardship in North America, has now been implemented in many countries on either a voluntary and/or mandatory basis, particularly for packaging and e-waste. Implementation has varied widely from the perspective of the producers (Cahill et al., 2011); for example, requiring them to organise and fund a parallel collection and recycling system in ‘first mover’ jurisdictions (Taiwan 1990–1997 (Lee et al., 1998) and Germany from 1991 (Wilson, 1996)); pay public authorities to meet the required targets on their behalf (e.g. France (Cahill et al., 2011) and Taiwan from 1997 (Lu et al., 2006)); or meeting the targets at the lowest possible cost, which in the UK meant focusing first on transit packaging from supermarkets rather than post-consumer packaging from MSW so that by 2018 they were paying less than 10% of local authority costs to recycle their packaging wastes (Wilson, 2018b).

Much of the legislative focus on EPR has been in the Global North, particularly in the EU. Four example case studies from the Global South were included in the GWMO (Rodic et al., 2015), focusing on packaging waste in Brazil (also known as reverse logistics, e.g. Guarnieri et al., 2020), South Africa (e.g. Godfrey and Oelofse, 2017) and Tunisia, and e-waste in China (e.g. Cao et al., 2016).

## Global North – From waste management to waste and resource management

### Rediscovering municipal solid waste (MSW) recycling

Recycling of clean industrial wastes where it is cost-effective to do so has always been a normal industrial activity. Recycling of MSW in the Global North had become by the 1970s an optional activity, seen as a good thing to do, but only when market conditions were favourable. This began to change when the costs of first controlled and later full control (environmentally sound management - ESM) landfill and incineration increased from the 1970s (Figure 2(b)).

#### Recycling as a competitive ‘sink’

The cost increase was particularly sharp in the United States, which faced a garbage crisis in the 1980s (Louis, 2004); new compliant disposal facilities were difficult to site, often far away and always expensive. Recycling was no longer something that was done only when market prices were high, but rather a competitive ‘sink’, which avoided the disposal costs for those wastes (Bartone, 1990). The result was that MSW recycling increased from 6% in 1970 to 17% in 1990 and 27% in 1995 (US EPA, 1997). This apparently very positive change was viewed rather differently by the established recycling industry; supply and demand were finely balanced, so a large influx of additional materials from MSW fundamentally changed the market dynamics (Scheinberg, 2003, 2011). For example, wastepaper exports from the United States were blamed for causing a glut on the world market and a steep drop in prices (Abert, 1992).

#### Policy measures to promote recycling

By the early 1990s, there were thus three distinct factors driving this ‘rediscovery’ of recycling from MSW: recycling as a competitive ‘sink’ to avoid high-cost disposal; diversion of biodegradable organic (‘biogenic’) wastes from landfill to reduce methane emissions and mitigate climate change; and moving waste up the hierarchy. Recycling includes both dry materials recycling (mainly paper, plastics, metals and glass) and organics recycling (often referred to as composting but includes the solid digestate from anaerobic digestion). Some 25 countries and territories, covering most of the Global North, were using or developing policy instruments to promote recycling (ERM, 1994; Wilson, 1996). Recycling sometimes involved separation from mixed wastes or segregation by the householder and delivery to a ‘bring’ point, but segregation at source and separate collection was beginning to expand. Canada was an early mover with their ‘blue box’ set-out container for dry recyclables, and the Netherlands with separate collection of kitchen and garden waste (Scheinberg, 2011). Such separate collection systems have become the norm in many countries, moving the level of collection service gradually from ‘basic’ (mixed waste) through ‘improved’ (two separate fractions) to ‘full’ (three or more separate fractions) (Figure 2(a)).

Different countries have used different mixes of policy instruments to drive up recycling rates. The EU set a policy framework, introducing packaging waste recycling targets under EPR in the 1994 *Packaging Directive* and increasing those over time; landfill diversion targets in the 1999 *Landfill Directive* and giving advance notice of a likely general MSW recycling target of 50% by 2020 which became mandatory in the 2008 *Waste Framework Directive (*ACR+, 2009*)*. Many Member States which already favoured high recovery systems (DB7, see Figure 1) (e.g. Austria, Belgium, Netherlands, Sweden, Denmark, France, Finland) employed a wide suite of policy instruments (DB9), including mandatory requirements for separation at source, landfill restrictions and bans, both landfill and incineration taxes and economic incentives for recycling (EEA, 2013).

By contrast, the UK continued to focus on fiscal instruments (moving from DB6 to DB8). Landfill tax rates for MSW and other biodegradable wastes increased gradually from £7/tonne in 1996 to £15 in 2004, then more steeply to £80 by 2014, then linked to inflation (£98.60/tonne in 2022/2023). A landfill allowance trading scheme enabled local authorities to trade their allocations of landfill space; this was well used from 2005 to 2008 while landfill tax rates were relatively low, but was discontinued early in 2013 (Calaf-Forn et al., 2014).

The United States did not adopt targets for recycling or landfill diversion at the Federal level, relying on tight regulation and market forces (DB8); however, some individual states did introduce recycling targets backed up, for example, by landfill bans (more like DB9) (Bartone, 1990; Ham, 1992).

Such legislative and economic instruments were also supplemented by social instruments as discussed earlier, many focused on promoting and supporting behaviour change as householders became used to separating their wastes. The UK also used a ‘change agent’, the Waste and Resources Action Programme (now an independent charity WRAP www.wrap.org.uk) which was charged by government to work with companies, consumers and the waste and resources sector to deliver the ambitious changes required. Most policy instruments focused on the supply side, that is, increasing the quantities of materials separated for recycling to meet targets. Some did focus on demand, for example, procurement policies requiring a minimum content of recycled materials in, for example, office paper, or quality standards for compost or digestate to be used for food production which in effect required the process input to be clean, source separated organic wastes.

#### Increased recycling rates

The net result of all this activity has been a dramatic rise in MSW recycling rates (dry materials plus organics recycling) in the Global North. From a baseline generally in single % figures around 1990, by 2019 one EU country (Germany) had reached a (combined) MSW recycling rate of 67%, 13 countries 40–60%, 10 more 30–40% with just 4 still at lower levels (EEA, 2021). Elsewhere, South Korea reports 60%, Australia 44% and the United States 32% (OECD, n.d.). These reported figures come with the usual ‘health warnings’ on data quality and variable definitions between countries (Greenfield and Woodward, 2016). One example is Japan, where the officially reported MSW recycling rate was 3% in 1983 (Bartone, 1990), increasing to 20% in 2019 (OECD, n.d.); however, it was noted that the definition of MSW is limited to the wastes actually collected by municipalities, excluding materials collected directly from households by recycling dealers, neighbourhood groups and schools (Tanaka and Matsumura, 1989; Abert, 1992); when these were included, the 1983 baseline was estimated, not at 3% but at 23% (Bartone, 1990).

#### Global markets for recycled materials

The market for recycled organics is local, but for dry recyclable materials (except glass) is global; this huge increase in supply has changed and disrupted world recycling markets. In one sense, the timing was lucky: since the 1980s, the Global North have outsourced much industrial production of the materials and goods they consume to the Global South; so, the fast growth in supply of materials for recycling coincided with rapid expansion in demand from countries such as China, whose new industries bought recyclable materials on the world market. Production of machine-made paper and board in China increased steadily from 1.7 million tonnes in 1965 to 28 million tonnes by 1995; after a slight dip, growth took off again in 2000, to reach 117 million tonnes by 2015. Primary plastics production similarly grew steadily from a low base, reaching 1 million tonnes around 1990 and 11 million tonnes by 2000, then accelerating again to 78 million tonnes by 2015 (NBS China, n.d.).

A high proportion of materials collected for recycling in the Global North were thus exported, with China dominating the global market (Velis, 2014). China imported around 30 million tonnes of waste paper in 2012 (Magnaghi, 2015); while total world trade in scrap plastic increased from around 4 million tonnes per annum (Mtpa) in 2000 to 14–16 Mtpa between 2010 and 2015 (of which 8 Mtpa went to China), before falling sharply to 7 Mtpa by 2018 (BIR, 2021) and around 3 Mtpa by 2021 (OECD, 2022a). Our study of the waste plastics trade in 2012 showed a competitive market, with buyers in China and the US out-bidding each other for a limited supply of high-quality PET bottles for recycling (Velis, 2014); the resulting high price for their raw material was partly responsible for putting one recycler in the UK out of business (Wilson, 2018c).

But not all materials collected for recycling are actually recycled (Velis and Brunner, 2013), so that perhaps 5–10% by weight of the exported materials became ‘rejects’ to be disposed of in the importing country. Much of the higher quality imported materials went to rapidly modernising paper and plastics manufacturers; but some imported materials may also have gone to local, informal recycling industries, operating to relatively low environmental standards. Waste criminals saw an opportunity, and some exported materials were dumped or burned. As a result, a 2019 amendment to the Basel Convention in effect banned exports of certain plastics and mixtures of plastic waste from the Global North to countries that may not have the resources, infrastructure, legislation or enforcement capacity to manage residues.

China first tried to control imports by raising quality specifications, for example, through Operation Green Fence in 2012; when in 2018 those specifications became so stringent as to exclude all post-consumer grades of recycled paper and plastics (the ‘China ban’), it sent shock waves around municipalities and the waste sector in the Global North. Since then, many nations in the Global South have followed suit with stricter import specifications or ‘bans’ (Cook and Velis, 2022). It became urgent to rethink MSW recycling, to develop a new, more sustainable, global supply chain paradigm (see Part C).

### Edging towards waste prevention

#### Terminology: 3Rs –> 9Rs

A lot of effort has been given to promoting recycling in the Global North; what about waste prevention? It has been generally accepted since the 1970s that prevention is the preferred option, sitting at the ‘top’ of the waste hierarchy (Figure 5). The top rungs were expressed for many years as reduce, reuse, recycle, commonly known as the ‘3Rs’. These have been further refined over the years, suffering what could be called inflation of the ‘Rs’; for a period, ‘5Rs’ or ‘6Rs’ were fashionable; options for the circular economy are now often cited as the ‘9Rs’, of which there are actually 10, arranged into three groups: smarter product use and manufacture – R0 Refuse, R1 Rethink and R2 Reduce; extend lifespan of product and its parts – R3 Reuse, R4 Repair, R5 Refurbish, R6 Remanufacture R7 Repurpose; and useful application of materials R8 Recycling, R9 Recovery (including energy recovery) (Potting et al., 2017; Morseletto, 2020). So ‘waste prevention’ can be sub-divided into 8 of the 10 ‘Rs’. Even then, one can still generate additional Rs, for example, by splitting rethink into redesign and rent, the latter also called product service systems where one acquires, for example, the service of washing clothes rather than a washing machine so that the supplier remains responsible for keeping the machine working for as long as possible rather than the current motivation to sell a new one (Gottberg et al., 2010).

#### Source reduction of industrial and hazardous waste

For hazardous waste, one could argue that technical approaches to ‘source reduction’ did receive priority from the 1970s and 1980s, in response to high costs for proper treatment and, particularly in the United States, strict liability rules for landfill (US OTA, 1983; Wolf, 1988). Early programmes were termed variously reduction (e.g. UNEP and UNIDO, 1991), pollution prevention (e.g. World Bank et al., 1995), clean technology (e.g. ISWA WGHW, 1989), cleaner production and strategic waste prevention (e.g. OECD, 2000). The UNEP-led cleaner production programme was launched in 1989 and established a network of more than 100 Cleaner Production Centres around the world (UNEP, 1998).

However, it is difficult to measure the success of such initiatives: how data are reported has changed over time. Also, any reductions may be due, not to real waste prevention, but rather to the offshoring of dirty heavy industry and manufacturing from the Global North to the Global South, moves that were motivated both by cheaper labour but arguably also by less stringent environmental and health and safety standards. Some central treatment plants have now closed due a shortage of hazardous wastes, for example, AVR Chemie, a joint venture between local and central government and eight multi-nationals, opened a modern rotary kiln incinerator in Rotterdam in 1987, which closed in 2005.

#### Food waste prevention

One waste stream which has been targeted for waste prevention is food wastes. WRAP, the UK change agent charged with facilitating a step change in the 3Rs, published a seminal report, *The food we waste*, highlighting that a third of the edible food that people buy is not eaten (WRAP, 2008). This shocking statistic galvanised action, with similar data being confirmed for other countries in the Global North, alongside similar losses in the supply chain in the Global South (Parfitt et al., 2010; Wang et al., 2021). It has been estimated that worldwide 1.3 billion tonnes of food are grown but not eaten (FAO, 2011), accounting for 9% of global GHG emissions (FAO, 2013). SDG target 12.3 is to halve global per capita food waste and losses by 2030; progress is being monitored by the Food Waste Index (UNEP, 2021a).

#### Prevention of MSW

For MSW, increasing recycling did take priority in the early decades. Early work on prevention was voluntary, often led by communities and NGOs (e.g. (Fishbein and Gelb, 1992). In Europe, EU Member states did have long-term advance notice of a requirement to put in place initial national waste prevention plans, for which the eventual deadline was in December 2013 (Bartl, 2014; EEA, 2014). When I advised Defra on their waste and resources evidence programme, my personal focus was on waste prevention, including many individual research and two review projects (Cox et al., 2010; Sharp et al., 2010a, 2010b; Wilson et al., 2012a). I guest edited a special issue of WM&R in March 2010, where our editorial proclaimed ‘*waste prevention: its time has come*’ (Wilson et al., 2010). With hindsight, that may have been premature, but hopefully by 2020, it was beginning to be true.

## Progress in the Global South

A major focus of Part B has been the shift from the ‘technical fix’ to a more integrated approach addressing also the ‘governance’ factors. Progress in the Global South in addressing the institutional and financial constraints and other governance factors has already been discussed under ISWM. Attention turns now to progress on the ‘physical’ aspects of collection, disposal and the 3Rs of MSWM (Figure 6(c)).

### Progress in extending collection and controlled disposal

#### 1990s baseline

In the 1990s and 2000s, progress in extending collection and controlled disposal in the Global South was relatively slow. Indeed, World Bank did not update a summary statement on their website’s MSWM homepage for some 15 years up to the late 2010s: ‘*30% to 60% of all urban solid waste is uncollected and less than 60% of the population is served*’, while also asserting that open dumping of collected waste was still the norm (UNEP and ISWA, 2015). This corresponds to most urban areas sitting in development bands DB2 or DB3.

#### Recent advances in data availability

The first authoritative attempt to produce a global database on MSWM was World Bank’s *What a Waste (WaW) (Hoornweg and Bhada-Tata, 2012)*. This used available data, from official national statistics and published sources, which at the time were both inconsistent and unreliable; in the absence of weighbridges at many sites, data were built from estimates. When the CWG on MSWM in low- and middle-income countries was discussing our approach to a definitive report on the state of SWM in the World’s cities for UN-Habitat, our team leader Anne Scheinberg joked: ‘*there are only two types of data on SWM, bad data and worse data*’. As a result, we chose to gather our own detailed data for 20 cities using a consistent methodology based on ISWM (Figure 6(c)) (Scheinberg et al., 2010b; Wilson et al., 2012b), which was later developed into the Wasteaware Benchmark Indicators (WABI) (Wilson et al., 2015a).

#### Demonstrating progress in selected cities

**Figure 8.**
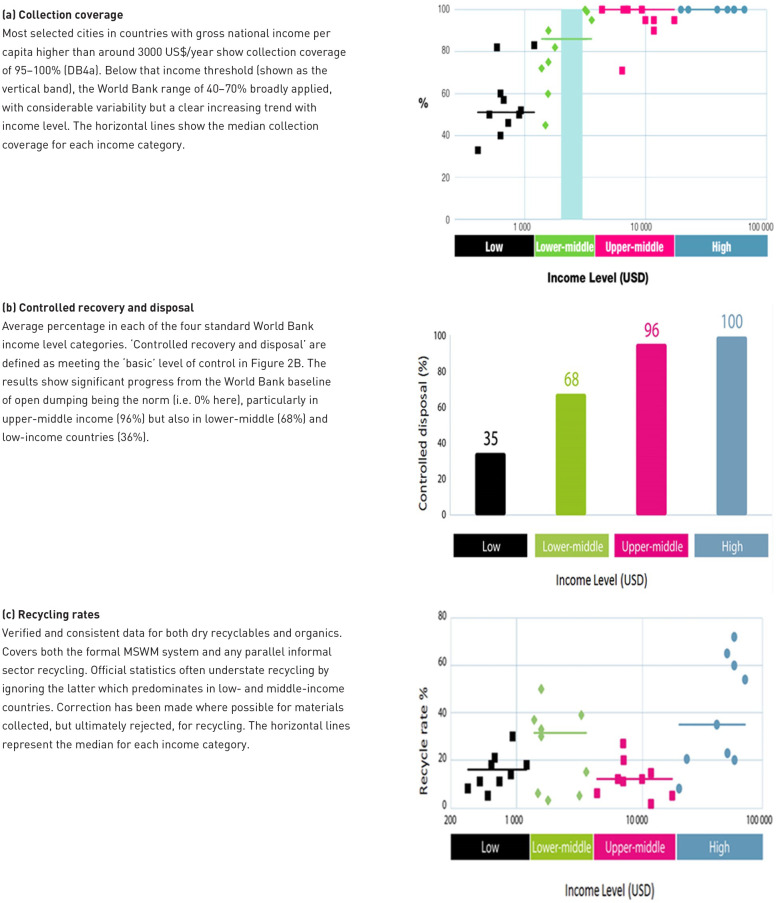
Quantitative Wasteaware Benchmark Indicators (WABI) for selected cities, showing (a) collection coverage, (b) controlled recovery and disposal, and (c) recycling rates. Indicators are plotted against income level of the country (gross national income (Atlas method) per capita) on a logarithmic scale. Plots cover the 39 cities for which data were available in May 2014. Selection of the cities may favour those with better performance; also performance is lower in rural areas; so it is expected that these cities outperform the national average. However, they do illustrate that substantial progress had been made. Data source: Wasteaware – University of Leeds. Figures prepared for UNEP and ISWA (2015). Wasteaware Benchmark Indicators: (Wilson et al., 2015a). An updated analysis relating this data set to a wide range of socio-economic indicators is available (Velis et al., 2023).

When I led work on the inaugural Global Waste Management Outlook (GWMO), we not only compiled a global database based largely on national statistics, but also supplemented that with an analysis of the latest WABI database which then covered some 40 cities (UNEP and ISWA, 2015). The results showed both a more nuanced and a more positive progress report than implied by the World Bank website (Figure 8). For collection coverage, there was a clear income threshold, mid-way through the lower middle-income category, above which most selected cities achieved 95–100% (DB4a); and below which the World Bank range of 40–70% broadly applied (Figure 8(a)). For controlled recovery and disposal, these data again show significant progress from the World Bank baseline of open dumping being the norm: particularly in upper-middle income (96%) but also in lower-middle (68%) and low-income countries (36%) (Figure 8(b)). Summarising in terms of the development bands (Figure 1), the selected cities in low-income countries had generally reached DB2/3, lower-middle income DB3/4 and upper-middle income DB4/5.

#### Billions of people still lack basic services

Using these data as a guide, my ‘back-of-the-envelope calculation’ for the GWMO was that at least 2 billion people worldwide were without access to MSW collection, and more than 3 billion lacked access to controlled disposal facilities. Thus, despite considerable progress, the conclusion was that MSWM was still a global challenge for the 21st century and a global call for action was issued (UNEP and ISWA, 2015; Wilson and Velis, 2015). The World Bank published an update of their global database (WaW2.0) (Kaza et al., 2018); when I use their data to redo my calculations, the estimates are higher, around 2.7 billion people without access to waste collection services. The original GWMO results were reported variously as a global waste crisis or emergency; my updated calculations reinforce that headline.

#### Fates of MSW

The WaW2.0 data for fate of MSW based on national statistics (Kaza et al., 2018) remain ambiguous as to what proportion of land disposal is uncontrolled, controlled or environmentally sound management (ESM) (Figure 2(b)), and often omit informal recycling. But a best estimate of fates worldwide around 2015 based on that dataset showed around 29% by weight uncollected (split roughly 2:1 between ‘wild’ dumping and open burning). The 71% of waste by weight that is collected was split: 9% open burned, 21% open dumping, 13% controlled landfill, 10% full control (sanitary, ESM) landfill, 7% ESM incineration, 7% (dry) recycling and 4% composting or anaerobic digestion (Gómez-Sanabria et al., 2022). The average rate of controlled disposal as a proportion of the total waste collected in the Global South is around 30% (i.e. 13% controlled landfill/43% by weight of collected waste disposed by non-ESM methods); while this is less than the data for selected cities reported in the GWMO (Figure 8(b)), it is still significantly better than the World Bank historical baseline of 0%.

#### Waste and the SDGs

The GWMO was prepared in parallel to the sustainable development goals (SDGs) (UN, 2015). To keep the number of SDGs manageably low, neither SWM nor air pollution were included as a high-level SDG, instead being dispersed across targets within several SDGs. GWMO developed what can best be described as five ‘global waste targets’, that correspond to a ‘virtual waste SDG’. The first two targets are, *by 2020, ensure access for all to adequate, safe and affordable solid waste collection services* (GW1), and *eliminate uncontrolled disposal and open burning* (GW2). These correspond to the two parts of SDG indicator 11.6.1, and are intermediate steps on the way towards GW3, *by 2030 ensure the sustainable and environmentally sound management of all wastes*, which corresponds for MSWM to SDG target 12.4 except for adjustments to make the target date less unrealistic (2030 rather than 2020). The two remaining targets are straight equivalents, GW4/SDG 12.5 on substantially reducing waste generation through prevention and the 3Rs, and GW5/SDG 12.3 on halving food waste. Both the original GWMO (UNEP and ISWA, 2015) and subsequent papers (Rodic and Wilson, 2017; Wilson, 2021) tabulate the linkages between the GW targets and the SDGs, showing direct and measurable links with six, direct links with a further six, and indirect links with the remaining five SDGs (Wilson, 2021).

### 3Rs and informal sector recycling

#### 3Rs in Asia

The 3Rs (reduce, reuse, recycle) has been adopted as the ‘banner’ for efforts to improve waste and resource management (WaRM) policy in Asia and the Pacific since 2005; the inter-governmental Ha Noi Declaration in 2013 (Regional 3R Forum, 2013) established sustainable 3R Goals for Asia and the Pacific for 2013–2023. Two reports provide the baseline in 12 countries (Visvanathan et al., 2008), and mid-term progress towards the goals (Regional 3R Forum, 2018).

Having said that, most recycling in the Global South is still informal, which has likely always been commonplace (see part A, 1970 baseline), initially pre-dating and then operating in parallel to and often ‘invisible’ to the formal MSWM sector (Wilson et al., 2006). So this sub-section focuses on informal sector recycling.

#### Categories of informal recycling

**Figure 9.**
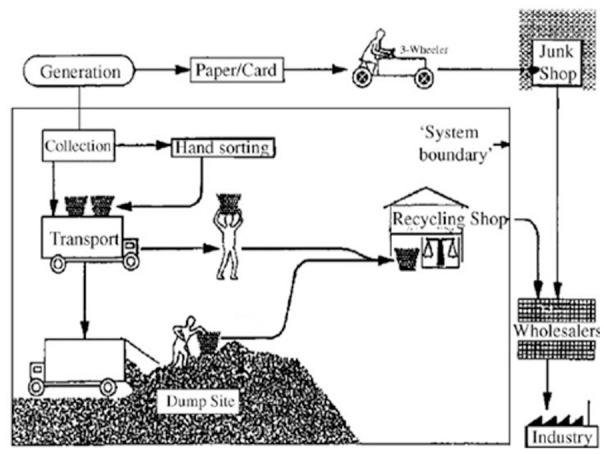
The recycling and MSWM system in Bangkok in 1987. An important element was the ‘three wheelers’, itinerant waste buyers going door-to-door in tricycle carts to purchase source segregated materials from households or household servants. Once the waste has been collected and is within the MSWM system, it is picked over at collection points, by municipal workers sorting waste on vehicles and at the dumpsite. Source: I first drew this graphic as part of the baseline study for a project in 1987 (ERL and IBH, 1988; Wilson et al., 1988) and have reused it frequently since (e.g. (Wilson et al., 2006). Further information on recycling in Bangkok is available (Muttamara et al., 1994). MSWM: municipal solid waste management.

The usual image is of pickers scavenging at dumpsites (e.g. Birkbeck, 1978; Beall, 1997), but Figure 9 from a 1987 study in Bangkok shows a much more complex system. This includes itinerant waste buyers going door-to-door to purchase source segregated materials from households or household servants, which is also a ‘customary system’, for example, on the Indian sub-continent (Lardinois and Furedy, 1999). Once the waste has been collected, it is also picked over at collection points (e.g. Huysman, 1994) and by municipal workers sorting waste on the vehicles (Muttamara et al., 1994) (Cointreau et al., 1984).

#### Recycling rates

Official statistics on recycling often ignore the informal sector; however, when care is taken to include them, the results are perhaps surprising (Figure 8(c)). The data there confirm earlier work (Wilson et al., 2009; Scheinberg et al., 2010a) to suggest a range in the Global South of perhaps 5–45%. Even higher rates are possible when organics recycling (see below) is included; for example, an estimate for the Zabbaleen recyclers in Cairo showed 74% when they were feeding the residual organic wastes to pigs (Scheinberg et al., 2010a).

#### Changing attitudes to the informal sector

There has been increasing focus on how to build on this foundation while also addressing the obvious problems such as child labour (e.g. ILO, 2004, 2022) and unsafe working conditions (Cook and Velis, 2020; Zolnikov et al., 2021). One challenge was/is to change the attitude of municipal authorities and politicians from often negative, ranging from embarrassment and neglect, through collusion to active persecution (Medina, 2000); to positive engagement, support and inclusion as recycling experts and legitimate stakeholders (Wilson et al., 2006). An important step is to recognise the economic, social and environmental benefits from informal recycling – reducing the quantities to be collected and disposed of by the formal MSWM system by say 20% potentially saves a mega-city $ hundreds of million in both annual operating and in capital costs (Scheinberg et al., 2010a; Wilson et al., 2012b); while creating tens of thousands of jobs provides livelihoods (SDG 7) (Linzner and Lange, 2013) and alleviates poverty (SDG 1) (Morais et al., 2022).

#### Inclusion/integration of the informal sector

Much work has focused on how best to include the informal sector alongside the formal MSWM system to form an integrated WaRM system. Academic papers include, for example, calls to re-conceptualise the system (Gutberlet, 2010; Dias, 2016; Velis, 2017; Wittmer, 2022), and several reviews (Ezeah et al., 2013), one focusing on barriers and success factors (Aparcana, 2017), another on ‘formalisation’ efforts (Morais et al., 2022). The grey literature includes syntheses from comparative case studies (e.g. Gerdes and Gunsilius, 2010; Gunsilius et al., 2011; Scheinberg, 2012), one of which provides a decision-makers’ guide (Scheinberg and Savain, 2015) and another an operational guide specifically aimed at planning for the inclusion of those dumpsite pickers displaced by new ESM disposal sites (Cohen et al., 2013).

**Figure 10.**
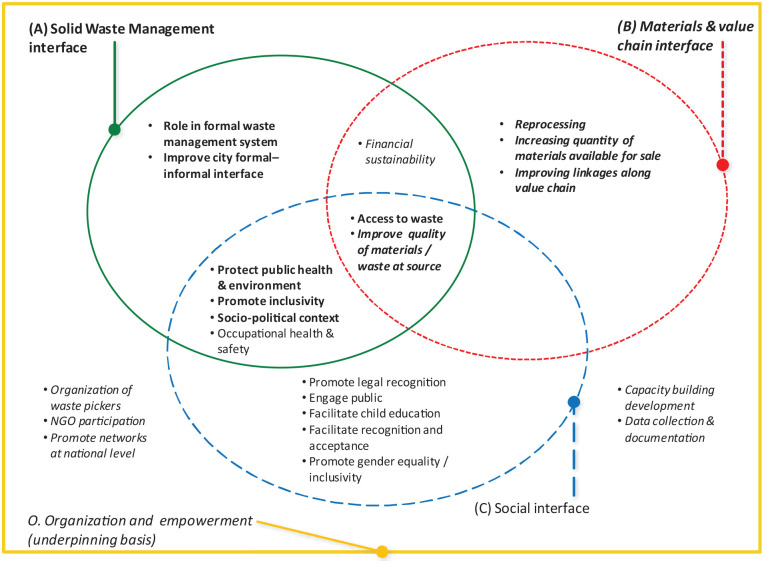
The InteRa tool for designing a local programme for the inclusion/integration of the informal recycling sector (IRS) alongside the formal SWM system. The overall analytical framework assigns groups of interventions to four categories, comprising three primary interfaces between the IRS and (A) the SWM system, (B) the materials and value chain and (C) society as a whole; underlain by a fourth, (O) organisational aspects. To maximise the potential for success, IRS inclusion/integration/formalisation initiatives should consider all four categories in a balanced way. Increased attention should be paid to their interdependencies (where the ‘sets’ intersect) which are central to success, in particular the IRS having access to source separated waste and to affordable micro-finance. The different fonts show how the groups of interventions have been allocated to the four categories: (A) bold; (B), bold italics; (C) plain text; (O) italics. Source: (Velis et al., 2012).

Figure 10 shows schematically one tool for designing a local inclusion programme; a successful plan needs to select appropriate actions from each of four components (Velis et al., 2012). Everything is underpinned by organisation and empowerment of the recyclers (marked ‘O’ in Figure 10), in particular organisation into cooperatives or micro-enterprises to ensure their voice can be heard and facilitate negotiation with the municipality and other stakeholders (e.g. Medina, 2000; Samson, 2009, 2015; Gutberlet, 2015; Godfrey et al., 2017). Another aspect here is promoting recognition and acceptance (Chaturvedi, 2003), as, for example, in Brazil and elsewhere in Latin America where informal recyclers have legal status (Gerdes and Gunsilius, 2010; Fidelis et al., 2020; Marquez et al., 2021); this overlaps with the interface (‘C’ in Figure 10) between the informal sector and wider society. Other actions address the interfaces with the formal MSWM system (A), and the materials and value chain (B) of which the recyclers form a vital part (Jaligot et al., 2016).

#### The importance of separation at source

Figure 10 also highlights some critical interventions which cut across the categories, particularly access to waste which has been separated at source. One comment has stuck in my memory from the opening keynote address to an international conference in the 2000s, by an advocate who had been working with their local recycling community for many years; ‘when my friends ask me how I can work with such dirty people, I tell them that it is not the recyclers who are dirty but us’. And that is true: if we don’t mix the dry recyclable materials with messy food waste, both fractions will stay clean. The recyclers can work in cleaner and healthier conditions; separate larger quantities to sell at higher prices; and earn a better livelihood so that they can afford to send their kids to school rather than working in the family business. Increasing the collection service level in the Global South beyond the basic mixed waste collection is returned to in Part C.

#### Organic waste recycling

Most focus tends to be given to the recycling of dry materials within the waste. But MSW composition in the Global South is generally 50+% food and other putrescible organic materials. Segregating this fraction at source and collecting it separately not only facilitates the clean recycling of the dry materials, it also opens the door to clean organic recycling, much higher recycling rates, another income stream for recyclers and benefits to local farmers (Ricci-Jürgensen et al., 2020). Organic recycling can be carried out at the household, community or decentralised levels, with considerable advantages over larger, more formal schemes. The many options (Lenkiewicz and Webster, 2017; Lohri et al., 2017; Zabaleta et al., 2020) include direct use as animal feed (Scheinberg et al., 2010b; Boumans et al., 2022); decentralised composting (Ali and Rouse, 2004; Zurbrügg et al., 2004, 2005; Rothenberger et al., 2006); anaerobic digestion to generate gas for cooking as well as a compost product (Gunnerson and Stuckey, 1986; Mueller, 2007; Vögeli et al., 2014); breeding insects such as black soldier flies as a source of protein and fertiliser (Diener et al., 2011; Dortmans et al., 2021; Deng, et al., 2023; ur Rehman et al., 2023); and the production of char as a cooking fuel (Lohri et al., 2016; Zabaleta et al., 2018).

### Investments in infrastructure

#### Upgrading to controlled recovery and disposal

There was considerable focus in the 1990s on how to upgrade disposal standards stepwise in the Global South (Figure 2(b)). The South Africa government was a leader, publishing two editions of their Minimum Requirements for Waste Disposal by Landfill (DWAF, 1998). A useful book provided both technical guidelines and the wider ‘governance’ context (Ali et al., 1999); World Health Organization (WHO) published guidance on upgrading existing dumpsites (Rushbrook, 2001); many papers were presented at conferences and a few in peer-reviewed literature (e.g. (Blight, 1996; Rushbrook, 1999)). An authoritative World Bank – WHO handbook focused mainly on best practice (full control, ESM) technology, but included sections on minimum acceptable standards, and a chapter on additional provision for co-disposal of difficult wastes (Rushbrook and Pugh, 1999).

Other reports included several on collection and the full range of the waste hierarchy (Diaz et al., 1996; UNEP IETC, 1996); UN Habitat and others on collection (Coffey, 1988; Rouse and Ali, 2002; Coffey and Coad, 2010); World Bank on incineration (Rand et al., 2000a) and composting (Hoornweg et al., 1999); and GTZ on mechanical-biological treatment (Kebekus et al., 2000). A review of other organic recycling sources is in the previous section.

#### Accessing investment funding

Extending collection coverage, upgrading recovery and disposal standards to a ‘basic’ controlled level, and building recycling rates from the existing (informal sector) baseline, all require investment. Affordability has earlier been identified as a key constraint in the Global South. Many municipalities struggle to meet operational costs, so need help with investment costs to extend and improve MSWM infrastructure and services. Established financing sources include national funds, commercial credits and international or bilateral development grants and loans.

International financial institutions such as World Bank and other multilateral and bilateral agencies have been active both in technical assistance, project preparation and funding of vehicles and infrastructure. A review of World Bank funding for MSWM from 1988 to 1997 reported a similar number of projects (71) to the previous 10 years, but with double the funding ($1044 million) (Bartone, 2000; Gopalan and Bartone, 1997). Most projects funded waste collection vehicles, about half sanitary landfill development, with just three being dedicated MSWM projects.

#### Case studies: Project preparation disrupted by ‘magic solutions’

Experience with the larger, more integrated and ambitious projects was ‘mixed’ at best in the 1990s. For example, I led consultancy teams providing technical assistance to prepare two proposed World Bank dedicated MSWM projects, one for local government units outside of Metro Manila in the Philippines, and the other for Colombo in Sri Lanka. Despite positive results from the feasibility studies, neither project was taken forward. One issue was lobbying by local environmentalists and academics against the landfill component, arguing instead for a ‘zero-waste’ solution based on the ideal of 100% recycling. However, what finished both projects was the ‘magic solution salesmen’. In the Philippines, they sold a so-called ‘BOT technology’ (build–operate–transfer (BOT) is one contractual form of public private partnership) to the first local government unit who were about to sign up, claiming it was cheaper and environmentally preferable to landfill; the salesman later contacted our office in Manila: ‘we’ve got a contract, can you provide the technology?’

In Sri Lanka, the technology really was ‘magical’: mixed MSWM was to be shredded, diluted 1:20 with water, ground to a wet pulp and then dried to a fine powder, which was to be sold as a ‘fertiliser’ for agriculture. That time, I got a direct call a couple of months later from the project proponent inviting us to join their team; when I asked polite questions as to how they addressed the thermodynamic and contamination issues, he accused me of trying to steal their technology and hung up. In both cases, the result was the same: the World Bank cancelled their investment, the alternative ‘commercial’ project quickly fizzled out and the country was back to square one.

During our 4 years in Colombo, the city had used several unsatisfactory temporary dumpsites while waiting for the new World Bank funded landfill site. When that project was cancelled, they continued to use a sequence of uncontrolled disposal sites surrounded by housing. A methane gas explosion and landslip at Bloemendhal in 2009 miraculously caused no fatalities; not so when a landslip at the by then 50 m high Meethotamulla site on Sinhalese New Year, 14 April 2017, completely destroyed 68 houses and killed at least 32 people (MacDonald, 2019).

#### When are the conditions right to invest in ‘high-tech’?

These examples may appear extreme, but technology salespeople have always tried to push their products in the Global South. My project teams were often summoned to the Mayor’s office and asked for advice on the pile of ‘offers’ on their desk, selling all sorts of technologies, mainly waste to energy but also state-of-the-art landfill and reconditioned compaction vehicles for collection. A useful set of initial screening questions would be whether the technology is proven: at commercial scale? For MSW? For wastes with high organic and moisture content? The two examples suggest a simple rule of thumb: if a solution looks too good to be true, it probably is.

Partly in response to the experiences recounted above, the World Bank supplemented their technical guidance on technologies with specific guidelines aimed at politicians, mayors and other decision-makers; covering landfill (Thurgood, 1998) and incineration (Rand et al., 2000b). The latter set out numbers of mandatory key criteria, any one of which should stop the incinerator project even before any detailed feasibility work. These essential pre-conditions included:

➢ a mature and well-functioning waste management system has been in place for a number of years – near universal collection coverage and all waste which cannot be recycled is disposed of at controlled and well operated landfills;
➢ authorities have the capacity and resources to meet their responsibility to control, monitor and enforce operations;
➢ lower calorific value must average at least 7 MJ/kg, and must never fall below 6 MJ/kg in any season;
➢ the community can afford and is willing to pay increased treatment costs.

In terms of the development bands (Figure 1), a city should be approaching or have reached DB5, with rates of collection coverage and controlled disposal around 95%; in current parlance, MSWM should meet SDG 11.6.1 within a good governance framework.

#### Decision-makers’ guides

Despite this apparent clarity, aggressive marketing of technologies has continued to cities with low collection coverage, low rates of controlled disposal and weak institutional arrangements. Yet another round of guidance has been prepared, this time aimed primarily at decision-makers. Table 2 provides a ‘guide to the guides’, indicating for each the range of technologies covered and of the decision support tools provided. For incineration and other ‘high-tech’ technologies, these current decision-makers’ guides confirm the earlier pre-conditions listed above, and add at least one more: residents actively sorting their waste at source allowing the recovery of recyclable materials and control of waste not suitable for incineration.

**Table 2.** A guide to recent decision-makers’ guides to technology selection for MSWM in the Global South. The decision support tools provided include narrative guides to how the technologies work; analysis/tabulation of their pros and cons; checklists of useful questions for the Mayor and their advisors to ask the salespeople to screen appropriateness of the technology and vendor for the local context; and colour-coded criteria for a rapid first assessment – any single ‘red’ light indicates a mandatory pre-condition which must be met before proceeding to a more detailed evaluation.

Scope	Agency	Source	Narrative guide	Pros and cons	Checklists/useful questions to vendors	Red/amber/green pre-conditions	Comments
Integrated MSWM system	USEPA	(US EPA, 2020)	X	X	X		Covers governance factors, collection, recycling, controlled disposal and technologies for recovery and disposal.
Four proven technologies for MSWM	World Bank	(Kaza and Bhada-Tata, 2018)	X	X	X		Separate guides to sanitary (full control, ESM) landfill, composting, anaerobic digestion and incineration with energy recovery. Some mention also of pyrolysis/gasification.
Alternative waste-to-energy technologies	GIZ	(GIZ, 2017)	X	X		X	Covers five technologies: landfill gas, co-processing in a cement kiln, anaerobic digestion, incineration and pyrolysis/gasification. Colour-coded matrix tool to examine local readiness for each technology.
	UNEP-IETC	(UNEP IETC, 2019)	X	X	(X)		Relatively generic to all waste-to-energy technologies, more focus on incineration.
	CWG	(Whiteman et al., 2016)			X		Rapid assessment tool applicable to any waste-to-energy technology. Detailed questions/checklists under multiple criteria.
Individual technologies: waste-to-energy incineration	IGES Centre Collaborating with UNEP on Environmental Technologies (CCET)	(Liu and Nishiyama, 2020)	X	X	X	X	Three guidelines in a standard format. Each provides a useful graphic showing colour-coded pre-conditions which need to be met before even considering the technology.
Composting		(Kawai et al., 2020)	X	X	X	X	
MBT		(Ishigaki and Liu, 2020)	X	X	X	X	
Co-processing in a cement kiln	GIZ-LafargeHolcim	(GIZ and LafrageHolcim, 2020)	X	X		X	Detailed technical guidelines + summary for decision-makers + assessment matrix tool

ESM: environmentally sound management; MBT: Mechanical-biological treatment; MSWM: municipal solid waste management.

#### Selecting appropriate technologies

Table 2 does include guidance for decision-makers on collection, landfill and organic recycling/recovery technologies, all of which may be appropriate in a wide range of local situations. The review of guidance earlier in this section is still relevant when the aim is to upgrade from no or limited control to basic control (Figure 2(b)). A useful rule of thumb to decide when a technology is appropriate in a particular local situation uses the ISWM framework (Figure 6) and again derives from South Africa. This is the so-called ‘Pent As’ evaluation tool: a technology should be institutionally Appropriate, technically Applicable, legally Achievable, financially Affordable and environmentally Acceptable (Pilusa and McCarthy, 2016; Gower-Jackson, et al., 2018).

#### Inability to fund operating costs

Some donor-funded projects failed after implementation. One example was the Jam Chakro landfill in Karachi, completed as a sanitary landfill to full control standards in 1996, but which quickly reverted to being an uncontrolled dumpsite. This was blamed on an ‘invasion’ by informal pickers seeking to make a living, who tried to maximise the few remaining recyclables (mainly metals) reaching the site by burning the waste. However, the pickers had not been considered in project design; the city did not have the institutional capacity to manage the site as it had been designed; nor could they afford the operating costs (Rouse, 2006).

The city not being able to afford the costs of proper operation was/is a concern for many international financial institutions, who are often constrained to funding capital but not ongoing operating costs. World Bank pioneered the use of the Kyoto Protocol’s Clean Development Mechanism to incentivise cities to upgrade existing uncontrolled landfills and invest in new sanitary landfills (Dulac, 2010). Capital costs and some initial operating costs were met by official development assistance, while collection and utilisation of landfill gas generated carbon credits under the Clean Development Mechanism. Payments were made retrospectively on providing verification of carbon savings achieved, so after the initial period, the city could rely on receiving an annual payment which would in effect fund the following year’s operating costs, so long as the site continued to be operated properly and generate gas. The downside of the Clean Development Mechanism was the need for costly and bureaucratic verification, which limited its rollout to other waste projects, although by the time the scheme ended in 2012 it had begun to be used also to fund composting, anaerobic digestion and plastic recycling projects (Dulac, 2010).

#### Capacity building

For several years from 2000, the World Bank decided that many countries were not yet ready to accept investments in MSWM, and focused instead on building that capacity. One example I was part of involved eight countries on the southern and eastern coasts of the Mediterranean Sea. Regional guidelines for decision-makers and aids to implementation (e.g. explanatory documents, tools, case studies and train-the-trainer materials) covered aspects of ISWM including policy, legal and institutional, finance and cost recovery, private sector participation (PSP), and public awareness & community participation (Belherazem et al., 2005). A follow-up project was funded by the German agency GIZ, using their long-term technical assistance model aimed at building local capacity so that the project achievements continue after funding ceases.

#### How much development finance goes to SWM?

Our study for ISWA of official development finance over the 10 years to 2012 showed that cumulatively US$4 billion was directed to SWM; as a proportion of total annual funding, this tripled over the period, from 0.1% to just 0.3%. SWM spending was split as $2.8 billion in loans from development banks for investment mainly in collection and engineered landfill capacity, and $1.2 billion as 3000 smaller grants. The grant funding was split as 25% for purchase of collection vehicles and containers, and to provide SWM in the aftermath of conflicts or natural disasters; and 75% to increase local skills and capacity and provide other technical assistance on issues such as the informal sector recycling, PSP, cost recovery, awareness raising and climate change. Of the cumulative $4 billion, two-thirds went to just 10 middle-income countries (the largest being China, with $510 million in 12 loans); only 10 low-income countries (all in sub-Saharan Africa) received grants or loans of more than $4 million, representing just 5% of the total funding (Lerpiniere et al., 2014). Lending most to the upper-middle-income countries who can best afford it, and who likely have more capacity to absorb the investment, makes good sense from a financier’s perspective; but does little to tackle the continuing waste emergency in the lowest income countries.

### Progress in emerging economies

This sub-section addresses countries referred to in the 1990s as ‘emerging economies’: China, Eastern Europe and the newly independent states of the former Soviet Union.

#### Rapid stepwise progress in China

China has already been seen to be a rather special case; its 1970 baseline of near universal MSW collection, state-run recycling enterprises and apparently circular ‘garbage farming’ seem to have placed it at least in development band DB5 (Figure 1); but by 1990 had reverted to DB4a on collection and DB3/DB2 on controlled recovery and disposal. The period since then has been one of unprecedented change, documented by the invaluable online collection of China Statistical yearbooks (NBS China, n.d.). The proportion of the population living in urban areas increased from 19% in 1980 to 36% in 2000 and 61% in 2020 (UN Population, 2018). Economic growth took off from 1990, showing more than 40-fold increases in both industry and services by 2014, while gross domestic product per capita increased 20-fold. The demise of the old ‘circular’ system thus coincided with an exponential increase in the quantities of MSW collected, from 31 million tonnes in 1980 to 191 million tonnes in 2015 (Wilson, 2018c).

China in 1990 thus faced considerable challenges, and adopted a similar stepwise approach to Europe and the United States 20 years earlier, first bringing waste recovery and disposal under basic control, then gradually ramping up standards (Zhang et al., 2010; Dorn et al., 2012; Lu et al., 2017). As in the Global North, the initial stages focused on technologies; the volume of new policies relating to MSWM in China was relatively low in the 1980s and 1990s, before expanding rapidly through the 2000s and 2010s (Chu et al., 2019).

Since I joined Imperial College London as a Visiting Professor in 2000, I have supervised the theses of many Chinese Masters students. One compared the MSWM systems in Beijing and London as each prepared to host the Olympic Games. She found that in Beijing, 2282 garbage farming deposit points around the city had become uncontrolled disposal sites by 1990; the first large transfer station and controlled landfill opened in 1994; 15 basic control landfills and two incinerators achieved a controlled recovery and disposal rate of 91% in 2003 (Guo et al., 2005). MSW generation in Beijing doubled between 2000 and 2007, creating severe capacity issues (Wang and Wang, 2013). Compositional analysis showed a decline in coal ash from 52% in 1990 to 6% by 2006, with a corresponding rise in food waste from 25% to 63% (Li et al., 2009).

Nationwide, the controlled recovery and disposal rate increased from 52% in 2005 to 94% in 2015 and 99% by 2019 (Liu et al., 2020); up to 2015, the number of landfills increased from 356 to 640 and incinerators from 67 to 220 (Mian, et al., 2017; NBS China, n.d.; Wilson, 2018c). Thus, in the 2010s, China surpassed DB4b, transitioned rapidly through DB5 due to a considerable tightening of environmental regulation and control on waste recovery and disposal facilities (Hodgkinson et al., 2022); and has now become established within DB7 thanks to a sustained focus on technology adaptation and innovation (Whiteman et al., 2021).

Up to the 1980s, China had a state-run recycling system operating in parallel to the formal MSWM system. As the formal recycling system ran down, the vacuum was taken up by a growing informal recycling sector. Official MSWM statistics measure wastes ‘as collected’, not ‘as generated’ (Linzner and Salhofer, 2014). This means that any materials sold directly by the generator either to informal collectors or to the remaining formal recycling industry are reported elsewhere (Xiao et al., 2018). The informal recycling sector was significant: estimates suggest that it provided a livelihood to 300,000 people in Beijing (Li et al., 2009) and 3.3–5.6 million in total (Linzner and Salhofer, 2014); and achieved an MSW recycling rate ‘as high as 27.8%’ (Liu et al., 2020) or in the range 17–38% (Linzner and Salhofer, 2014). The value chain into which the informal recyclers sold their materials was also largely ‘informal’, including dedicated recycling hubs such as the plastics recycling village of Luwang in Shandong province, which was closed by government inspectors in 2017 (Wilson, 2018c).

Both the ‘China ban’ on import of recyclable materials from the Global North and the closure of unregistered and environmentally non-compliant local recycling facilities indicate an official policy shift towards formal recycling of MSW. Separate collection of at least three segregated fractions (dry recyclables, household hazardous wastes, residual wastes) is now mandatory in major cities; with a strong recommendation to add a fourth segregated fraction, food waste (Liu et al., 2020). In the 2020s, China is implementing stronger policy instruments than in much of the Global North, which appears to reflect a transition towards DB9.

So for MSW management, China appears to have moved from crisis to a relatively modern, well-developed system in 30 years, over which time quantities have increased fivefold; that is impressive. Unfortunately, the rapid nature of that progress is being used against them. For example, the first definitive reference on the quantities of plastics entering the oceans (Jambeck et al., 2015) named China as the largest source, accounting for 28% of the World total. On digging into the detailed modelling and assumptions, one finds that the data used were from 2004, when they estimate that 78% of waste in China was mismanaged. That estimate would have been pessimistic then, but given rapid recent progress in China, continuing to use such estimates today is simply wrong.

#### Moving direct to EU standards in new Member States

Up to the late 1980s in many eastern European countries, municipal-owned enterprises provided waste collection to all who paid for the service, while standards of disposal remained low. Recycling was often ‘compulsory’ through state-run recycling companies, which were quickly disbanded when the countries achieved independence from 1989 onwards. Anecdotal reports at the time in the former East Germany suggested that waste quantities suddenly ‘doubled’, which if it was true would suggest that the previous recycling rate had been around 50%. Both the eastern *Länder* in the newly unified Germany and those countries which applied for accession to the European Union were bankrolled to move in one step to full ESM control standards. By 2020, all the ‘new’ EU Member States sit firmly in DB5 and are working to improve the level of both recovery and disposal standards and of collection services at the same time (Figure 2). Many are finding it challenging to raise levels of segregation at source and rebuild recycling rates, to achieve again or to exceed their previous baseline and to meet EU targets. So, perhaps the much-derided state ‘subsidy’ for recycling was not after-all so misplaced; had the pre-transition waste management systems in eastern Europe been understood (and defended) better and quicker, then the circular economy movement might well have started there.

#### Two case studies: Is an interim step necessary?

I led two major technical assistance initiatives around 2000 to prepare European Bank for Reconstruction and Development projects to fund landfills as part of integrated MSWM, one in Kharkiv, Ukraine and the other in Kaliningrad, the Russian oblast surrounded by EU countries on the Baltic coast. My teams carried out very detailed work, first preparing a participative MSWM strategy followed by feasibility studies including some 24 supporting reports covering the baseline situation, and legal, technical, institutional, financial, economic and social aspects. The recommendations in each case were positive, but neither project was implemented. In Kharkiv, the final loan agreement was voted down by the local Duma, who chose to make the high cost a party-political issue; why should a large part of the credit available to the city be used for a new landfill site when there are lots of other infrastructure priorities (schools, hospitals, etc.)?

The high-cost barrier here is worth exploring further. The older EU Member States had all moved stepwise, first upgrading to the basic (controlled landfill) level, before ramping up to improved and full (ESM) control levels (Figure 2(b)). When countries in eastern Europe applied for accession to the EU, they were inundated with both technical assistance and preferential grant funding to move in one step to full ESM control standards; the non-accession countries could receive technical assistance but only limited grant funding, generally from individual Member States. Nevertheless, the Bank’s mandate requires them to ensure that all funded projects meet full EU environmental standards (some EU bilateral donors apply the same seemingly moral but in practice progress-defeating principle to all developing countries: ‘we should not be funding projects elsewhere which would not be acceptable at home’). So it is easy to see the viewpoint of local politicians; either provide similar grant funding or allow stepwise progression as the EU did in the 1970s and 1980s, initially building a landfill to controlled or improved control levels which is more affordable.

## Part C: 2020–2030 – Reflections on Present and Future Priorities

The evolution of waste and resource management (WaRM) over the last 50 years has resulted in substantial change, at least in the Global North. In this final part, I first discuss recent developments which may (or may not) turn out to be the ‘tipping point’ which sees waste management emerge onto the world stage as a political priority. I then use some of the lessons learned over the past 50 years, and my work over the last 15 years on global priorities, to reflect on how continuing evolution of WaRM should be shaped through the 2020s (and beyond).

## SWM emerges onto the global agenda

### A changing landscape

#### Population, economic and waste growth

It was reported in the introduction that the World was already quite a different place in 1970 than it had been in 1950, and those changes have since intensified. Between 1970 and 2020, world population doubled to 7.8 billion (UN Population, 2022), with the proportion living in urban areas increasing from 37% to 56% (UN Population, 2018). There were just three megacities (population over 10 million) in 1970, New York, Tokyo and Osaka; by 2030 that is expected to reach 40, with 32 in the Global South (UN Population, 2018). Living standards as measured by average world gross domestic product per capita in constant 2015 US$ have more than doubled, from US$4900 in 1970 to $11,000 in 2019 and 2021 (there was a dip in 2020 due to COVID-19) (World Bank, n.d.). Consumption has increased in parallel, leading at least initially to exponential increases in resource extraction and use, emissions and waste generation (e.g. OECD, 2002; Hoornweg, et al., 2013). There is some evidence that the Global North is now beginning to ‘decouple’ waste growth from economic growth (Velis et al., 2023), but economic development will inevitably mean that waste per capita continues to increase in most of the Global South (Hoornweg et al., 2015; UNEP and ISWA, 2015; Velis et al., 2023).

When increases in population, migration from rural to urban areas and waste generation per capita are multiplied together, MSW generation in many African and Asian cities is doubling every 15–20 years (UNEP and ISWA, 2015). So even if a city manages to double the number of people receiving a collection service, and the weight of waste going to controlled facilities, every 15–20 years, the overall weight of uncollected waste and of wastes going to uncontrolled dumping and burning would remain roughly the same; on the one hand, admirable progress would have been achieved; but on the other, it would be ‘running to stand still’.

#### Changing types and composition of waste

The composition of MSW has changed markedly with the growth of consumerism. Around 1950, the dominant fractions were dust from coal ash used for domestic heating and cooking, and putrescible organics from food and garden waste. By 1970, post-consumer wastes were already growing, including packaging, newspapers and magazines, and other short-lived products. The proportion of packaging in MSW as generated has grown rapidly, now ranging from 20 to 40% by weight (or perhaps double that by volume) depending on country income level (Karak et al., 2012; UNEP and ISWA, 2015). Plastics were only beginning to be measured in MSW composition surveys in 1970 (<1%) (UK DoE, 1971); plastics now comprise 7–12%% by weight across a wide range of income levels, with levels up to 20+% commonly reported (Karak et al., 2012; UNEP and ISWA, 2015; Das, et al., 2019).

Waste electrical and electronic equipment, here called e-waste, has been an issue for some time and quantities continue to grow rapidly, as does the literature; reviews include Widmer et al. (2005), Ongondo et al. (2011), Shittu et al. (2021), Murthy and Ramakrishna (2022), Omondi et al. (2022) and Shahabuddin et al. (2023). Types of e-waste are also evolving; for example, one hazardous component was cathode ray tubes, for example, from televisions, but that is now largely a ‘legacy’ waste; while, for example, end-of-life solar panels (Rathore and Panwar, 2022) and electric car batteries are expected to grow rapidly. Some e-waste is categorised as hazardous wastes and will arise in all countries whether or not they have the infrastructure for environmentally sound recycling, recovery and disposal. E-waste is just one component of household hazardous waste (El-Khateeb, 2022), which has long been on the agenda in the Global North (see 1990 baseline; (Slack et al., 2005; Inglezakis and Moustakas, 2015)) but is increasingly receiving attention in the Global South (Manggali and Susanna, 2019).

#### COVID-19

Another issue which burst upon the World in 2020 is the COVID-19 pandemic. MSWM was one of the essential utility services which had to be maintained despite lockdowns. Contamination of discarded waste was seen as one potential infection route (Han et al., 2022), which inter-alia disrupted world recycling markets (e.g. GA Circular, 2020); and both infection control and testing resulted in a sharp increase in quantities of single-use plastics for disposal. Several review papers already collate experiences and make recommendations to improve future resilience of the waste management system (Mahyari et al., 2022; Singh et al., 2022).

#### Healthcare waste management

Including COVID-19 here does point up one notable omission from this historical review, which has not attempted to be comprehensive. That is the important issue of healthcare waste management, which would merit its own historical review (e.g. Kenny and Priyadarshini, 2021; Liang, et al., 2021; WHO, 1999, 2014; Townend, et al., 2009).

### Global action on plastics pollution

#### Data on plastics

Global plastics production has grown tenfold since 1970 (Geyer et al., 2017). Focusing in on the period 2000–2019, OECD report a doubling in both plastics production (from 234 to 460 Mt) and plastic wastes (from 156 to 353 Mt); outpacing economic growth by 40%. Only 9% of plastic waste was ultimately recycled; 19% incinerated; ~50% sanitary landfilled; leaving 22% disposed of in uncontrolled dumpsites, open burned or leaked into the environment. Leakage to the environment in 2019 amounted to 22 Mt (OECD, 2022a). More than 60% of plastics is used for short term mainly single-use applications (Resource Futures, 2018) – 40% packaging, 12% consumer products and 11% textiles (OECD, 2022a).

#### Ocean plastics

Concern over the accumulation of both macroplastics and microplastics in the environment, particularly in the oceans, is not new (Barnes et al., 2009; Rochman et al., 2013). Publication of the first global database on MSWM (WaW; Hoornweg and Bhada-Tata, 2012) provided the basis to estimate the weight of macroplastics entering the oceans from mismanaged waste; looking at populations within 50 km of the coast, initial estimates were around 8 Mtpa (Jambeck et al., 2015), with an additional 2 Mtpa from longer rivers (Lebreton et al., 2017; Schmidt et al., 2017). Images of marine animals ensnared by discarded plastic rings, or plastics in the stomachs of dead seabirds, were commonplace, but the tipping point in terms of global public opinion was (yet another) television programme, by Sir David Attenborough, broadcast around the world in 2017/2018 (Rapid Transition, 2019).

#### Mismanaged solid waste as the major source of plastics leakage

By coincidence, the week after the first release of the Attenborough programme, I was co-leading a workshop on marine litter, as one of three strands at the annual Development Finance Forum organised by the German state-owned development bank KfW, titled ‘Oceans 21 – Solutions for a Sustainable Marine Future’ (Mundus Maris, 2017). The meeting focused on improving the management of MSW in developing countries as the best way to ‘turn off the tap’ of plastics entering the oceans (Velis et al., 2017). My best estimate was that reaching the first two global waste targets of extending waste collection to all and eliminating uncontrolled disposal (i.e. 95+% on SDG indicator 11.6.1) should at least cut in half the weight of plastics entering the oceans (CIWM and Wasteaid UK, 2018). OECD report that macroplastics account for 88% by weight of plastic leakage into the environment, mainly resulting from inadequate collection and disposal; with microplastics from a range of sources such as tyre abrasion, brake wear or textile washing, accounting for the remaining 12% (OECD, 2022a).

#### Huge increase in activity

The new global focus on plastic wastes has led to an exponential increase in research; a Scopus search for waste, plastic and (marine OR ocean) yielded 917 documents to the end of 2017, increasing to 4800 by the end of 2022. Contributions include reviews of global impacts (e.g. Beaumont et al., 2019); emissions inventories (Zhu and Rochman, 2022; Cottom et al., 2023) and scenarios to reduce plastics pollution (Lau et al., 2020; Pew and Systemiq, 2020; OECD, 2022b). The latter require major improvements at all stages of the plastics life cycle to reverse the ‘business as usual’ scenario of continuing exponential increases.

All this has put better waste and resource management firmly on the global agenda. The Global Waste Management Outlook (UNEP and ISWA, 2015) highlighted the ‘global waste emergency’ of more than 2–3 billion people lacking basic services, but it has taken the plastics crisis to mobilise serious action. Many ‘plastics-funded’ initiatives have been launched to extend waste collection to unserved communities in the Global South; early lessons learned in Brazil, Chile, India and Indonesia have been collated (Danielson et al., 2020). Much funding has gone to southeast Asian countries seen as potentially leaking most plastics to the oceans (Jambeck et al., 2015); for example in Indonesia, projects STOP (STOP, n.d.; Stuchtey et al., 2019) and CLOCC (CLOCC, n.d.).

#### Towards a legally binding instrument

Pressure has gradually built for global action (e.g. de Wit et al., 2019), leading in March 2022 to agreement at the 5^th^ UN Environment Assembly (UNEA-5) to ‘end plastic pollution’ and forge an international legally binding instrument by 2024 (UNEA, 2022a). The resolution addresses the full lifecycle of plastic, including its production, design, recycling and disposal. The challenge now is to ensure that the final agreement recognises the need to tackle the collection, recycling and disposal of MSW in lower-income countries in an integrated manner, and not just plastic wastes in isolation (Silva-Filho and Velis, 2022). The case is being made strongly for a just transition (UN-Habitat and NIVA, 2022), and for a place at the negotiating table for the informal recycling sector (GRID-Arendal, 2022).

### Waste and climate

#### Historic focus on methane

Global heating has been a major driver for better SWM since the 1990s, initially focusing on reducing methane emissions from the decomposition of biogenic wastes in landfill which the Intergovernmental Panel on Climate Change (IPCC) has identified as responsible for some 90% of GHG emissions from the narrowly defined end-of-pipe waste sector. Efforts in the Global North to increase landfill standards beyond basic control did include the capture and combustion and/or utilisation of landfill gas, and in Europe diversion of biogenic waste from landfill, so when the IPCC’s Fifth Assessment Report (AR5) reported on 2010 GHG emissions (IPCC, 2013), the contribution of the narrowly defined end-of-pipe waste sector had already been reduced to 3–5% of the global total.

#### Indirect carbon savings from the 3Rs

However, better SWM, and the transition to WaRM and the circular economy through the 3Rs, mitigates GHG emissions across the economy, not just in the end-of-pipe waste sector. IPCC accounting conventions credit, for example, the 90% savings from recycling a tonne of aluminium to the metals industry, or a reduction in the 9% of global GHG emissions attributable to producing food which is thrown away without being eaten (FAO, 2013) to the food industry.

#### Estimated mitigation potential

My work for GWMO (Wilson et al., 2015c) and subsequently (Wilson, 2022) suggests one can have high confidence that the potential contribution of better waste and resource management to climate mitigation is large and needs to be actioned if the world is to have a chance of limiting global heating to 1.5–2.0°C. Any numerical estimate of the contribution is necessarily uncertain, my best guess being perhaps 15–20+% of total global GHG emissions. This is consistent with the mitigation potential being championed by others in terms of closing the circularity gap (70% of global GHG emissions is due to materials handling and use; Circle Economy, 2022); or the circular economy (Diaz-Bone et al., 2021); or resource efficiency (WRAP, 2021). ISWA have been active in promoting the links between waste and climate, and WM&R published two special issues in advance of COP15 in Copenhagen in 2009 (Savino, 2009).

### Open burning of waste

One impact of mismanaged solid waste that has until recently ‘slipped under the radar’ is open burning, which contributes both to plastics pollution and to climate heating.

#### Extent of open burning

The first attempt to estimate the extent of open burning and model emissions used the What a Waste (WaW) database (Hoornweg and Bhada-Tata, 2012); the result was a headline grabbing >40% total global MSW (970 Mtpa), with 29% of that in China (Wiedinmyer et al., 2014). However, the assumptions made (all urban wastes in the Global South, and all rural wastes everywhere, available for open burning; and 60% of available wastes actually burned) bore little relation to the real world. A more recent study used the WaW2.0 database (Kaza et al., 2018) and more reasonable assumptions, reporting a still shocking but more believable estimate of 16% of the global total of MSW open burned (394 Mtpa) (Gómez-Sanabria et al., 2022).

#### Local and global impacts

Open burning is now attracting more research interest (Ramadan et al., 2022). Both activity and emissions have been measured at a local level (e.g. Reyna-Bensusan et al., 2018; Krecl et al., 2021). Health implications have been modelled using the Wiedinmyer activity data, with conflicting estimates of around 300,000 premature deaths per annum either globally (Kodros et al., 2016) or just in India and Nepal (Saikawa et al., 2020). Laboratory studies by my PhD student at Imperial College London, Natalia Reyna, have highlighted that two plastics (PET and polystyrene) are responsible for around 80% of black carbon emissions from open burning of MSW (Reyna-Bensusan et al., 2019). Black carbon is a short-term climate forcer many times more potent than methane; our best estimate of the potential contribution of open burning of waste to total GHG emissions was relatively high, at 2–10% (Reyna-Bensusan et al., 2019). However, the estimated global warming potential of black carbon compared to CO_2_ has since been reduced in IPCC’s AR6 report by a significant factor (IPCC, 2021); using that and the revised best estimate of activity levels discussed above (Gómez-Sanabria et al., 2022), an updated estimate might be 0.5-1.0%.

#### Campaign to end open burning

An authoritative review of the health and safety implications (Cook and Velis, 2020; Velis and Cook, 2021) has led to an international campaign to end the scourge of open burning (Powrie et al., 2021; Mebratu and Mbandi, 2022). The plastics component of waste is a particular concern, and the new treaty to stop plastics pollution (UNEA, 2022a) must include measures to phase out open burning (Velis, 2022), which is an integral part of both global waste target GW2 (stop open dumping and burning) and achieving 95+% on SDG indicator 11.6.1.

### International science-policy panel on chemicals, waste and pollution prevention

Throughout my long career, waste management, in general, and MSWM, in particular, have always been rather unfashionable; an essential utility service, but taken for granted unless things go wrong; and relatively low down on the list of political priorities. So it is ironic that UNEA-5 took not just one significant step to change that with the global treaty to end plastics pollution (UNEA, 2022a), but two. They also resolved to establish an international, independent ‘Science-policy panel to contribute further to the sound management of chemicals and waste and to prevent pollution’ (UNEA, 2022b), to be modelled on the IPCC.

#### Scope of the panel

The scope and modus operandi of the new panel are currently being negotiated, with a target start date in 2024. The key issues to be addressed by the panel will be fiercely debated, as will the interfaces with other international environmental agreements including those on hazardous wastes, chemicals (UNEP, 2022) and now also plastics. Some priorities may be relatively clear-cut, for example, obtaining better data, hazardous wastes (both elaborated below), pesticides, trans-boundary and urban air pollution, plastics pollution. From this paper, I would argue that open burning of waste and mismanagement of MSW (inter alia as the major source of plastics entering the oceans) must also be included.

I would also make the case to extend the scope beyond the limited list of wastes which were brought under legislative control in the 1970s. Major political exclusions then were mining and quarrying wastes and agricultural and forestry wastes, which are dominant in weight terms (UNEP and ISWA, 2015); some pose serious health and environmental risks which need to be addressed at the global level, for example, mine tailings (Kossoff et al., 2014).

Another issue is the definition of ‘science’. From my own experience, the evidence base to inform policy making (Wilson et al., 2007) must be interdisciplinary, including not just STEM (science, technology, engineering and medicine) but also the social sciences. The whole lifecycle needs to be addressed, not just end-of-life waste management; also the full range of both technical aspects and ‘governance’ factors in the ISWM methodology. It is also important to recognise and address potential conflicts, such as that between chemicals control and recycling in the circular economy (see Rethinking recycling below).

#### Improving waste data

Obtaining better data, through both direct monitoring and modelling, will be central to the panel’s role. This will be particularly welcome for waste management, where both lack of availability and unreliability of data have been recurring themes of this paper. A particular issue is the difficulty of measuring waste that is not collected and managed, which includes much open burning and plastics leakage to the oceans.

A key element of the consistent data collection underpinning the assessment of progress with MSWM in the Global South in Part B above was the use of a material flow diagram for each city (Scheinberg et al., 2010b). The use of material flow analysis (MFA) in waste management was pioneered by Paul Brunner’s team in Vienna (Brunner and Rechberger, 2005, 2016); their STAN freeware is widely used (Cencic and Rechberger, 2008; TU Wien, n.d.). A version of MFA, termed the Waste Flow Diagram and adapted to estimate leakages to the environment from the MSWM system (GIZ et al., 2020), forms part of the Waste Wise Cities Tool to measure performance against SDG indicator 11.6.1 (UN-Habitat, 2021a). The Waste Flow Diagram is also one of a suite of four MFA tools to measure leakages of plastics from the MSWM system into the environment (University of Leeds, n.d.), two at the local and two at the global level (Cottom et al., 2023).

Estimating leakages from the system must be complemented by routine collection of data on waste that is managed within the system: if you don’t measure it, you can’t manage it.

When waste has been collected, one criterion for the basic level of control (Figure 2(b)) is that it is weighed and recorded (UN-Habitat, 2021a). But better data are an issue not just in the Global South. To take the UK as an example, universal weighing of all MSW collected was only achieved in 1993, around a century after near universal collection. Reliable and consistent data on commercial and industrial wastes are still not available, although a multi-year project led by the four environmental regulators to develop a standardised digital tracking and reporting system has recently led to proposals for new regulations (UK Defra, 2022).

The huge opportunities for the waste and resources sector provided by the fourth industrial revolution (Mavropoulos and Nilsen, 2000; Kanojia and Visvanathan, 2021), including ‘big data’ and artificial intelligence (Ihsanullah et al., 2022), are only just beginning to come on stream.

#### Hazardous wastes

These wastes will certainly appear on the new science-policy panel’s agenda. What needs to be done in terms of technology for the environmentally sound management of specific types of hazardous waste has been well established in the Global North since at least the 1990s, as have the governance frameworks to make that happen in practice. However, globalisation has resulted in the outsourcing of heavy industry and manufacturing, and thus much of the world’s industrial hazardous waste generation, to the Global South. But hazardous wastes are generated in all countries, not just the emerging industrial nations; current examples include e-wastes, oily wastes, lead-acid batteries, solvents, pesticides and a myriad of household hazardous wastes; while legacy wastes include polychlorinated biphenyls from electrical equipment, asbestos and now-banned and out-of-date pesticides. As reported in Part A, attention began to focus on building the capacity for sustainable hazardous waste management systems in the Global South as early as the 1980s/1990s; the Basel Convention has continued that work (UNEP Basel, n.d.). Considerable progress has been made for example in China (Duan et al., 2008; Kanwal et al., 2022; Su et al., 2022). Elsewhere, legislation is often in place; the priority now is to upgrade and make robust the governance systems, including independent regulatory agencies with sufficient skilled and well-paid staff, to allow effective implementation and thus provide the confidence that industry needs to invest in the required ESM recovery and disposal facilities. So, much remains to be done to provide an integrated and sustainable local and global hazardous waste management system serving the needs of all countries.

### Exponential growth in peer-reviewed literature

The increasing political profile of waste and resource management (WaRM) is reflected in the volume of the peer-reviewed literature. A fivefold increase in the total number of published papers on marine plastic waste between 2017 and 2022 has already been noted. But there is also a longer-term trend: two broad Scopus searches, on ‘municipal solid waste management’ and ‘urban waste management’, gave similar results over time: up to 1980 – ~250 papers; 1990 – 750; 2000 –2000; 2010 –5000; 2020 – 12,000; 2022 – 14,700. Recent reviews on various specific topics generally restrict their search to papers from the last 5, 10 or 20 years. My basic thesis here is that one needs to understand how WaRM has evolved in the past, both to plan confidently for the future and to avoid ‘reinventing the wheel’. Hopefully, this paper can contribute by appearing in current searches, and providing an extensive reference list, including some from the grey literature which might now be more difficult to find unless one knows what to look for. But I would also urge researchers routinely to extend their literature reviews back in time: the exponential increase means that this would not greatly increase the workload as there were relatively fewer papers published the further one goes back.

One example where early work is worth searching out is research and guidance on basic levels of control as an intermediate step, where a lot of work was carried out in the 1970s–1990s. For example, I was disappointed to find the un-caveated suggestion in a recent academic review of hazardous waste management in Africa that any MSW landfill meeting an ‘improved’ or better level of control (Figure 2(b)) could be suitable for some hazardous wastes (Idowu et al., 2019; Akpan and Olukanni, 2020), without any reference to the extensive knowledge base of good (and bad) practice on co-disposal of hazardous wastes in MSWM landfills that could help avoid potentially costly and environmentally damaging mistakes (see Part A).

## Three key policy priorities in waste and resource managment for all countries

My work over recent years on global priorities has highlighted three closely inter-related challenges which must be addressed as policy priorities to shape the continued evolution of WaRM.

### Sustainable financing

Most discussions of sustainable financing for WaRM focus on capital investment costs for full control (ESM) recovery and disposal facilities (see Figure 2(b)). But over a period of years, operational and maintenance costs often exceed investment costs; investment in basic control facilities may be required as an interim step; and collection costs often exceed recovery and disposal costs. So, it is important to consider all aspects of sustainable financing.

#### Securing investment finance for waste facilities

This is a challenge in all countries. Economies of scale ensure that the average facility cost for MSWM is high; according to a commercial database, just over 1000 new recovery and disposal facilities reached implementation in the 6 years 2014–2019, with an average capacity around 230,000 tonnes per annum and investment cost US$ 63 million. The distribution of this new capacity was 18% US and UK, 27% other high-income countries, 38% China, 16% other middle-income countries and just 1% low-income countries (Maalouf et al., 2020). Around three quarters of new capacity is for residual wastes after source separation, mainly for waste to energy; one issue with securing commercial finance in the Global North is that such long-term investments rely on having a guaranteed income stream over 25 years, which is difficult for a municipality when there is the expectation that e.g. targets for waste reduction, reuse and recycling (the 3Rs) will increase over that period.

#### Readiness to absorb investment in the Global South

These data reinforce earlier comments on the problems of financing sustainable waste management in the Global South. Both commercial and official development finance are targeted at those countries who are most able to absorb the investment and service the debt, notably China and other (upper) middle-income countries. For large, mainly high-tech projects, a pre-condition is that a basic, functioning MSWM system (meeting SDG 11.6.1, DB5) is already in place, which, in turn, requires attention to all aspects of governance as discussed in Part B. Indeed, we have proposed that an assessment of a city’s cleanliness, which requires a well-performing MSWM system, could be used as a proxy indicator for ‘good governance’ which is difficult to measure directly (Whiteman et al., 2001). The World Bank has recently set out a roadmap for waste sector ‘reform’ to facilitate evolution of the MSWM system, progressing (using the terminology of this paper) ‘up’ both the 9DB tree (Figure 1) and the ‘ladders’ of collection service level and control level for recovery and disposal (Figure 2). The roadmap was developed using the experience of the EU and Japan, applying that to example countries in eastern Europe and the former Soviet Union (World Bank Group 2018).

#### Basic services are often not affordable

So, the question remains open of how best to make progress in the least developed countries (Bundhoo, 2018), where many cities and towns have difficulty in raising the budgets to cover existing inadequate MSWM services (Brunner and Fellner, 2007). These services need to be improved, to extend collection to all and to upgrade to controlled recovery and disposal (95+% on SDG indicators 11.6.1, development band DB5). The local population need and have the right to better services, to improve public health and the local environment, but often cannot afford to pay (full) cost recovery charges. As a result, the cities are considered to have a low readiness for investment, and strategic projects struggle to be ‘bankable’; so, they have at best restricted access to the funds they require for investing in new infrastructure or operating improved services.

#### The costs are local, the benefits global

However, the continued mismanagement of MSW severely impacts the global environment and the global economy through marine plastics and global heating. The poor local population bear the costs of proper MSWM; but many of the benefits accrue to the rest of the World. How then can this dilemma be solved and the vicious circle broken? It is in the self-interest of, as well as a moral obligation on, the Global North and the international community to assist countries with the least developed MSWM systems to invest in, establish and sustain basic MSWM services for all their citizens.

#### International obligation to deliver sustainable finance

How best can the international community deliver on their obligation to bridge the financing gap between the improved MSWM services and infrastructure which are needed for the global good and what can be afforded locally at the present time? A global environmental and social responsibility initiative is required to finance the rapid expansion of MSWM services to under-served, often low income and/or slum, communities. A broader toolkit of more flexible, innovative and sustainable financing mechanisms needs to be developed, which can be used in multiple combinations and tailored to local circumstances. It is necessary to extend the reach of official development finance to the lowest income countries, and to increase the proportion devoted to SWM (GWMO recommended an increase from the current 0.3% to 3% for 10 years (UNEP and ISWA, 2015)). Use should be made of multiple components, including existing and new financing mechanisms designed to tackle both the climate and plastic pollution emergencies; EPR worldwide (see below); and plastic credits if they can be made effective (PREVENT, 2021; TCI, 2021). Funding also needs to contain a more substantial grant component, recognising the benefit to the wider global community of rapid progress in extending collection to all and eliminating uncontrolled disposal and open burning.

#### Targeting sustainable finance in the Global South

Such sustainable financing mechanisms need to deliver investments in infrastructure AND capacity development, alongside short- and medium-term operational financing, within the framework of a sector policy and/or strategic plan. They need to cover (separate) collection and recycling as well as recovery and disposal; and to ensure that smaller and poorer communities are not left behind. The focus should be on delivery of basic service needs of citizens; generating local business and employment opportunities; maximising waste reduction (expenditure reducing) and reuse, recycling, and recovery (income generating) opportunities; and fostering a healthy environment for the private sector to invest in. These stand in opposition to the quick-fix, technology-fix commercial offers that bombard municipalities in the Global South (see discussion towards end of Part B) and which threaten to stall the development process of the waste and resources sector for a generation.

### Rethink sustainable recycling – Global North

#### The MSW recycling system is broken

Yes, reported recycling rates have increased from low single % figures in the 1980s or 1990s to 40–70% in 2020; but most plastics and paper were exported to the Global South for physical recycling, with the market dominated by China before they effectively stopped the import of post-consumer wastes for recycling in 2018. Several underlying problems all come back to multiple market failures. Product pricing covers the costs of raw materials, manufacturing and distribution, but not the costs of waste management at the end-of-first-life. The market price for recycled materials depends not on the costs of recycling, still less on the complex value of recycled materials including ‘embedded’ technical, social and environmental as well as economic values (Iacovidou et al., 2017, 2021). It fluctuates rather with market price of the virgin materials with which they are expected to compete; to keep the price of their products competitive and maximise profits, the market requires producers to minimise the costs of raw materials, which often favours long-term contracts for virgin materials.

#### Don’t just focus on stimulating supply

Rediscovering MSW recycling in the Global North has vastly increased the supply of materials in world recycling markets, which was met temporarily by an increase in demand from newly industrialising economies in the Global South, primarily China. Policy instruments in the Global North have focused mainly on increasing the recycling capture rates and thus supply, without addressing the fundamental market failure on the demand side. Fixing the broken recycling system will not be easy, requiring actions on multiple fronts. It is critical to allocate responsibilities not with public waste management authorities, but firmly with the producers (see EPR section below). Other components include being prepared to pay for recycling, shifting focus on the supply side from quantity to quality and stimulating demand.

#### Be prepared to pay for recycling

For many years up to the 1980s, MSW recycling in the Global North was an ‘optional extra’, something to be done when market prices were high and (often civil society) organisations could raise some money. With the rediscovery of recycling as a competitive sink to full control (ESM) recovery and disposal options, the mindset that recycling is only undertaken if the income exceeds the expenditure is slowly changing. Separate collection of several segregated fractions for recycling increases collection costs but is now recognised as increasing the collection service level (Figure 2(a)). The willingness to pay for recycling is illustrated by data from the UK, where the change agent WRAP has published an annual report on the gate fees paid by local authorities for various recovery and disposal options since 2008; the current median gate fee paid to a material recycling facility for sorting source separated materials is £60/t gross (range: −£30/t to £135/t), or £18/t net of income from material sales (range: −£155/t to £135/t) (all figures excluding transport costs to the facility) (WRAP, 2022). Most local authorities also reported increasingly stringent limits on input contamination rates (typically <10%).

#### Target quality rather than quantity

Attention needs to shift from a focus on increasing the quantity of materials separated for recycling to increasing the quality (the ‘technical’ value) (Velis and Brunner, 2013), for example through better segregation at source and the separate collection of more fractions. Recycling targets need to be set, not for materials collected for recycling, but for materials actually recycled. Weight targets may need to be supplemented by other metrics; measuring carbon savings (i.e. contribution to global heating mitigation) would increase the focus, for example, on recycling textiles (Turner et al., 2015).

Targets for the more difficult to recycle post-consumer wastes, for example, in MSW, need to be set and monitored separately from those for easier to recycle commercial and industrial wastes. For example, from the viewpoint of local authorities, early implementation in the UK of EU-wide EPR requirements were ineffective, as the producers were able to meet the targets largely by recycling ‘transit packaging’ used to distribute products to supermarkets (Cahill et al., 2011).

Unfortunately, the relevant SDG indicator 12.5.1 has been specified as the national recycling rate, which provides little incentive for individual cities to increase their MSW recycling rates (UNEP, 2021b). The national rate includes ‘clean’ recycling within industry where basic good practice yields high rates (see Part A, 1970 baseline), which can mask continuing low rates for MSW; for example, survey work as part of Hong Kong’s first Waste Reduction Study showed recycling rates around 70% for industrial waste, 30% commercial waste and 9% household waste (ERM, 1994).

#### Stimulate demand

A suite of policies is needed to stimulate demand, including policies on ‘green purchasing’. To take one example, as a self-employed consultant I find myself paying a premium to purchase office quality recycled paper. Demand is low, so the price is higher than for mass produced virgin paper, particularly for small quantities. Two useful policy measures would be to extend producer responsibility to non-packaging paper (including also newspapers and magazines); and to require all large organisations (not just the producers) to specify minimum recycled content in their purchasing decisions. If all public sector organisations specified recycled paper, then the supply of recycled paper would increase to meet the demand, reducing the price and making it easier for private sector and smaller organisation and individuals to follow suit, further increasing the demand; which, in turn, creates the conditions for a stable market for paper collected for recycling.

### Rethink sustainable recycling – Global South

#### Integrate recycling with formal MSWM

In much of the world, recycling still operates as a parallel, largely informal, free market system alongside the formal city MSWM system. So a fundamental component of rethinking recycling in the Global South is to define the system boundary to include at least those informal sector workers who collect and prepare materials for recycling. Such recyclers’ only income is from selling the materials they collect, much of which is sold into international markets; which is in sharp contrast to the Global North, where both collection and preparation for recycling are absorbed within MSWM costs. So, the livelihoods of informal recyclers have suffered both from the market ‘collapse’ triggered by the broken recycling system in the Global North, and more recently from the market ‘hiatus’ caused by COVID-19 (GA Circular, 2020), without any financial ‘safety net’.

I remember well when one client in a technical department arranged a meeting for me with his policy counterpart, to make the case for indirect support to informal recyclers, who could make a living when material prices yielded an income around 8 US$ per tonne of MSW but stopped when the price fell below that level. The cost to the city from collection and disposal was around $80 per tonne, so to me the case for the city to provide land and other indirect support was overwhelming. The cost to the city would have been perhaps $8 per tonne, which would have enabled the recyclers to make a reliable livelihood as recycled material prices fluctuated and so not only continue operating but to double their recycling rate, saving the city $ tens of millions every year. But the answer I got was unequivocal: ‘the city cannot be seen to subsidise the private sector’. That may seem obtuse, but there is still considerable resistance to viewing the recyclers as an integral part of a city’s waste and recycling system, particularly if that involves paying them directly or indirectly for the services they provide in terms of waste collection and recovery.

#### Build from where you are rather than follow the Global North

Another conceptual issue is that the international community expects the Global South to follow the same development path as in the Global North. To quote directly from the World Bank roadmap: ‘*Once full collection coverage and environmentally sound disposal practices are in place, and when affordability allows it, waste separation and recycling should be considered as the next step up in the gradual upgrade of the sector*’ (World Bank Group, 2018). However, this does not take into account three key differences: the existence of an active informal recycling sector in many countries; the challenge highlighted earlier in Part C of ‘running to stand still’ – extending collection coverage and controlled recovery and disposal while (urban) populations and waste generation are growing exponentially; and the very slow progress to date towards SDG indicator 11.6.1 of near universal waste collection and controlled disposal, never mind going beyond that towards ESM.

#### Move earlier to separation at source

So, another aspect of rethinking recycling in the Global South is to make the case for modifying this rigid historical version of the 9DBs roadmap for use in lower-income countries; a question that has been asked, for example, for South Africa (Godfrey and Oelofse, 2017). Can ways be found for the lowest income communities to leapfrog the ‘basic’ level of collection service (Figure 2(a)), moving directly to an ‘improved’ level of service collecting two source segregated fractions, or a ‘full’ level with three or more fractions (Whiteman et al., 2021)? It has often been said to me that asking people to change their behaviour in this way is fine is the Global North, but it won’t work in . . . (their country). I disagree and would offer counter arguments:

a. When services are extended to unserved areas, the baseline behaviour is self-management of wastes, so moving direct to separate collection, for example, of wet and dry fractions, avoids having to change behaviours twice.
b. When affordability is a key issue, it makes sense to maximise the opportunity to valorise the waste by keeping the dry recyclables segregated from the organics so that both can provide income. Separation can even provide a small income for the generator which partially offsets the fee for the collection service.
c. Arguably, the Global South has a significant advantage in that there is often still a customary system of itinerant waste buyers to build on, which was not the case in most of the Global North in 1990. But it is a case of ‘use it or lose it’; my international students often come to me after a lecture and say, ‘I remember the itinerant buyers coming around when I was a child (or my grandmother remembers them well), but we seldom (never) see them now’.

Helping people form or change their behaviours to normalise separation at source (Matter et al., 2013) opens opportunities for existing recyclers to receive better quality dry materials and for recovery of the clean organic fraction in added-value applications. The latter include breeding insects such as black soldier flies as a source of protein and fertiliser; production of char as a cooking fuel; anaerobic digestion to generate gas for cooking; production of high-quality compost suitable for use on food crops; and use as animal feed (for references, see *Part B, Progress in the Global South, 3Rs and the informal sector, Organics recycling*). The benefits should be a win–win–win: extended collection coverage; less waste requiring controlled disposal so less investment required; and more jobs with better working conditions, contributing to reduced poverty (SDG 1) and to decent work and sustainable livelihoods (SDG 8) (Velis et al., 2022).

As an example, one labour-intensive separate waste collection and utilisation system for extending collection to unserved communities has recently been demonstrated in Viet Nam (ZUG, n.d.), and an implementation guide has been prepared (Pfaff-Simoneit, 2023).

### Need worldwide EPR with teeth

#### Cover the full costs

A key part of any solution, for MSWM as a whole and for sustainable implementation of the 3Rs, has to be putting more responsibility on the ‘producers’ (manufacturers and supply chain) who place products or packaging on the market. Where extended producer responsibility (EPR) (or product stewardship) is already present, it needs to be given ‘teeth’ to work more effectively; the producers need to cover all the end-of-first-life costs of collecting, sorting, reusing and recycling their products and of managing any residual wastes; and to meet progressively increasing recycling targets and take on the risk of fluctuating market prices.

#### Incentivise reduction and reuse

But EPR needs to go further; to take packaging as an example, a well-designed EPR scheme must incentivise the waste producer, both to reduce the quantities of single-use packaging and to utilise and/or develop reusable alternatives. The COVID-19 pandemic resulted in a vast increase across the Global North in internet shopping, and thus of single-use packaging wastes. Simply accepting that ‘cardboard can be recycled’ is not enough; ‘proper’ EPR must result in incentives to adopt existing reuse models (e.g. design of boxes to fold up easily (without the use of copious quantities of plastic tape) for collection at the next delivery), and/or to develop new ones.

#### Take full responsibility to make recycling happen

Also, if a product is placed on the market, the producer must take full responsibility to ensure that recycling both can and actually does take place in an environmentally sound (ESM) way. Obligations should include using a minimum % of those recycled materials in manufacturing their products, thus stimulating demand. Such measures should incentivise good design, so that, for example, products can easily be dismantled to facilitate repair, reuse and recycling; the use of difficult-to-recycle materials is minimised; and food-grade packaging materials can be recycled into the same application. Actions to meet EPR obligations might also include investing in development of new recycling technologies, and/or taking an ownership stake in reprocessing facilities.

#### Extend the coverage of EPR

EPR is primarily thought of in the context of packaging wastes and e-wastes, but is much more generally applicable. It has also been applied to batteries, lamps, end-of-life vehicles and tyres; other potential products include clothing and other textiles, nappies (diapers), bulky items such as mattresses, furniture and carpets, selected components of buildings, fishing gear and cigarettes (to control the discarded butts). EPR should cover all products being put on the market which contain chemical substances of concern (see discussion of the circular economy below).

#### Extend EPR upstream

EPR focuses on extending responsibility downstream, to include products at the end of their first life. It is also important that producers should take responsibility for their supply chain; this is usually a voluntary obligation under corporate social responsibility. With globalisation, there has been much recent attention, for example, on wages paid and working conditions at textile factories supplying cheap ‘fast fashion’ brands in the Global North. In the context of this paper, producers need to be held accountable for the waste management practices in their supply chain, including mining, resource extraction and manufacturing. The practice of supply chain hazardous waste audits dates to the 1980s (Wong et al., 1989) and is still good practice.

#### Implement EPR in the Global South

So far, EPR has been implemented mainly in the Global North. But fast-moving consumer goods companies now sell their products in every country, many of which have inadequate MSWM in place; NGOs make a strong case that such companies are directly responsible for the pollution from their mismanaged plastic packaging, much of which is open burned or leaks into the ocean (Tearfund, 2020). Similarly, mobile phones and laptops are now sold worldwide, despite most countries lacking the infrastructure and governance systems to manage e-waste safely (e.g. Omondi et al., 2022). EPR needs to be implemented more generally across the world, in a more coordinated way. It is difficult for many low- and middle-income countries to implement EPR when they would in effect be ‘taking on’ transnational corporations whose annual revenues may dwarf the country’s gross domestic product. A particular challenge in making this work is the current lack of any mechanism for global or even regional EPR; this should be a priority for inclusion in the global treaty on plastics pollution.

More work is required to develop EPR paradigms tailored to the needs of the Global South (see Part B). These should be effective in channelling funds to municipalities and/or their service providers who are struggling to extend waste collection to all. They should also work with the informal recycling sector to achieve progressively increasing recycling targets, thus supporting the gradual inclusion, integration and formalisation of the sector. It is essential to ensure that such relationships are fair and equitable, with the informal sector being able to make a good livelihood in acceptable working conditions; like other actors in the waste and resource management system, they need to be paid for their services, and incentivised to meet targets.

#### Transparent monitoring of EPR

Worldwide, the performance of EPR systems needs to be monitored transparently. Aggregated data on materials placed on the market, collected and recovered need to be made available to the public. There is a strong rationale for EPR organisations to finance data collection initiatives that provide publicly accessible data in a timely manner, and not just data collection to serve their own internal reporting purposes.

## Priority challenges and directions in implementation

### Moving towards clean cycles

#### Directions in the Global North

Some parts of the Global North and China have already brought wastes under control (reaching development band DB5 (Figure 1), meeting SDG indicator 11.6.1), ramped up to full control/(ESM – Figure 2(a)) (DB6/7), and increased collection service levels to separate waste at source (Figure 2(b)) and increase recycling rates (DB8/9). So, their direction of travel is now towards the ultimate aspiration of ‘DB Zero’, sitting on top of the ‘9DBs tree’ (Figure 1), a circular economy or zero waste (Mazur-Wierzbicka, 2021). There are numerous definitions of these terms (Kirchherr et al., 2017), although many are framed around the ‘9Rs’ (see Part B, Edging towards waste prevention; Potting et al., 2017; Morseletto, 2020). The primary focus in moving forward needs to be on the first two groupings of the 9Rs: smarter product use and manufacture (often termed designing out waste (RSA, 2014)); and extend lifespan of product and its parts. Progress to date on what can broadly be termed ‘waste prevention’ has been limited and there is still much to do; many resources are available (e.g. (Ghisellini, et al., 2016; Circle Economy, 2022; Ellen MacArthur Foundation, n.d.; UNEP, n.d.).

Like any aspiration to perfection, it is good to develop strategies and targets to mark progress (e.g. European Commission, 2020), but reaching perfection in an absolute sense is not possible (Brunner, 2013). This is particularly true of the third group of the 9Rs, the useful applications of materials through recycling and recovery (including energy recovery); there will be occasions where materials become so contaminated that they need to escape the cycles of the 9Rs and be disposed of in a safe final sink.

#### Limits to recycling

Modern materials and products include complex mixtures of elements (e.g. metal alloys), with a vast range of chemical additives used to improve both functional and cosmetic properties (e.g. in plastics; Hahladakis et al., 2018). Of the tens of thousands of chemicals in use, only around 500 have been extensively characterised for their hazards and exposures, so the current list of chemical substances of concern will inevitably grow (UNEP, 2019). When materials and products are recycled, careful management is required to prevent cases where the chemicals gradually build up, disperse and/or transform as materials cycle through the economy and become incorporated in new products or enter the environment via uncontrolled emissions (Brunner, 2010; Grosso et al., 2017).

Regulations to control chemicals in products are becoming more stringent in the Global North, for example, the EU *REACH (Registration, Evaluation, Authorisation and Restriction of Chemicals) Regulations*. REACH testing requirements are being phased in, so in principle an existing virgin material containing some undocumented chemicals may be compliant, but when it is recycled into a ‘new’ secondary raw material, that material would require additional testing, which could be an unintended barrier to recycling. It is important to recognise and manage pro-actively any potential policy conflict between recycling and the circular economy on the one hand, and both the public health and environmental protection drivers of waste management (Stanisavljevic and Brunner, 2019), and chemicals legislation aimed at ensuring a non-toxic environment, on the other (Johansson et al., 2020).

As always when there are multiple, conflicting objectives, balance and trade-offs are required. To achieve that requires a collaborative strategy between the regulators, and the materials, chemicals, product manufacturing and waste management sectors. This should include phasing out chemicals of concern at design phase, or when not possible, minimising them and managing potential negative impacts all along their life cycle, including all 9Rs in the circular economy. Consideration should be given to banning chemical additives which are purely cosmetic rather than functional. Accessible, transparent and user-friendly labelling information about the presence of hazardous contents must remain with the product/material as it cycles. When the levels of chemical substances of concern become too high, or include legacy (banned) substances, these need to be removed prior to recycling and the residues disposed to safe final sinks, likely as hazardous wastes.

To help ensure that this happens, the principle of EPR should be expanded to include all products being put on the market anywhere in the world which contain chemical substances of concern.

#### Need for final sinks

The idea of final sinks started with nuclear wastes; the group I joined at Harwell in 1974 was set up to transfer the concept to bringing hazardous wastes under control. For organic wastes, thermal treatment was used to destroy the toxic and persistent organic compounds, concentrating any toxic residuals such as dioxins or inorganic elements in the fly ash remaining after gas cleaning. Some inorganic wastes were chemically treated to destroy their hazards, with, for example, heavy metals concentrated in the residues, solidified and disposed in safe final sinks, either secure hazardous waste landfills (Wilson, 1979a) or in extreme cases, deep underground salt mines (Sierig, 1987). The need for ‘final sinks’ in the context of materials cycling through a circular economy was first pointed out by Brunner (2010), and has since been elaborated (Brunner and Tjell, 2012; Kral et al., 2019).

Making the case for final sinks highlights that the waste hierarchy is a generic order of priority, not an absolute. Reuse and recycling generally sit above energy recovery, thermal destruction and safe landfill, but there are exceptions. For example, spent industrial solvents are hazardous wastes for which the preferred option is solvent recovery for reuse, but the possibility of trace contamination with active ingredients means that waste to energy is preferred for most solvents from pharmaceutical production. Some air pollution control residues from (waste to energy) incinerators can contain trace levels of dioxins and toxic metals such as mercury; in which case, solidification and safe disposal are preferred to recycling into a construction material which would be contaminated.

#### Circular economy in the Global South

Most work on the transition to the circular economy has focused on the Global North, simply because they have already addressed the earlier development bands. The priorities in the Global South are generally still on bringing wastes under control, but the case has already been made here for early consideration of increased collection service levels through source separation, thus creating a win–win–win of extended collection, better livelihoods for the informal recyclers and less waste for controlled disposal within the formal MSWM system. Add in opportunities to build on existing (largely informal) repair and reuse of products, and one could argue that the baseline for building a circular economy is better than in the Global North, but what a circular economy will look like in practice is likely to be quite different.

This is an active area for both research and advocacy. For example, in terms of the wider circular economy, the challenges and opportunities are explored by Ellen MacArthur Foundation (2016), Preston and Lehne (2017), Preston et al. (2019), and the case made by Gower and Schroder, 2016. The role of informal recyclers is explored by Morais et al. (2022) and Velis (2017); and the role of informal reuse, repurposing, repair and recycling by Korsunova et al. (2022). In the context of negotiations on a global plastics instrument, the case is being made strongly for a just transition of the informal waste and recycling sector (UN-Habitat and NIVA, 2022).

### Extending basic waste services to all

#### A continuing challenge

The first sustainable development goal (SDG 1) is: ‘End poverty in all its forms everywhere’; target SDG 1.4 includes ‘Ensure that all men and women, in particular the poor and vulnerable, have equal . . . access to basic services . . .’ (UN, 2015). Indicator SDG 1.4.1 has the following definitions: ‘Basic Services refer to public service provision systems that meet human basic needs including drinking water, sanitation, hygiene, energy, mobility, waste collection, health care, education and information technologies’; and ‘Access to basic services implies that sufficient and affordable service is reliably available with adequate quality’ (UN-Habitat, 2021b). Many of these components are measured by stand-alone indicators, which for waste collection is SDG 11.6.1 (UN-Habitat, 2021a).

For 20 years from 1985, I was involved in many planning and feasibility studies for integrated waste management projects in the Global South, as part of official development programmes. Some of these were implemented, but I also got frustrated when our reports simply ‘sat on the shelf’. When I assessed progress in the GWMO, at least 2 billion people still lacked access to a waste collection service (UNEP and ISWA, 2015), while a recent update increased that estimate to 2.7 billion (see Part B); which I argue constitutes a global waste emergency. These experiences caused me to question whether relying solely on a ‘top-down’ approach led by Governments and cities supported by international finance institutions and bilateral donors can solve the MSWM challenge on its own. The current system favours capital spending on larger infrastructure projects, working preferentially with the more developed (upper) middle-income countries who are seen as more capable of accepting the investment (see Part B).

#### Build from the community upwards

To extend basic collection services to all, I believe that a parallel ‘bottom-up’ approach is required. Which is why when I had the opportunity to commission my CIWM Presidential report, the focus was on helping communities in the poorest countries, where the municipality often has no funds to provide a service, to tackle the problem themselves. The CIWM/Wasteaid toolkit (Lenkiewicz and Webster, 2017; Wilson and Webster, 2018) provides practical guidance on organising community waste management. The focus is on low-cost technologies, which local people can use to make products to sell locally from the low value organics and plastics in the waste – giving themselves a sustainable livelihood to feed and educate their families, while providing a valuable waste collection service for the health and well-being of their community, and the whole planet.

#### A people-centred approach

**Figure 11.**
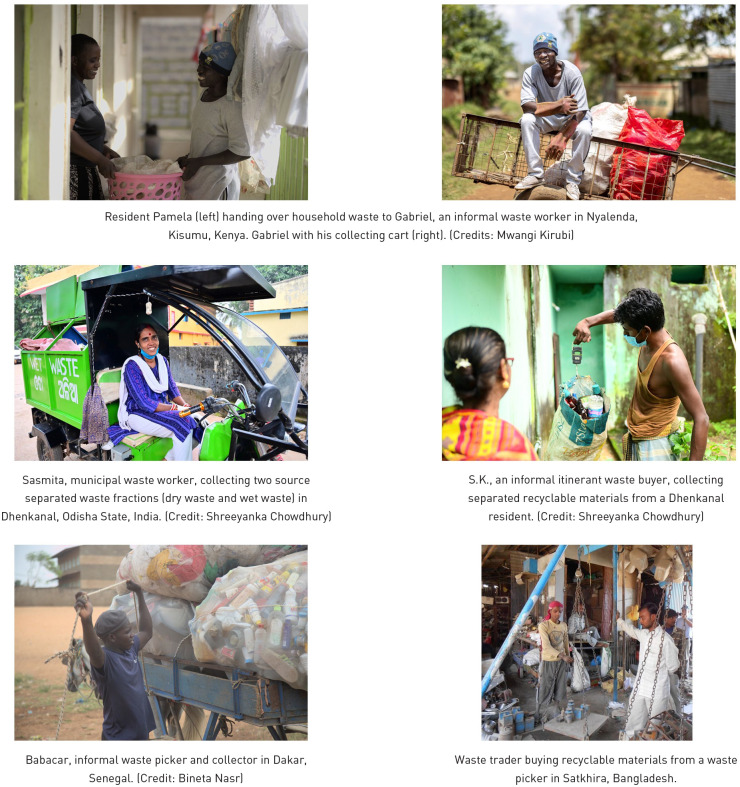
A people-centred approach to MSWM services. Images of residents and professional service providers from both the formal and informal sectors in four case study cities in Africa and South Asia, taken from ‘Managing our Wastes 2021: View from the Global South’ (Practical Action, 2021). ‘*There is an urgent need to bring people back to the heart of the narrative: the impact they suffer and the potential they hold for more effective solutions*’. Most people were happy for their name as well as their picture to be disseminated worldwide. All photos © Practical Action, reproduced with permission. MSWM: municipal solid waste management.

Local NGOs have long adopted such a bottom-up approach, in particular working alongside the informal recycling sector (e.g. Chintan, n.d.; Lardinois and Furedy, 1999; Medina, 2007). Plastics pollution has spurred more international NGOs to become involved in MSWM. I was delighted recently to be invited to endorse a seminal report by the development charity Practical Action, whom I have supported for nearly 50 years. They argue that ‘*Analysis of solid waste management tends to focus on volumes, composition, and flows of waste, and on infrastructure and equipment needed to solve the problem. Recent environmental concerns have reinforced this. There is an urgent need to bring people back to the heart of the narrative: the impact they suffer and the potential they hold for more effective solutions*’. They adapted the collection service ladder (Figure 2(a)) to measure services on four attributes: quality of service, accessibility, impact of waste on the locality, and separation for recycling and resource recovery which bring more value to the poorest in waste value chains. Using evidence from four well-evidenced case studies from Africa and South Asia (Figure 11), they propose refocusing on systems that work for people in terms of affordability, better working conditions and those four attributes of service. Key findings included the following: current low access to even basic services; low focus on waste with the greatest impact (organic wastes, film plastics); informal waste workers make recycling happen; and improving formal collection services does not always support recycling (Practical Action, 2021).

The concept of a people-centred approach to waste and resource management is not new (Ali, 2006), and builds on the broader ISWM components of user and provider inclusivity (see part B); but perhaps its time has now come.

### What standards for recovery and disposal?

#### Upgrading disposal to ‘basic’ control standards

Even when wastes are collected in the Global South, as much as 70% are still disposed of by uncontrolled disposal and open burning (see Part B, using data from Gómez-Sanabria et al., (2022)). So, what more can be done to reach the elusive interim target of 95+% controlled recovery and disposal (SDG 11.6.1 compliance, development band DB4/5)?

One barrier has been the ambiguous attitude to the acceptability of the ‘basic’ level of control. That level was used as a first step in the Global North in the 1970s, but many countries moved directly to increase standards to ‘improved’ and ‘full control’ (environmentally sound management - ESM) levels (Figure 2(b)). It was noted in Part B that some of those countries have considered it wrong for them to fund infrastructure in the Global South that would not meet their own current standards, and may require international finance institutions which they fund to do the same. There is also political pressure in many countries in the Global South to follow ‘best practice’, so ESM is often copied across into national standards. Particularly in secondary cities and rural environs, authorities are unable to afford such high standards, and equally are unable to implement interventions below that standard. So, a combination of international and local insistence on ‘best practice’ is preventing local officials from making affordable incremental improvements and replacing or upgrading existing dumpsites to intermediate basic-control landfills suitable for reaching SDG 11.6.1 compliance. The result is often perverse – in effect an insistence on ‘full control’ allows ‘no control’ uncontrolled disposal and open burning to continue.

This political and cost barrier is magnified further by the economies of scale required to make a full-control sanitary (ESM) landfill economic. In Part B, under Global South investments in infrastructure, 1990s guidance was reviewed on minimum landfill standards and appropriate technology for middle-and low-income countries. Developments since then in landfill design and operation have focused on meeting full control/ESM standards on a large site. So, there remain many gaps when it comes to appropriate and affordable practices and technologies for developing, operating and sustaining intermediate basic-control landfills (which can include relatively basic upgrades to existing uncontrolled sites) to allow SDG 11.6.1 compliance in towns and cities which are either poorer, and/or smaller, and/or more remote and therefore less able to achieve economies of scale through cooperation with neighbouring communities. For example, the lack of a relatively low-cost, versatile, reliable and easy-to-maintain machine for operating a landfill site remains a major barrier to universal rollout of controlled disposal across the world.

#### Is controlled recovery a realistic alternative to controlled landfill?

There are two parts to this question. Under ‘rethink sustainable recycling – Global South’ one part has been addressed, about looking earlier to upgrade collection service levels to separate wastes at source and so enabling higher control levels for both dry materials recycling and for organics recycling/recovery. The answer was both yes, it can and should be brought forward; but no, it reduces but does not remove the need for controlled disposal of the residual wastes.

The second part of the question remains: are there realistic controlled recovery options that would replace the need for controlled disposal of residual wastes to reach SDG 11.6.1/DB5? The conventional answer, in terms of costs and affordability, has generally been ‘No’. The only technology that could potentially offer a cost-effective alternative is co-processing of a refuse-derived fuel fraction in a cement kiln, as cement is manufactured in most countries. But the guidance there (GIZ and LafrageHolcim, 2020), as for all energy recovery technologies, is based on meeting full control (ESM) standards.

A recent WM&R editorial explicitly advocated a step-by-step approach to emission standards, as was done in both the Global North (Part A) and China (Part B), to make waste-to-energy (incineration) more affordable in developing countries (Yan et al., 2020). Chinese manufactured incineration plants already need to meet full control ESM standards for facilities within China; but the argument appears to be both that they should be able to export plants meeting lower emission standards to other developing countries, and that such ‘controlled recovery and disposal’ should be acceptable to meet SDG 11.6.1. However, I would argue that early adoption of waste-to-energy in lower-income countries is unlikely to meet all the pre-conditions set out in current decision-makers’ guidelines (Table 2). In addition, air emissions lead directly to human exposure; and public opposition to incineration is coordinated internationally (GAIA, n.d.); so, any attempt to ‘cut corners’ in this way by accepting intermediate ‘basic’ control standards simply to lower costs is likely to work against the future adoption of waste-to-energy to ESM standards (both in the target country and elsewhere) when the pre-conditions can properly be met and the resulting higher costs are affordable. The relative costs of Chinese manufactured incineration plants appear to be reducing quickly, so that when a city becomes first SDG 11.6.1 compliant and then ready to move to the next step beyond their interim controlled disposal site, a Chinese full control ESM waste-to-energy incinerator might well have become cost competitive with a full control ESM landfill. However, other pre-conditions would still need to be met, including an effective, independent environmental regulator; and facilities for safe disposal of the air pollution control residues, some of which are often classified as hazardous wastes.

#### Moving towards full control standards

It is still the general expectation that the Global South will move from basic to full ESM control levels over recovery and disposal, with the transition being much quicker in upper-middle-income countries. SDG 12.4 made this an explicit target for all wastes by 2020; while global waste target GW3 relaxes the target date to 2030. However, the only official indicators for SDG 12.4 focus on chemicals and on hazardous wastes, so once a city or country reaches basic control over MSW (SDG 11.6.1, DB5), there is no current SDG indicator to monitor further progress. Environmentally sound management (ESM) of MSW appears to have inadvertently ‘fallen between the cracks’ – an omission that needs to be rectified by the new science policy panel on chemicals, waste and pollution prevention.

## Conclusions

### Learning from the past …

This paper has documented the evolution of waste and resource management (WaRM) since the 1970s when environmental control legislation was first introduced. Initial technical standards for municipal solid waste management (MSWM) were similar to ‘basic control’ levels now set in sustainable development goal (SDG) indicator 11.6.1 (Figure 2(b)), corresponding in the nine development bands (9DBs) theory of waste and development to the target baseline DB5 (Figure 1). Standards were increased in a series of steps in the 1980s and beyond, moving through ‘intermediate’ to ‘full control’ or environmentally sound management (ESM), corresponding to pathways via DB6 or DB7. A similar progression was also observed for hazardous wastes.

It is important to remember that waste management as it is today only exists due to effectively enforced legislation. Laws and regulations define basic concepts such as: definitions of waste and hazardous waste; clear allocation of responsibilities and accountability; standards of environmental performance of facilities and operations; and sanctions in cases of non-compliance and violation. The aim is to create a ‘level playing field’, where the waste and resources industry can attract investment in higher standard facilities, without fear of being undercut either by waste criminals or by other facilities operating to lower standards.

By the 1990s, people were beginning to recognise that an approach focussed primarily on the ‘technical fix’, based on technologies within a strictly enforced legislative framework, was not sufficient on its own. In the Global North, new MSW facilities for landfill and incineration to meet ESM standards were becoming increasingly expensive, always met with strong local opposition (not in my back yard – NIMBY) and methane from landfill was recognised as a climate issue. So, recycling of MSW (both dry materials and e.g. composting of the wet, putrescible organics such as food and garden wastes) was being rediscovered, not so much as a source of revenue but more as an alternative, potentially cheaper ‘sink’. Various economic, social and information-based instruments were developed to provide a balanced basket of policy instruments, enabling a more integrated approach to improving the levels of collection service, collecting several source-segregated fractions to facilitate diversion of waste to recycling or more generally ‘up the hierarchy’ (reaching DB8 or DB9). Solid waste management (‘SWM’) was evolving into waste and resource management (‘WaRM’).

Progress in the Global South has generally been much slower. Early attempts to export technologies designed for American, European or Japanese wastes, regulatory systems, cultures and income levels, often resulted in failure. The World Bank and others identified the constraints to taking the first steps of extending MSW collection coverage and controlled disposal (moving gradually from DB1 to DB4) as institutional and financial rather than technical. This led to a new paradigm, *integrated sustainable waste management* (ISWM), which has been used here to analyse progress from the 1990s. ISWM considers both the technical factors, the ‘hard’ components required for physical management of the wastes, the ‘what to do’; and the ‘soft’ governance aspects, required to make that happen in practice, the ‘how to do it’. The latter has been visualised as four interconnecting cogwheels: responsibilities and partnerships (including all stakeholders), money matters (financial sustainability), proactive policies and sound institutions and the need for a data revolution (UNEP and ISWA, 2015).

My current best estimate (albeit still hampered by unreliable data) is that, despite much recent progress, around 2.7 billion people, more than a third of the world’s population, lack access to a waste collection service; while some 40% of MSW that is collected is open dumped or burned (Gómez-Sanabria et al., 2022). This ongoing global waste emergency requires urgent action by the world community.

### . . . in order to plan for the future

Understanding this evolution of waste and resource management (WaRM) over the last 50 years is important when looking forward. The waste problem has absolutely not been ‘solved’. Even in the Global North, much remains to be done, both in terms of bringing all districts, regions and countries up to the same high levels of collection service and of ESM recovery and disposal; and of moving beyond ESM and recycling towards waste prevention and a truly circular economy (‘DB Zero’, Figure 1). There are also additional challenges to be faced, including the emergencies of global heating and plastics pollution, and many ‘new’ waste streams, for example, e-wastes in general, electric car batteries, solar panels, etc., for which both 3rs (reduce, reuse, recycle) and ESM approaches have yet to be (fully) developed. The overriding priority in much of the Global South remains, how to take the essential early steps to bring wastes under control: both by achieving near-universal collection and controlled disposal of MSW, to reach the target baseline of DB5 and 95+% compliance on indicator SDG 11.6.1; and by keeping hazardous wastes separate from MSW and managing them in an environmentally controlled way.

The increasing political profile of WaRM is reflected in the exponential increase in the peer-reviewed literature. Scopus searches for papers on ‘municipal solid waste management’ show around 250 papers up to 1980, increasing by a factor of 2.5–3 each decade, to stand at 14,700 by the end of 2022. My basic thesis is that it is necessary to understand how WaRM has evolved in the past to plan confidently for the future and to avoid ‘reinventing the wheel’. I hope that this paper can contribute by acting as a conduit to earlier experiences, including the ‘grey’ literature; but I would also urge researchers routinely to extend their literature reviews back beyond the current norm of just 5, 10 or 20 years; many more papers were published recently, so this would not greatly increase the workload.

### Priorities moving forward

#### Three policy priorities

Three closely inter-related policy priorities are critical to the continued evolution of waste and resource management (WaRM) in all countries. One is sustainable financing, which remains a challenge for residual waste management facilities in the Global North, particularly in countries like the UK and the United States which rely more on market forces (development pathway DB6 → DB8). In much of the Global South, the financial costs of better MSWM are unaffordable locally, but the economic benefits of reducing plastics pollution and global heating are felt worldwide; a global initiative is required to develop tailored and innovative financing mechanisms to extend services to under-served communities.

The second priority is a radical rethinking of sustainable recycling of MSW. In the Global North, the MSW recycling system, which has been rebuilt from a low base over the past 30 years, is broken. The focus needs to move from increasing the supply of materials as measured by the quantities collected for recycling, to increasing demand and the quality and quantity of materials actually recycled. Targets need to be set not just on weight, but also on carbon.

In the Global South, the baseline is quite different, with an active informal sector recycling system often operating in parallel to the formal city MSWM system. Here, radical rethinking means first combining existing formal MSWM and informal recycling into one integrated WaRM system and involving the existing informal recyclers as a key stakeholder group. Second, modifying the 9DBs roadmap as followed in the Global North to leapfrog the ‘basic’ level of collection service (Figure 1), moving directly to an ‘improved’ or ‘full’ level of service collecting two, three or more source segregated fractions. The benefits should be a win–win–win: extended collection coverage; less waste requiring controlled disposal so less investment required; and more jobs with better working conditions for both formal and informal waste workers.

The third, inter-related, policy priority is to place more responsibility on the ‘producers’ (manufacturers and supply chain) who place products or packaging on the market, including all products containing chemical substances of concern. Where extended producer responsibility (EPR) (or product stewardship) is already present, it needs to be given ‘teeth’ to work more effectively. The producers need to: cover all the end-of-first-life costs of collecting, sorting and recycling their products and of managing any residual wastes; meet progressively increasing recycling targets; and take full responsibility to ensure that recycling both can and actually does take place in an environmentally sound (ESM) way. A well-designed EPR scheme must incentivise product design to reduce waste quantities; to facilitate disassembly for easy repair, reuse and recycling; and to minimise the use of difficult-to-recycle materials.

EPR needs to be implemented in a coordinated way across the world; it is the municipalities and informal recyclers in the Global South who are most in need of financial and other support from the producers to manage end-of-life products and packaging on their behalf, and who are most impacted by their mismanagement.

#### Extending waste services to all

Implementation of these three priority policies would go some way to addressing the global waste emergency of billions of people still without even basic MSWM services. However, the traditional ‘top-down’ approach to development, working through national governments, slowly building capacity and focusing on (larger) investments in infrastructure, will take many years to reach the poorest communities. So a parallel, complementary, ‘bottom-up’ approach is also required, working with NGOs and communities themselves, and putting people at the centre of the narrative. Sustainable waste and resource management needs to work for the poorest people, providing both a quality service which keeps low-income and slum areas clean and healthy, and a decent livelihood for the multitude of workers who deliver collection and recycling services.

Achieving controlled disposal of residual wastes at an affordable cost remains a major challenge; not least because the priority of most people is to keep their immediate neighbourhood clean (‘out of sight, out of mind’), so local willingness to pay for disposal is limited. Innovation is required in governance, service delivery and technologies used. Solutions need to meet what have been termed the ‘Pent As’: institutionally Appropriate, technically Applicable, legally Achievable, financially Affordable, and environmentally Acceptable (See part B, investing in infrastructure). The approaches used would be customised to the context, needs and capacity of the target community, to make the system more resilient to external shocks (financial crisis, health pandemic, fuel supplies, etc.), with a strong focus on participative planning and the various ISWM governance aspects.

#### An entry point to wider global goals

Getting waste and resource management right will also contribute to many other societal issues. We previously proposed that a clean city, which requires a well-functioning MSWM system, could be used as a proxy indicator for good governance (Whiteman et al., 2001). Addressing the five global waste targets identified in the Global Waste Management Outlook (GWMO) would contribute directly to 12 of the 17 SDGs (Wilson, 2021). Achieving near-universal MSW collection and controlled recovery and disposal (SDG 11.6.1, DB5) was estimated earlier to cut by half the weight of plastics entering the ocean, and to eliminate the scourge of open burning. Add to that substantial progress on the 3Rs (reduce, reuse, recycle), including food waste prevention, and it can be said with high confidence that better waste and resource management could make a substantial contribution to climate mitigation (perhaps 15–20% of global GHG emissions).

### New opportunities

With challenges come opportunities. I have worked for many years to increase the political priority of waste and resource management and have often felt that I was fighting a losing battle. Then in March 2022, the fifth UN Environmental Assembly passed two resolutions which could potentially change that. ‘End Plastic Pollution: Towards an international legally binding instrument’ (UNEA, 2022a) puts waste and resource management at the centre of what needs to become massive change (Siva Filho and Velis, 2022). The second looks to establish an international science-policy panel on chemicals, waste and pollution prevention, modelled on the IPCC for climate change (UNEA, 2022b). Hopefully, the panel’s scope will routinely include hazardous wastes, plastic pollution and wastes, open burning and improving data; but that list must also include improving MSWM in developing countries (achieving 95+% on SDG 11.6.1) and moving towards the 3Rs/9Rs/circular economy.

When I first stumbled into waste management in 1974, I did not expect still to be here nearly 50 years later. I have stayed because my international work on policy, planning and the evidence base has constantly evolved, bringing interesting new challenges and opportunities. Thanks largely to plastics pollution, those are greater now than ever; the early 2020s may be a ‘tipping point’, when my ‘baby’ has ‘come of age’ and is emerging at last onto the world stage as a global priority. But I do need to continue handing on the baton to the next generations of waste and resource managers and researchers.

## Appendix

### List of abbreviations

3Rs Reduce, reuse, recycle

9DBs Nine development bands theory of waste and development (Whiteman et al., 2021)

9Rs A common definition used for the circular economy, sub-dividing and refining the 3Rs (Potting et al., 2017; Morseletto, 2020)

AR5/6 IPCC’s Fifth (IPCC, 2013) and Sixth (IPCC, 2021) Assessment Reports

CIWM UK Chartered Institution of Wastes Management

CWG Collaborative Working Group on MSWM in low- and middle-income countries

DBs Development bands (see also 9DBs above)

EC European Community (predecessor of the EU)

EPR Extended producer responsibility

ESM Environmentally sound management

EU European Union

e-waste Electronic waste (also known as waste electrical and electronic equipment or WEEE)

GHG Greenhouse gases

GW1, GW2 etc Global waste targets, corresponding to a ‘virtual waste SDG’

GWMO Global Waste Management Outlook (UNEP and ISWA, 2015)

GWP Global warming potential compared to CO_2_

hazwaste Hazardous waste

IPCC Intergovernmental Panel on Climate Change

ISWA International Solid Waste Association

ISWM Integrated sustainable waste management

MSW Municipal solid waste

MSWM Municipal solid waste management

Mtpa Million tonnes per annum

NGO Non-governmental organisation

NIMBY Not in my back yard

PPP Public–private partnership

PSP Private sector participation

SDG Sustainable development goal

UMP Urban Management Programme (joint activity of UNDP, UN-Habitat & World Bank)

UN United Nations

UNDP UN Development Programme

UNEA UN Environment Assembly

UNEP UN Environment Programme

UNFCCC UN Framework Convention on Climate Change

WABI Wasteaware Benchmark Indicators (Wilson et al., 2015a)

WaCT Waste Wise Cities Tool (UN-Habitat, 2021a)

WaRM Waste and resource management

WaW The World Bank’s *What a Waste* report (Hoornweg and Bhada-Tata, 2012).

Also the second edition WaW2.0 (Kaza et al., 2018)

WGHW ISWA Working Group on Hazardous Waste

WHO World Health Organisation

WM Waste management

WM&R Waste Management & Research journal
